# The Rise and Fall of Billionaire siRNAs during Reproductive Development in Rice

**DOI:** 10.3390/plants11151957

**Published:** 2022-07-28

**Authors:** Lili Wang, Dachao Xu, Longjun Zeng, Dong-Lei Yang

**Affiliations:** 1 State Key Laboratory of Crop Genetics and Germplasm Enhancement, Nanjing Agricultural University, Nanjing 210095, China; 2014201023@njau.edu.cn (L.W.); 2015201060@njau.edu.cn (D.X.); 2 Yichun Academy of Sciences, Yichun 336000, China; longjunzeng@126.com

**Keywords:** siRNA, rice, reproductive development

## Abstract

RNA polymerase IV-dependent siRNAs, usually 24 nt in length, function in the RNA-directed DNA methylation that is responsible for de novo methylation in plants. We analyzed 24 nt siRNAs in inflorescences and found that among the 20,200 24 nt siRNA clusters, the top 0.81% highly expressed clusters accounted for more than 68% of the 24 nt siRNA reads in inflorescences. We named the highly expressed siRNAs as billionaire siRNAs (bill-siRNAs) and the less-expressed siRNAs as pauper siRNAs (pau-siRNAs). The bill-siRNAs in inflorescences are mainly derived from the ovary. Female gametes produced more bill-siRNAs than male gametes. In embryos and seedlings developed from fertilized egg cells, the bill-siRNAs from gametes disappeared. The endosperm, which develops from the fertilized central cell, also contained no bill-siRNAs from gametes but did contain newly and highly expressed siRNAs produced in different regions. In contrast, bill-siRNAs from the ovaries were maintained in the seed coat. The biosynthesis of bill-siRNAs in various tissues and cells is dependent on OsRDR2 (RNA-dependent RNA polymerase 2) and Pol IV (DNA-dependent RNA polymerase IV). Similar to the pau-siRNAs, the first base of bill-siRNAs is enriched at adenine, and bill-siRNAs can direct DNA methylation in various tissues.

## 1. Introduction

In plants, small RNAs (sRNAs) are usually 21–24 nucleotides (nt) long and regulate almost all biological processes, including development, adaptation to stress, and genome stability. sRNAs regulate gene expression at both the transcriptional and posttranscriptional level. Based on their biogenesis and functions, sRNAs can be categorized into two major groups in plants: microRNAs and small interfering RNAs (siRNAs) [1]. siRNAs can be further categorized into p4-siRNAs, phased small interfering RNAs (phasiRNAs), epigenetically activated siRNAs (easiRNAs), and natural antisense transcript-derived siRNAs (nat-siRNAs). P4-siRNAs are usually 24 nt long and are transcribed by Pol IV-RDR2 on TEs and repeats [2,3,4].

The molecular mechanism of RdDM has been well studied in Arabidopsis, and the mutations in most components of RdDM-machinery genes cause few developmental defects in Arabidopsis [5,6]. *osnrpd1a/osnrpd1b* double mutants, however, affect panicle size and tiller number in rice [7,8]. The *pol iv* mutant in Arabidopsis can rescue triploid embryo abortion [9]. *Capsella pol iv* affected male gamete development [10]. In *fem1* (*osrdr2*) mutants of rice, the development of both male and female reproductive organs was defective [11]. Together, these results indicate that 24 nt siRNAs are important for reproductive development of rice and other plants.

Grover et al. [12] compared the cumulative distribution of RNA loci in unfertilized ovules, seed coats, and leaves in *Brassica rapa* and found that siRNA were concentrated on minor loci in ovules and seed coats. They called the highly expressed siRNA regions “siren loci”. Here, we identified 163 bill-siRNA clusters among 20,200 24 nt siRNA clusters in inflorescences, and found that although they represented only 0.81% of the 24 nt siRNA clusters, the bill-siRNA clusters produced more than 68% of the 24 nt siRNAs in rice. The bill-siRNAs in inflorescences were mainly produced in the ovary, in which both the egg cell and ovary without the egg cell produced bill-siRNAs. Both sperm and vegetative cells produced bill-siRNAs at different regions within the ovary. In both the embryo and endosperm, the gamete bill-siRNAs were absent. In the endosperm, however, new bill-siRNAs were produced at different loci. Although bill-siRNA clusters are much longer than pau-siRNA clusters, the two kinds of P4R2-siRNAs have a similar bias for the first base and the same function in mediating DNA methylation.

## 2. Results

### 2.1. Identification of Billionaire siRNAs in the Rice Inflorescence

In a previous study, we found that the whole-genome methylation level (WGML) in the CHH context was higher in inflorescences than in seedlings mainly because of the up-regulation of RdDM machinery [11]. However, 24 nt siRNA abundance on the hyper-methylated differentially methylated regions (hyper-DMRs) of inflorescence relative to seedlings was significantly lower in inflorescences than in seedlings (Figure S1A). The inconsistency between the increase of CHH methylation levels and the decrease in siRNA abundance in inflorescences relative to seedlings led us to analyze 24 nt siRNAs in detail. Using the same cutoff (see Methods), we identified 57,424 24 nt siRNA clusters in seedlings but only 20,200 in inflorescences, indicating that 24 nt siRNAs were reprogramed in inflorescences relative to seedlings. About 90.2% of inflorescence siRNA clusters were also identified in seedlings, and only 1971 clusters were inflorescence-specific (Figure S1B). On all seedling siRNA clusters (siRNA clusters that were seedling-specific or common to seedlings and inflorescences), the siRNA abundance was higher in seedlings than in inflorescences, but the CHH methylation was lower in seedlings than in inflorescences ([11]; Figure S1B). By contrast, the 24 nt siRNA abundance and CHH methylation were both higher in inflorescences than in seedlings on the inflorescence-specific siRNA clusters (Figure S1B).

**Figure 1:**
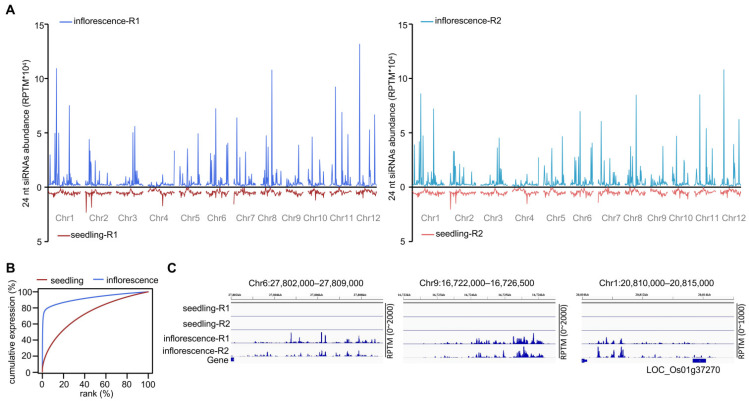
Rice inflorescences produce bill-siRNAs. (**A**) Comparison of 24 nt siRNA distributions on the rice genome between inflorescences and seedlings in Nipponbare. Y-axis indicates siRNA abundance with RPTM within 500 kb windows. (**B**) Cumulative expression plot of 24 nt siRNAs in inflorescences and seedlings in Nipponbare. The abundance of each cluster is indicated by RPTM based on two replicates; their cumulative expression was ranked and plotted. siRNA clusters were analyzed if their abundance ≥ 12 RPTM in combined replicates (n = 20,200 in inflorescence, n = 57,424 in seedling). (**C**) Screen shots of Integrative Genomics Viewer (IGV) showed the abundance of bill-siRNAs in inflorescences and seedlings.

The negative correlation between siRNA abundance and CHH methylation level on hyper-DMRs of inflorescence relative to seedling led us to investigate 24 nt siRNA distribution on the genome in Nipponbare inflorescences and seedlings. siRNA abundance in seedlings was roughly evenly distributed on the genome but was concentrated in certain regions in inflorescences (Figure 1A). Cumulative expression analysis of 24 nt siRNAs in inflorescences and seedlings revealed that a few regions produced most of the siRNAs in inflorescences (Figure 1B). We attempted to sort the highly expressed 24 nt siRNA clusters in inflorescences by their abundance. siRNA clusters with reads per ten million (RPTM) > 500, >1000, >1500, >1600, and >2000 accounted for 75.0, 72.7, 70.6, 70.1, and 68.7% of all 24 nt siRNA reads, respectively. To strictly define the highly expressed siRNA clusters, we used RPTM > 2000. We found only 163 siRNA clusters with RPTM > 2000 (Table S1), which represented <0.81% of all 24 nt siRNA clusters in inflorescences. On these clusters, however, the siRNAs accounted for 68.7% of the 24 nt siRNA reads in inflorescences. In contrast, using the same criterion (RPTM > 2000), we found only 19 highly expressed siRNA clusters in seedlings (Table S1). On these clusters, the siRNAs accounted for 3.1% of siRNA reads in seedlings. Because of their similarity to the divide between the rich and poor in modern human society, we named these highly expressed siRNA clusters “billionaire-siRNA clusters” (bill-siRNA clusters). The other 24 nt siRNA clusters were accordingly named pauper-siRNA clusters (pau-siRNA clusters). The abundance of 24 nt siRNAs on bill-siRNA clusters was much higher in inflorescences than in seedlings (Figure 1C).

To investigate the conservation of bill-siRNAs in rice populations, we conducted sRNA sequencing for inflorescences and seedlings in the TP309 variety (Table S2). As was the case in Nipponbare inflorescences, the 24 nt siRNAs in two replicates of TP309 inflorescences were concentrated in certain regions of the genome (Figure S2A). Based on cumulative expression analysis, the 24 nt siRNAs were concentrated in a small number of clusters in TP309 inflorescences (Figure S2B). Using the same criterion that was used for Nipponbare, we identified 129 bill-siRNA clusters in TP309 inflorescences (Table S1), which represented only 0.5% of the 23,635 siRNA clusters in TP309 inflorescences. On these clusters, however, the siRNAs accounted for 65.2% of all 24 nt siRNA reads. In TP309 seedlings, there were only 12 bill-siRNA clusters (among a total of 46,743 siRNA clusters), which accounted for 2.5% of 24 nt siRNA reads. As was the case with Nipponbare, the abundance of 24 nt siRNAs on bill-siRNA clusters in TP309 was much higher in inflorescences than in seedlings (Figure S2C).

A Venn diagram showed that 96.1% of bill-siRNA clusters in TP309 inflorescences overlapped with those in Nipponbare inflorescences (Figure S3A,B), indicating that most of the bill-siRNA clusters are probably conserved in natural populations of rice. There were 39 Nipponbare-specific and 5 TP309-specific bill-siRNA clusters (Figure S3A,C,D). The siRNA levels on Nipponbare-specific bill-siRNA clusters were significantly higher in Nipponbare inflorescences than in TP309 inflorescences (Figure S3A,C). In both varieties, the siRNA levels were significantly higher on common bill-siRNA clusters than on variety-specific clusters (Figure S3E). On common and variety-specific bill-siRNA clusters, SNP and Indel number did not significantly differ between Nipponbare and TP309 (Figure S3F), indicating that the production of variety-specific bill-siRNAs can probably not be explained by sequence variation.

### 2.2. Features of Bill-siRNAs

**Figure 2:**
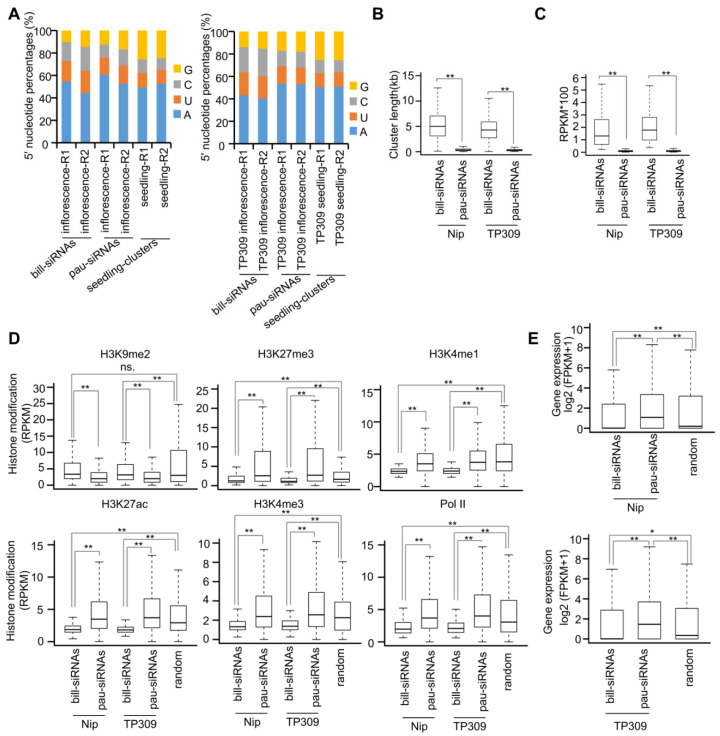
Characteristics of bill-siRNAs in inflorescences. (**A**) Percentage of four bases on the first nucleotide acid of bill-siRNAs and pau-siRNAs. (**B**) Lengths of bill-siRNA clusters and pau-siRNA clusters in Nipponbare and TP309. ** indicates *p* < 0.01 by the Wilcoxon sum test. (**C**) Relative abundance of bill-siRNA clusters and pau-siRNA clusters in Nipponbare and TP309. ** indicates *p* < 0.01 by the Wilcoxon sum test. (**D**) Comparison of histone modification of H3K9me2, H3K27me3, H3K4me1, H3K27ac, and H3K4me3, and Pol II occupancy on bill-siRNA clusters and on pau-siRNA clusters. A total of 20,000 randomly selected 350 bp regions were analyzed and served as control. ** indicates *p* < 0.01, and ns indicates not significant by the Wilcoxon sum test. (**E**) Comparison of expression level of bill-siRNA cluster- and pau-siRNA cluster-adjacent genes in Nipponbare and TP309. Three hundred randomly selected genes served as controls. ** indicates *p* < 0.01, and * indicates *p* < 0.05 by the Wilcoxon sum test.

The first base was enriched at adenine for bill-siRNAs in inflorescences (Figure 2A), which is also true for 24 nt siRNAs in seedlings and for pau-siRNAs in inflorescences, suggesting that both bill-siRNAs and pau-siRNAs are loaded into AGO4 [13]. We found that bill-siRNA clusters were significantly longer than pau-siRNA clusters in both varieties (Figure 2B). Even when the siRNA abundance is divided by the cluster length, the siRNA abundance was much higher on bill-siRNA clusters than on pau-siRNA clusters (Figure 2C). Using previously published ChIP-seq data for various histone modifications in Nipponbare inflorescences [14], we found that the levels of H3K9me2, a repressive histone mark, were significantly higher on bill-siRNA clusters than on pau-siRNA clusters (Figure 2D). In contrast, the levels of H3K27me3, another repressive histone mark, were significantly lower on bill-siRNA clusters than on pau-siRNA clusters (Figure 2D), suggesting that bill-siRNAs tend to locate on stable heterochromatin but not on temporary heterochromatin.

In addition, active histone marks (H3K4me1, H3K4me3, and H3K27ac) were less enriched on bill-siRNA clusters than on pau-siRNA clusters (Figure 2D). Consequently, RNA polymerase II was less occupied on bill-siRNA clusters than on pau-siRNA clusters (Figure 2D). Eventually, the expression levels were significantly lower for bill-siRNA cluster-adjacent genes than for pau-siRNA cluster-adjacent genes (Figure 2E).

Only 12.6% and 15.9% of the genes that overlapped with bill-siRNA clusters exhibit differential expression (fold change > 2, FDR < 0.05) between seedlings and inflorescences in Nipponbare and TP309, respectively (Figure S4A,B), suggesting that bill-siRNA might have little effect on gene transcription of adjacent genes.

### 2.3. Accumulation of Bill-siRNAs Is Dependent on OsRDR2 and Is Required for CHH Methylation

**Figure 3:**
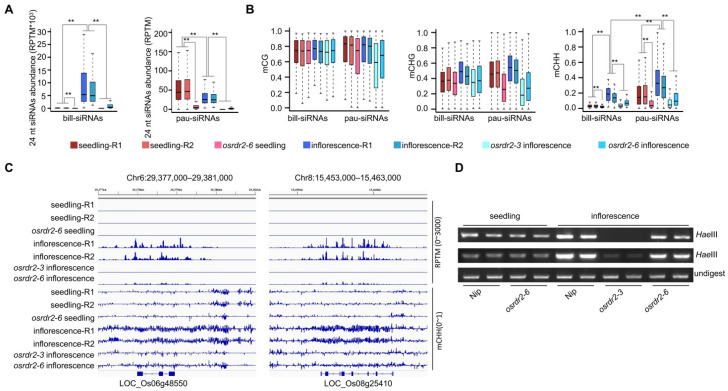
Accumulation of siRNAs and CHH methylation on bill-siRNA clusters depend on OsRDR2. (**A**) Boxplots showing the 24 nt siRNA abundance in seedlings and inflorescences of WT and *osrdr2* mutant. ** indicates *p* < 0.01 by the Wilcoxon sum test. (**B**) Boxplots showing the methylation levels of CG, CHG, and CHH in seedlings and inflorescences of the WT and *osrdr2* mutant on bill-siRNA clusters and pau-siRNA clusters in inflorescences. ** indicates *p* < 0.01 by the Wilcoxon sum test. (**C**) Screen shots of IGV indicating 24 nt siRNA abundance and CHH methylation levels in seedlings and inflorescences of the WT and *osrdr2* mutant on two representative bill-siRNA clusters. (**D**) Chop-PCR assay suggesting the CHH methylation level in seedlings and inflorescences of the WT and *osrdr2* mutant.

On bill-siRNA clusters, siRNA abundance was substantially higher in inflorescences than in seedlings (Figure 3A). On pau-siRNA clusters, in contrast, siRNA abundance was significantly lower in inflorescences than in seedlings (Figure 3A). The predominant production of siRNAs on bill-siRNA clusters is the reason for the lower siRNA abundance on pau-siRNA clusters in inflorescences than in seedlings. The siRNA on both bill-siRNA and pau-siRNA clusters was depleted in both inflorescences and seedlings of *osrdr2* (Figure 3A).

Because Pol IV- and RDR2-generated siRNAs are the hallmarks of RdDM [4,15], we assessed CHH methylation on siRNA clusters. On both bill-siRNA and pau-siRNA clusters, the CHH methylation levels were significantly higher in inflorescences than in seedlings (Figure 3B), which is consistent with our previous finding that CHH methylation is globally higher in reproductive organs than in seedlings [11]. The CHH methylation on bill-siRNA and pau-siRNA clusters is significantly lower in *osrdr2* inflorescences than in wild-type (WT; Nipponbare) inflorescences (Figure 3B), demonstrating that *OsRDR2*-dependent bill-siRNAs as well as *OsRDR2*-dependent pau-siRNAs are required for DNA methylation in inflorescences. Even though siRNA abundance is much higher on bill-siRNA clusters than on pau-siRNA clusters, the CHH methylation levels are lower on bill-siRNA clusters than on pau-siRNA clusters in inflorescences (Figure 3B), indicating that siRNA abundance is not positively correlated with the levels of DNA methylation. We selected two bill-siRNA clusters (Figure 3C) and subjected them to Chop-PCR. The Chop-PCR assay demonstrated that CHH methylation is higher in inflorescences than in seedlings, and that the high CHH methylation in inflorescence is dependent on *OsRDR2* (Figure 3D).

### 2.4. Bill-siRNAs in Inflorescences Are Mainly Derived from the Ovary

**Figure 4:**
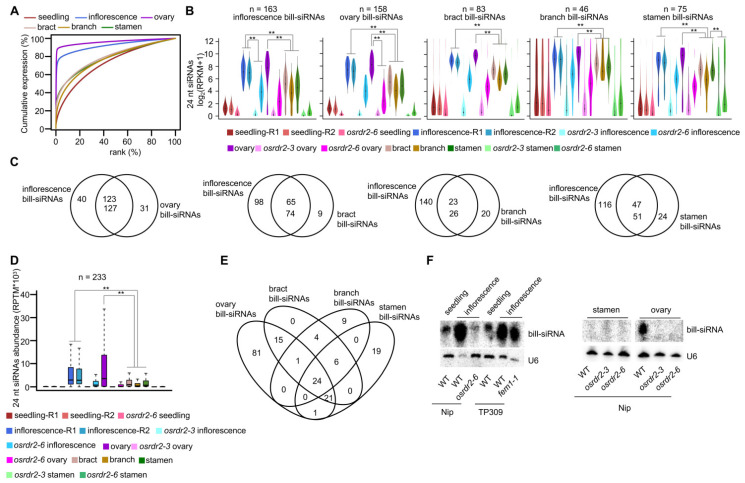
Bill-siRNAs in inflorescences are mainly derived from the ovary. (**A**) Cumulative expression plot of 24 nt siRNA clusters in seedlings, inflorescences, ovaries, bracts, branches, and stamens in Nipponbare. (**B**) Violin plots showing 24 nt siRNA abundance in different tissues on various genotypes on various bill-siRNA clusters. Quantile lines indicate the 1/4 and 3/4 percentiles. Black dots represent the median. ** indicates *p* < 0.01 by the Wilcoxon sum test. (**C**) Venn diagram indicating the overlap between bill-siRNAs in inflorescences and bill-siRNA clusters in the other four tissues. (**D**) Boxplot showing 24 nt siRNA abundance on the combined bill-siRNA clusters (inflorescences, ovaries, bracts, branches, and stamens) in various tissues. ** indicates *p* < 0.01 by the Wilcoxon sum test. (**E**) Venn diagram indicating the overlaps between bill-siRNA clusters in four tissues. (**F**) sRNA northern blotting indicating the sRNA levels of different tissues on one representative bill-siRNA cluster.

The rice inflorescence is composed of branch, lemma/palea (bract), ovary, and stamen. To clarify the source of bill-siRNAs in the inflorescence, we conducted sRNA sequencing for those tissues at development stage 12 of the anther (Table S2). Cumulative expression analysis indicated that the accumulation of siRNAs in the ovary was very similar to that in the inflorescence (Figure 4A and Figure S5). The siRNAs tend to be concentrated on a small number of loci in the branch, bract, and stamen, but were less concentrated in those tissues than in the inflorescence (Figure 4A). The siRNAs in the ovary, however, were even more concentrated than in the inflorescence (Figure 4A and Figure S5). Using the same criterion that we used for the inflorescence, we identified 158 bill-siRNA clusters in the ovary, 83 in the bract, 46 in the branch, and 75 in the stamen (Table S1).

On bill-siRNA clusters, siRNA abundance is similar in the ovary and the inflorescence, but is significantly higher in the ovary than in the bract, branch, or stamen (Figure 4B). In addition, 75.5% of bill-siRNA clusters in the inflorescence are shared by the bill-siRNA clusters in the ovary. In contrast, a much lower percentage of bill-siRNA clusters in the inflorescence are overlapped with bill-siRNA clusters in the bract, branch, or stamen (39.9, 14.1, or 28.8%, respectively; Figure 4C), suggesting that the majority of bill-siRNAs in the inflorescence are derived from the ovary.

Among the five tissues, there are a total of 233 bill-siRNA clusters. Among these 233 siRNA clusters, 24 nt siRNA abundance is highest in the ovary (Figure 4D), and is significantly lower in the bract, branch, and stamen than in the inflorescence or ovary (Figure 4D). Of the bill-siRNA clusters in the bract, branch, and stamen, 85.92, 56.82, and 64.79%, respectively, are overlapped with those in the ovary (Figure 4E). Together, these results indicate that most of the bill-siRNAs in the inflorescence are produced in the ovary. On the 233 bill-siRNA clusters in the five tissues, siRNA accumulation was significantly reduced in the *osrdr2* mutant (Figure 4D), suggesting that the biosynthesis of bill-siRNAs is dependent on *OsRDR2*. On one representative bill-siRNA cluster, northern blotting confirmed that *OsRDR2* is required for bill-siRNA production in the inflorescence and the ovary (Figure 4F).

On pau-siRNA clusters of the ovary and stamen, siRNA accumulation was significantly lower in *osrdr2* than in the WT (Figure S6A,B), suggesting that the biosynthesis of both bill-siRNAs and pau-siRNAs is dependent on *OsRDR2* in the ovary and stamen. The CHH methylation level on bill-siRNA clusters in the ovary and stamen was substantially lower in *osrdr2* than in the WT, suggesting that bill-siRNAs are functional in directing de novo DNA methylation in the ovary and stamen (Figure S6C,D).

As in the inflorescence, the first bases of bill-siRNAs in the ovary, bract, branch, and stamen are enriched on adenine (Figure S7A). The bill-siRNA clusters were significantly longer than the pau-siRNA clusters in the same tissues (Figure S7B). The expression levels of the genes that overlapped with bill-siRNA clusters are significantly lower than those that overlapped with pau-siRNA clusters in both the ovary and stamen (Figure S7C,D). Only 14.2% of the genes that overlapped with bill-siRNA clusters in the ovary show differential expression between the ovary and seedling (fold change > 2, FDR < 0.05), and 1.9% of the genes that overlapped with ovary bill-siRNA clusters in the ovary change their transcriptional levels when bill-siRNAs are eliminated in the *osrdr2* ovary (Figure S7E,F). Moreover, 16.4% of the genes that overlapped with bill-siRNA clusters in the stamen exhibit differential expression between stamen and seedlings, and only 1.8% of them change their transcriptional levels when bill-siRNAs are eliminated in the *osrdr2* stamen (Figure S7G,H), indicating that bill-siRNAs have little role in regulating adjacent gene expression.

### 2.5. Bill-siRNAs in Gametes

Because bill-siRNAs are abundant in the ovary, we considered the possibility that bill-siRNAs are highly expressed in the egg cell. To test this possibility, we analyzed sRNAs of the egg cell and of the ovary without the egg cell [16]. The genomic distribution of siRNAs was similar in the ovary with egg, the egg cell alone, and the ovary without the egg cell (Figure S8A,B). A cumulative expression plot showed that the concentration of siRNAs in the egg cell and the ovary without the egg cell was comparable to that in the ovary with the egg cell (Figure S8C). We identified 168 and 185 bill-siRNA clusters in the egg cell and in the ovary without the egg cell, respectively. Among the 168 bill-siRNA clusters in the egg cell, 160 (95.2%) are overlapped with those in the ovary (Figure S8D). Among 185 bill-siRNA clusters in the ovary without the egg cell, 147 (79.5%) are overlapped with those in the ovary with the egg cell (Figure S8E). Among 168 bill-siRNA clusters in the egg cell, 165 (98.2%) are overlapped with those in the ovary without the egg cell (Figure S9). These results indicated that the bill-siRNAs are equally produced in the egg cell and other cells of the ovary.

As is the case in the ovary and inflorescence, bill-siRNA clusters are longer than pau-siRNA clusters in the egg cell and in the ovary without the egg cell (Figure S10A). The first base of bill-siRNAs in the egg cell and in the ovary without the egg cell is enriched for A (Figure S10B), which is similar to the bill-siRNAs in the inflorescence and ovary.

We used sRNAs of the sperm and vegetative cell [16] to identify potential bill-siRNAs in the male gamete. Cumulative expression plots revealed that siRNA distribution in the sperm and vegetative cell is similar to siRNA distribution in the stamen (Figure S11A). Using the same criterion, we identified 65 and 18 bill-siRNA clusters in the sperm and vegetative cell, respectively. Among the 18 bill-siRNA clusters of the vegetative cell, 15 are overlapped with those of the sperm (Figure S11B). On bill-siRNA clusters in the sperm cell, siRNA abundance is significantly higher in the sperm cell than in the vegetative cell or stamen (Figure S11B). In addition, the majority of bill-siRNAs of sperm were specific to the sperm relative to the stamen (Figure S11C). By contrast, a majority of bill-siRNAs of the vegetative cell are shared by the stamen (Figure S11D). As in other tissues, bill-siRNA clusters were longer than pau-siRNA clusters in the sperm and vegetative cell (Figure S11E). The first base of bill-siRNAs in the sperm and vegetative is enriched for A (Figure S11F).

### 2.6. The Bill-siRNAs of the Gametes Are Absent in Embryo and Seedling

**Figure 5:**
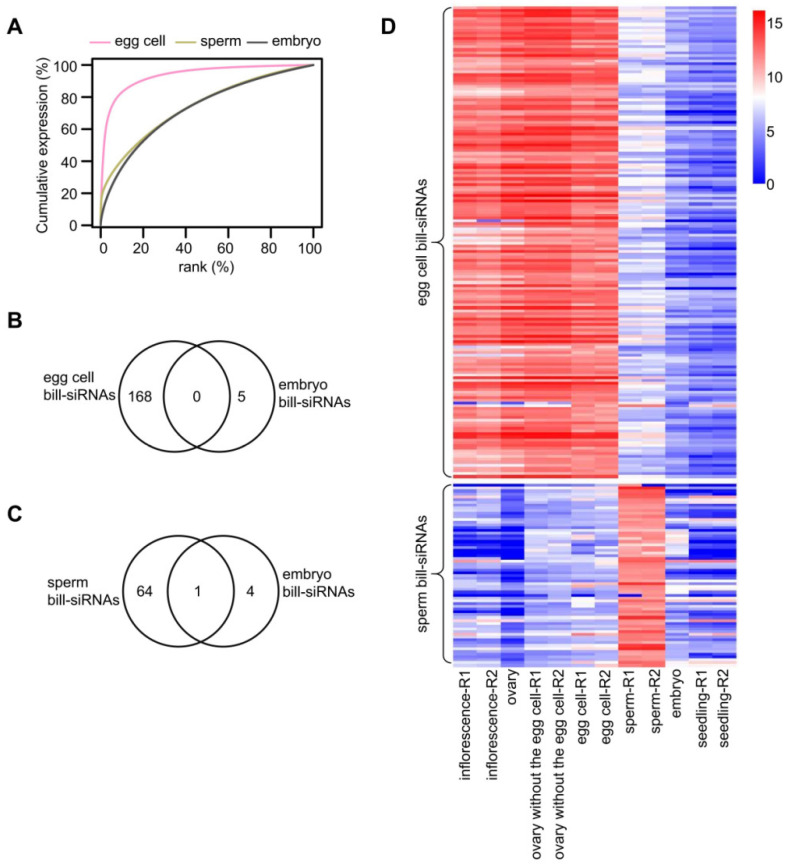
Bill-siRNAs of gametes are absent in embryos. (**A**) Cumulative expression plot of 24 nt siRNA clusters in egg cells, sperm, and embryos. (**B**) Overlap between bill-siRNA clusters in egg cells and embryos. (**C**) Overlap between bill-siRNA clusters in sperm and embryos. (**D**) Heatmaps indicating the siRNA abundance on bill-siRNA clusters in egg cells and sperm.

To track the bill-siRNAs of the gametes after fertilization, we examined sRNA-seq data of 7- to 8-day-old embryos [17]. A cumulative expression plot indicates that the siRNA concentration is substantially different in the embryo than in the egg cell (Figure 5A). Only five bill-siRNA clusters are identified in the embryo (Figure S12). None of the bill-siRNA clusters in the embryo are shared by those of the egg cell (Figure 5B and Figure S12). In addition, only one bill-siRNA cluster in the embryo is overlapped with that of the sperm (Figure 5C and Figure S12). The abundance of bill-siRNAs is substantially lower in the embryo and seedling than in the egg cell and sperm (Figure 5D). These results suggested that the bill-siRNAs from the gametes have disappeared in the embryo and seedling.

### 2.7. The Bill-siRNAs of the Gametes Perish but New Bill-siRNAs Are Produced in the Endosperm

**Figure 6:**
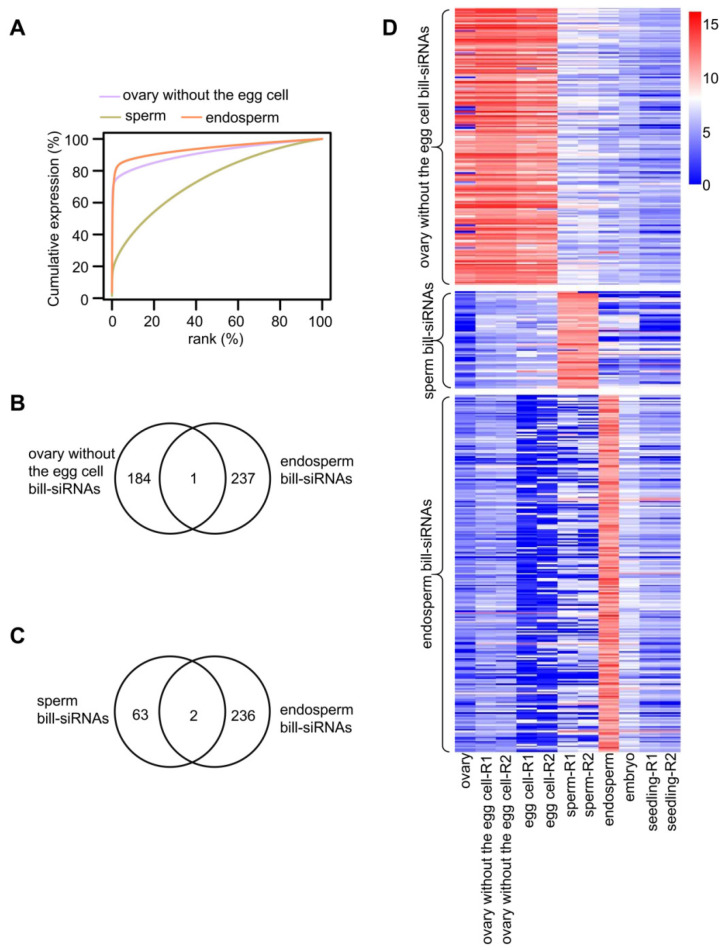
Endosperm produces new bill-siRNAs. (**A**) Cumulative expression plot of 24 nt siRNA clusters in the ovary without the egg cell, sperm, and endosperm. (**B**) Overlap between bill-siRNA clusters of the ovary without the egg cell and endosperm. (**C**) Overlap between bill-siRNA clusters of sperm and endosperm. (**D**) Heatmaps showing the siRNA abundance on bill-siRNA clusters in ovary, sperm, and endosperm.

Given that sRNA-seq data in the central cell were lacking, and that the siRNAs largely overlapped in the ovary, egg cell, and ovary without the egg cell, we used the sRNA data of the ovary without the egg cell as representative of the central cell. A cumulative expression plot shows that siRNA concentration in the endosperm is similar to that in the ovary without the egg cell, which differs from the pattern of siRNA in the sperm (Figure 6A). Among 238 bill-siRNA clusters in the endosperm, only one overlapped with that in the ovary without the egg cell (Figure 6B). On this overlapped siRNA cluster, however, the siRNA distribution was quite different in the endosperm than in the ovary (Figure S13A). In addition, only two bill-siRNA clusters of the endosperm were shared by the sperm (Figure 6C and Figure S13B). A heatmap demonstrated that the bill-siRNAs of the endosperm differed from those of the sperm or of the ovary without the egg cell (Figure 6D). Overall, the bill-siRNAs of the male and female gamete perished in the endosperm, and new bill-siRNAs were produced in the endosperm.

**Figure 7:**
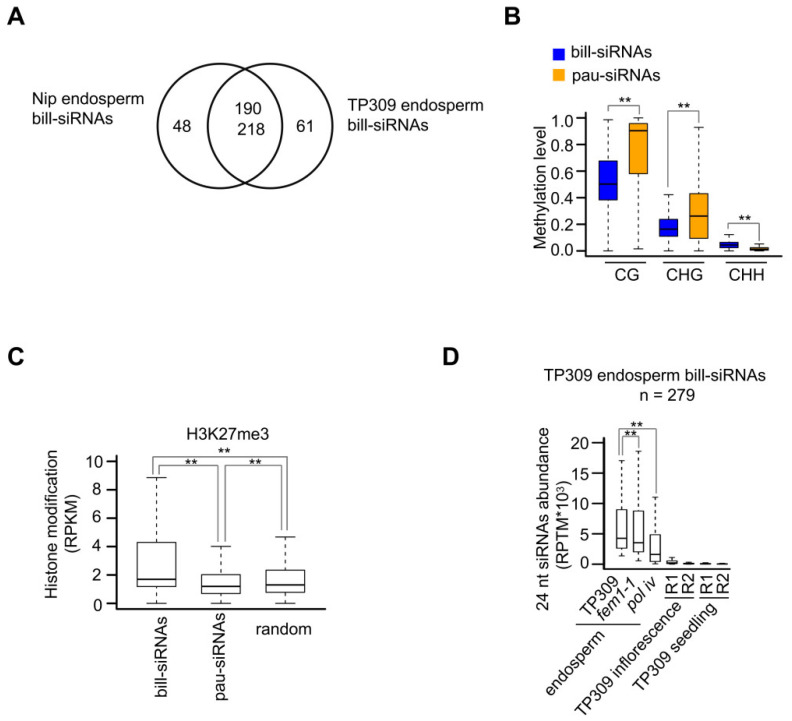
Biosynthesis of bill-siRNAs in the endosperm is dependent on OsRDR2 and Pol IV. (**A**) Overlap between bill-siRNAs in the endosperm of Nipponbare and TP309. (**B**) Boxplots showing the methylation levels in CG, CHG, and CHH on bill-siRNA and pau-siRNA clusters of the endosperm. (**C**) Comparison of H3K27me3 enrichment on bill-siRNA clusters and pau-siRNA clusters in the endosperm. ** indicates *p* < 0.01 by the Wilcoxon sum test. (**D**) Boxplots showing siRNA abundance on bill-siRNA clusters of TP309 endosperm. ** indicates *p* < 0.01 by the Wilcoxon sum test.

siRNAs were also concentrated on a few loci in the endosperm of TP309 (Figure S14A). Among the 279 bill-siRNA clusters in the TP309 endosperm, 218 were shared by Nipponbare (Figure 7A). The bill-siRNA clusters of the TP309 endosperm were significantly longer than the pau-siRNA clusters of the endosperm in both Nipponbare and TP309 (Figure S14B). The first base of the bill-siRNAs in the endosperm in both Nipponbare and TP309 was enriched at A (Figure S14C). The percentage of A was lower in the bill-siRNAs of the endosperm than in those of the ovary (Figures S14C and S7A). The methylation levels of CG and CHG were significantly lower on bill-siRNA clusters than on pau-siRNA clusters (Figure 7B). The methylation levels of CHH, however, were significantly higher on bill-siRNA clusters than on pau-siRNA clusters (Figure 7B). For the repressive epigenetic mark H3K27me3 in the endosperm, the enrichment was significantly higher on bill-siRNA clusters than on pau-siRNA clusters (Figure 7C). The biosynthesis of bill-siRNAs in the endosperm was partially dependent on *FEM1*/*OsRDR2* and Pol IV because the abundance of siRNAs was significantly decreased in *fem1-1* (*osrdr2-1*) and *pol iv* (*osnprd1a-1/osnrpd1b-1*) mutants in the TP309 background (Figure 7D).

### 2.8. Ovary Bill-siRNA Persist in Seed Coat

**Figure 8:**
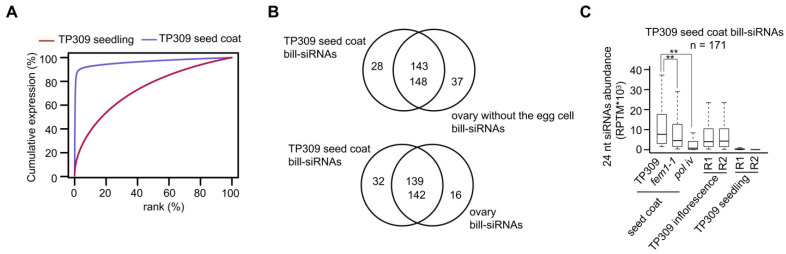
Bill-siRNAs derived from the ovary are retained in the seed coat. (**A**) Cumulative expression plot of 24 nt siRNA clusters in seedlings and seed coats in TP309 background. (**B**) Overlap for bill-siRNA clusters in the seed coat and in the ovary without the egg cell (upper), and in the ovary with egg cell (lower). (**C**) Boxplots showing siRNA abundance in the indicated tissues on TP309 seed coat bill-siRNA clusters. ** indicates *p* < 0.01 by the Wilcoxon sum test.

The seed coat develops from somatic cells in the ovary without the egg cell and central cell. To track the bill-siRNAs of the ovary without the egg cell, we sequenced the sRNA in the seed coat of 8-day-old seeds. A cumulative expression plot indicated that siRNAs were more concentrated in the seed coat than in the seedling (Figure 8A). Of the 171 bill-siRNA clusters in the seed coat, 143 overlapped with those of the ovary without the egg cell, and 139 overlapped with that of the ovary with the egg cell (Figure 8B), suggesting that the majority of the bill-siRNAs in the ovary persist in the seed coat. The bill-siRNA clusters in the seed coat were significantly longer than the pau-siRNA clusters in the seed coat (Figure S15A). The first base of the bill-siRNAs in the seed coat was also enriched at A (Figure S15B). On the bill-siRNA clusters in the seed coat, siRNA abundance was substantially reduced in the *fem1-1* (*osrdr2-1*) and *pol iv* (*osnrpd1a-1*/*osnrpd1b-1*) mutants (Figure 8C), indicating that the biosynthesis of bill-siRNAs in the seed coat is largely dependent on *FEM1*/*OsRDR2* and Pol IV.

## 3. Discussion

We identified highly expressed bill-siRNAs in the rice inflorescence, which mainly originated from the ovary, i.e., the female reproductive organ. We noted that other parts of the inflorescence such as the bract, the branch, and the stamen also generated bill-siRNAs, but at lower levels than the ovary. As was the case for pau-siRNAs, the first base of bill-siRNAs was enriched at adenine, suggesting that bill-siRNAs are likely loaded into OsAGO4. Although they are less efficient than pau-siRNAs, bill-siRNAs are necessary for DNA methylation. Moreover, the biosynthesis of both bill-siRNAs and pau-siRNAs is dependent on *FEM1*/*OsRDR2*. Bill-siRNAs are much more abundant than pau-siRNAs, but based on the biosynthesis and mode of action, both bill-siRNAs and pau-siRNAs are 24 nt siRNAs. The bill-siRNA clusters are generally longer than the pau-siRNA clusters. The repressive and active epigenetic marks on bill-siRNA clusters are higher and lower than on pau-siRNA clusters, respectively. As a result, the transcript levels of bill-siRNA cluster-adjacent genes are lower than those of pau-siRNA cluster-adjacent genes. However, the bill-siRNAs seem to have little effect in regulating gene expression of bill-siRNA cluster-adjacent genes among different organs and between WT and *osrdr2* mutants. Additional research is needed to determine how bill-siRNAs and pau-siRNAs differ in function. Additional research is also needed to identify the mechanism underlying the high expression of bill-siRNAs in the ovary.

By examining the whole life history of rice from seedling to seed development, we found that bill-siRNAs are much more abundant in reproductive organs, especially in the ovary, than in vegetative tissues. The bill-siRNAs in the ovary, the egg cell, and the ovary without the egg cell mostly overlapped, indicating that the entire ovary may produce bill-siRNAs. Given that reproductive defects occurred in *osrdr2* mutants [11], the bill-siRNAs in gametes may have roles in gamete development. Interestingly, bill-siRNAs in gametes disappeared in the embryo and endosperm, but bill-siRNAs were produced on different genomic loci in the endosperm. By contrast, the bill-siRNAs persist in seed coat. Taken together, our results indicate that bill-siRNAs in female gametes and male gametes disappear after double fertilization. The histone modification of H3K27me3 is enriched in the bill-siRNAs in the endosperm, indicating that these bill-siRNAs may have important roles in seed development.

A recent report identified highly expressed siRNAs, i.e., siren siRNAs, in the ovary and seed coat of *Brassica rapa* [12]. Because *B. rapa* is a dicotyledonous plant, its endosperm is temporary and is absorbed by the cotyledon during seed development. In monocotyledonous plants, however, the endosperm stores starch and other nutrients required for embryo development and seed germination. Our results indicate that bill-siRNAs are expressed at lower levels in the embryo and endosperm than in the ovary or seed coat, but new bill-siRNAs are produced in the endosperm. The current results and those of Grover et al. [12] indicated that bill-siRNAs might differ between monocotyledonous and dicotyledonous plants.

Although bill-siRNAs may have little effect on gene expression of the bill-siRNA cluster-adjacent genes, our results indicate that they have a significant role in DNA methylation and that they may have important roles in tissue development. According to recent research, highly expressed siRNAs in ovary [18] and tapetum cells [19] might function in directing DNA methylation in trans. The mechanisms and functions of bill-siRNAs warrant further research.

## 4. Materials and Methods

### 4.1. Plant Materials and Growth Conditions

Rice varieties included Taipei 309 (TP309) (*Oryza sativa*, japonica) and Nipponbare (*Oryza sativa*, japonica). The *osrdr2-3* and *osrdr2-6* mutants were in the background of Nipponbare [11]. The *fem1-1* (*osrdr2-1*) and *pol iv* (*osnrpd1a-1*/*osnrpd1b-1*) mutants were in the background of TP309 [11,20].

For the isolation of RNAs from the reproductive organs of Nipponbare, *osrdr2-3*, and *osrdr2-6*, the inflorescences, ovaries, stamens, bracts, and branches were obtained at stage 12 for stamen development from rice plants that were grown in a paddy field in Nanjing in 2019. For the isolation of RNAs from the reproductive organs of TP309, *fem1-1* (*osrdr2-1*), and *pol iv* (*osnrpd1a-1*/*osnrpd1b-1*), the inflorescences (at stage 12 for stamen development), endosperms (8 days after pollination), and seed coats (8 days after pollination) were obtained from rice plants that were grown in a paddy field in Nanjing in 2020. Rice seedlings were grown in Kimura B nutrient solution in a growth chamber (16 h/8 h light/dark with a 28 °C/25 °C cycle).

### 4.2. Whole-Genome Bisulfite Sequencing Analysis

The analysis of whole-genome bisulfite sequencing was performed as previously reported [11]. In brief, the low-quality reads were discarded by NGSQCToolkit. The clean reads were aligned to release 7 of the rice reference genome [21] by bismark (-N 1). After PCR duplicates were removed by deduplicate_bismark, the bismark_methylation_extractor was used to call methylation. The methylation levels were calculated through dividing the total number of methylated cytosines by the total number of sequenced cytosines in a region.

### 4.3. mRNA Sequencing and Analysis

About 1 μg of total RNA was used to enrich mRNA with oligo dT by magnetic beads. The mRNA was broken into short fragments and was used as template to synthesize cDNA. The purified double-stranded DNA was end-repaired, added with A-tailing, and ligated with adapters. PCR with 10–17 cycles was performed to amplify the library. The qualified library was sequenced on the Hiseq X10 platform (Annoroad Gene Technology Co., Ltd., Beijing, China).

The clean reads were aligned to release 7 of the rice reference genome [21] by Tophat (-m 1). The expression level for each gene was calculated using the FRKM (fragments per kilobase of exon per million mapped reads) method. The DEGs were identified with the cutoff of the fold change > 2 and the FDR < 0.05.

### 4.4. Small RNA Sequencing and Analysis

A 1 μg quantity of total RNA was used to separate sRNAs with lengths of 18 to 30 nt. The purified sRNAs were ligated with adapters and reverse-transcribed into cDNA. The cDNA fragments were amplified by 15–18 cycles of PCR. The PCR products ranging from 110 to 130 bp were isolated and purified. The purified DNA was heat-denatured and circularized. The qualified library was sequenced on the BGISEQ-500 platform.

The low-quality sRNA reads were removed. The clean sRNA reads were aligned to release 7 of the rice reference genome [21] by BOWTIE with 0 mismatch (-v 0). Reads that mapped to structural RNAs (rRNA, tRNA, snRNA, and snoRNA) were discarded. The uniquely mapped 24 nt reads were retained for subsequent analysis. The read counts were normalized by the reads per ten million mapped reads (RPTM) according to the total number of mapped reads. The scripts were uploaded to Github (https://github.com/Lily6927/sRNA-analysis.git) (accessed on 8 July 2022).

To define 24 nt siRNA clusters, the unique sRNA reads were merged (replicates were combined) less than 100 bp. Read counts in merged regions were calculated by bedtools and normalized with RPTM. Regions with more than 12 RPTM were considered 24 nt siRNA clusters. The siRNA clusters with RPTM > 2000 were identified as billionaire siRNA clusters (bill-siRNA clusters). The other siRNA clusters were identified as pauper siRNA clusters (pau-siRNA clusters).

### 4.5. ChIP-seq Analysis

After the low-quality reads were filtered by NGSQCToolkit (version v2.3), clean reads were aligned to release 7 of the rice reference genome [21] using Bowtie2 (version v2.3.2). The relative enrichment of histone modifications was normalized by RPKM.

### 4.6. Small RNA Northern Blotting

Total RNAs were extracted from 18-day-old seedlings, inflorescences, stamens, and ovaries using TRIzol (Invitrogen Corporation, Carlsbad, CA, USA, 15596018). The isolated RNAs were added with an equal volume of PEG8000 (20% PEG8000, 1 M NaCl), and were centrifuged at 13,000 rpm at 4 °C. The supernatant was combined with a 0.1 volume of 3 M NaAC and was centrifuged at 13,000 rpm at 4 °C. The pellet was dissolved in DEPC-treated water to obtain small RNAs. About 20 μg of denatured sRNAs were subjected overnight to 15% denaturing PAGE gel electrophoresis. The sRNA was transferred to a Hybond-N^+^ membrane (General Electric Company, Fairfield, CT, USA, RPN303B) with 400 mA for 3 h. The cross-linked membrane was hybridized with the ^32^P-labeled probe at 38 °C overnight. The signal was detected with a Multifunctional Molecular Imager (General Electric Company, Fairfield, CT, USA, Typhoon FLA9500). The oligos used for the synthesizing probe are listed in Table S3.

### 4.7. Chop-PCR

A 1 μg quantity of genomic DNA was digested by *Hae*III (NEB, R0108S) in a 30 μL reaction mixture at 37 °C for 16 h. A 2 μL volume of the digested DNA was used as a template to perform PCR in a 20 μL reaction mixture. The oligos used for Chop-PCR are listed in Table S3.

## Data Availability

Raw data of sRNA sequencing (bracts and branches of Nipponbare; endosperms and seed coats of TP309, *fem1-1*, and *pol iv* mutants) and mRNA sequencing (seedlings and inflorescences of TP309) generated in this study have been deposited in the NCBI Gene Expression Omnibus (GEO) (GSE154501; Table S2). The whole-genome bisulfite sequencing data of seedlings, inflorescences, stamens, and ovaries were deposited in the NCBI GEO in our previous report (GSE112259, GSE130168, and GSE152155; [11]; Table S2). The sRNA sequencing data of seedlings, inflorescences, stamens, and ovaries were deposited in the NCBI GEO in our previous report (GSE112259, GSE130168, and GSE152155; [11]; Table S2). The mRNA sequencing data of seedlings, inflorescences, stamens, and ovaries were also previously reported (GSE112259, GSE130168, and GSE152155; [11]; Table S2). The whole-genome bisulfite sequencing data of endosperm were downloaded from NCBI GEO (GSE117187; [22]; Table S2). The sRNA sequencing data of egg cells, ovaries without the egg cells, sperms, and vegetative cells were downloaded from NCBI (PRJNA533115; [16]; Table S2). The sRNA sequencing data of embryos and endosperms were from NCBI GEO (GSE44898; [17]; Table S2). The ChIP-seq data of H3K4me1, H3K4me3, H3K9me2, H3K27me3, H3K27ac, and Pol II in the Nipponbare inflorescence were downloaded from NCBI GEO (GSE142570; [14]; Table S2). The ChIP-seq data of H3K27me3 in Nipponbare endosperms were downloaded from NCBI GEO (GSE27048; [23]; Table S2).

## Supplementary Materials

The following supporting information can be downloaded at: https://www.mdpi.com/article/10.3390/plants11151957/s1, Figure S1: Abundance of 24 nt siRNAs was lower in inflorescences than in seedlings; Figure S2: Bill-siRNAs in TP309; Figure S3: Bill-siRNAs of inflorescences are conserved in rice varieties; Figure S4: Bill-siRNAs in inflorescences have little effect on gene transcription of adjacent genes; Figure S5: Genomic distribution of 24 nt siRNAs in different tissues; Figure S6: Biosynthesis of bill-siRNAs is dependent on OsRDR2 in ovaries and stamens; Figure S7: Characteristics of bill-siRNAs in stamens and ovaries; Figure S8: Bill-siRNAs in egg cells and in ovaries without the egg cells; Figure S9: Bill-siRNAs in egg cells and ovaries without the egg cell largely overlapped; Figure S10: The features of bill-siRNAs of egg cell and ovary without the egg cell; Figure S11: Bill-siRNAs in sperm and vegetative cells; Figure S12: Bill-siRNAs in embryos; Figure S13: Overlap of bill-siRNAs in endosperm vs. gametes; Figure S14: Features of bill-siRNAs in endosperms; Figure S15: Features of bill-siRNAs in seed coats; Table S1: Bill-siRNA clusters list; Table S2: Basic information of high-throughput sequencing; Table S3: Primers list.
