# Supplementary material for: The Rise and Fall of Billionaire siRNAs during Reproductive Development in Rice
Source: Plants (Basel). 2022 Jul 28;11(15):1957. doi: 10.3390/plants11151957 (PMC9370656; doi:10.3390/plants11151957)
Supplement: Supplementary file 1 [file plants-11-01957-s001.zip › plants-1800426-Figures.pdf]

Figure S1

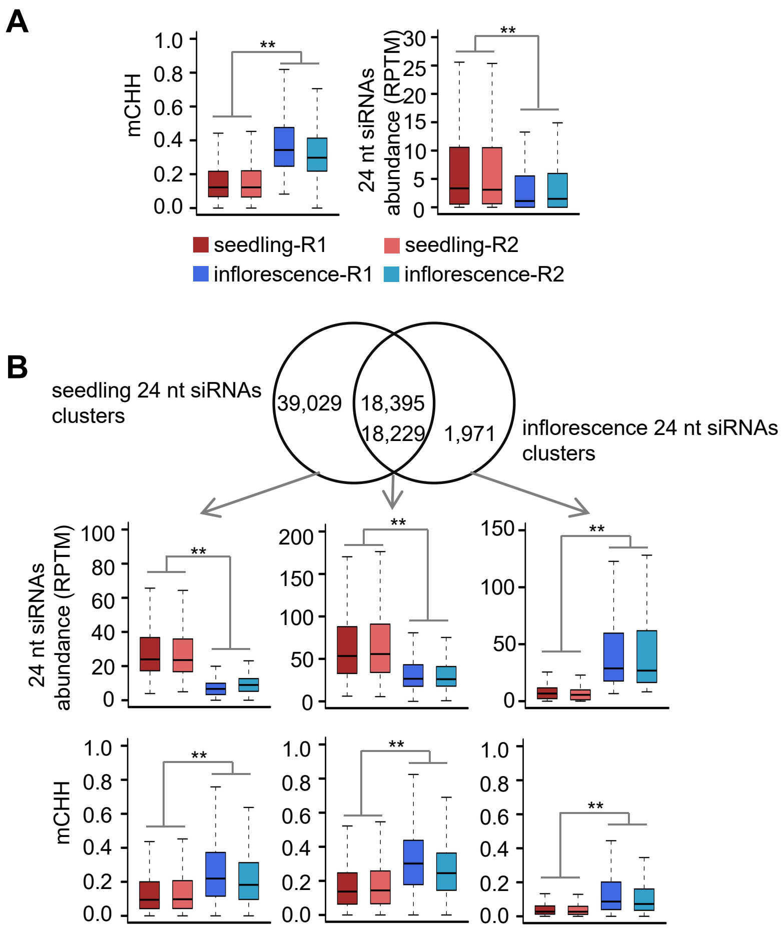

Figure S1. Abundance of 24 nt siRNAs was lower in inflorescences than in seedlings.
A. Boxplots showing the CHH methylation levels and 24 nt siRNA abundance in WT seedlings and WT inflorescences on CHH hyper-DMRs of inflorescences relative to seedlings. ** indicate $p < 0.01$ by the Wilcoxon sum test. **B.** Venn diagram indicating the overlap between seedling 24 nt siRNA clusters and inflorescence 24 nt siRNA clusters. Boxplots indicate the 24 nt siRNA abundance and CHH methylation level on common and specific regions. ** indicates $p < 0.01$ by the Wilcoxon sum test.

Figure S2

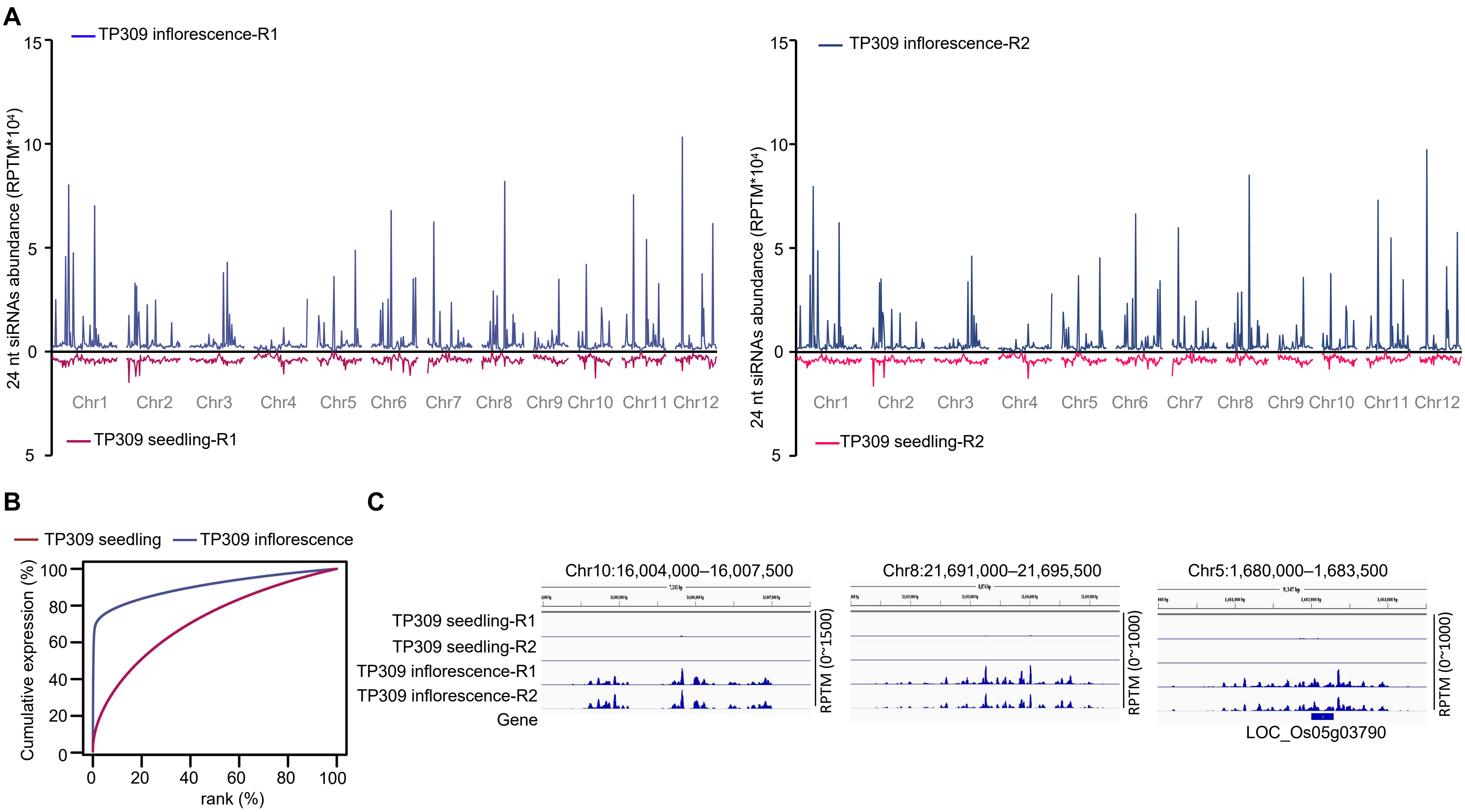

Figure S2. Bill-siRNAs in TP309.
A. Comparison of 24 nt siRNA distributions between inflorescences and seedlings in TP309. Y-axis indicates the siRNA abundance by RPTM within 500-kb windows. **B.** Cumulative expression plot of 24 nt siRNAs in TP309 inflorescences and seedlings. The expression of each cluster was indicated by RPTM using two replicates, was ranked, and their cumulative expression was plotted. siRNA clusters were analyzed if the RPTM ≥ 12 (n = 23,635 in inflorescences, n = 46,743 in seedlings). **C.** Screen shots of IGV showing 24 nt siRNAs levels in TP309 inflorescences and seedlings on three representative bill-siRNA clusters.

Figure S3

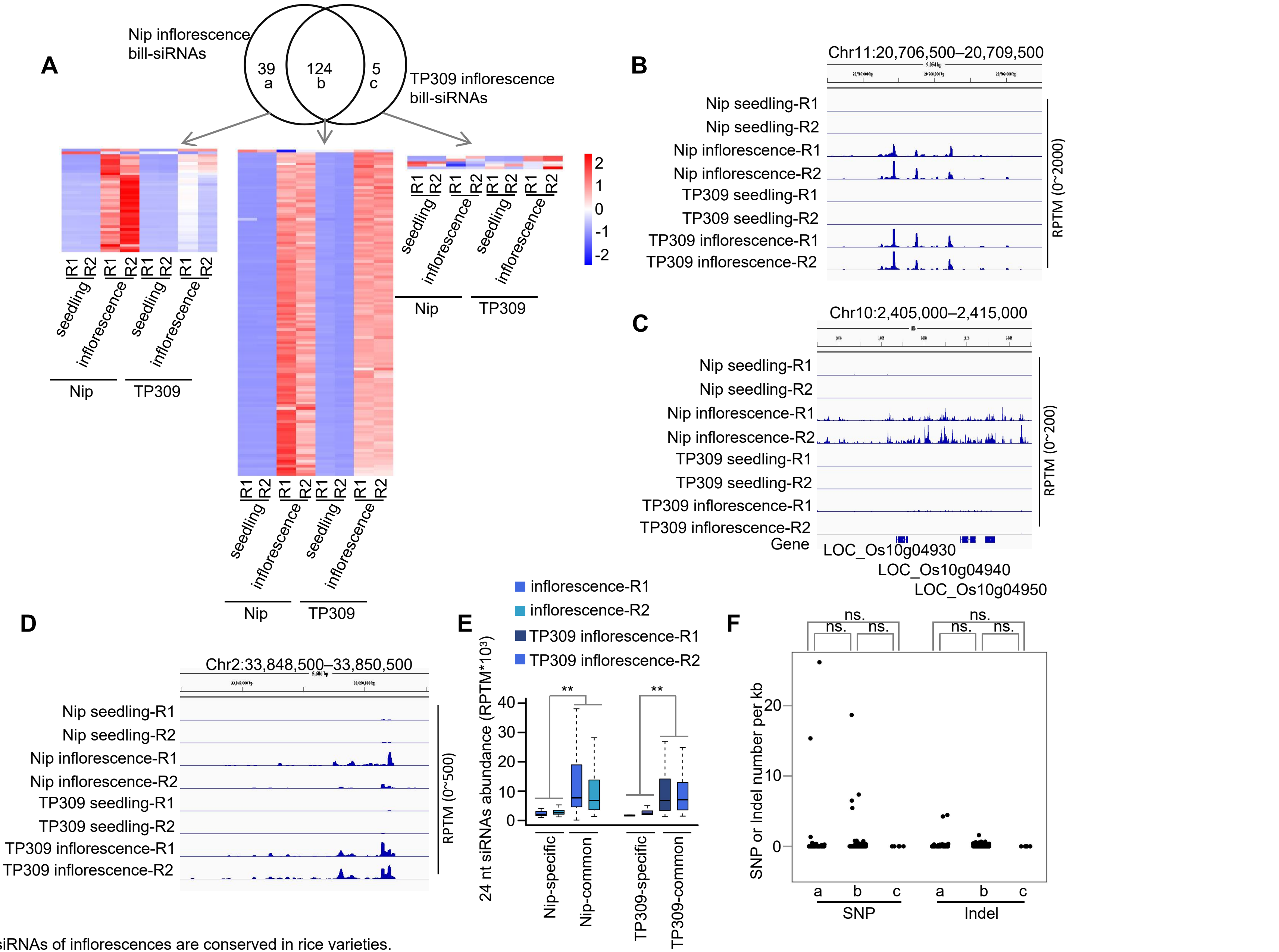

Figure S3. Bill-siRNAs of inflorescences are conserved in rice varieties.

A. Overlap between bill-siRNAs of Nipponbare and TP309 inflorescences. Heatmaps indicate the relative abundance of siRNAs on common or variety-specific bill-siRNA clusters. **B.** Screen shot of IGV indicating siRNA abundance on one representative common bill-siRNA cluster. **C.** Screen shot of IGV indicating siRNA abundance on one representative Nipponbare-specific bill-siRNA cluster. **D.** Screen shot of IGV indicating siRNA abundance on one representative TP309-specific bill-siRNA cluster. **E.** Boxplots indicating siRNA abundance on common and specific bill-siRNA clusters. ** indicates $p < 0.01$ by the Wilcoxon sum test. **F.** Strip-plots showing the frequency of SNP and Indel in Nipponbare and TP309. ns indicates non-significance ($p > 0.05$) by the Wilcoxon sum test.

Figure S4

A

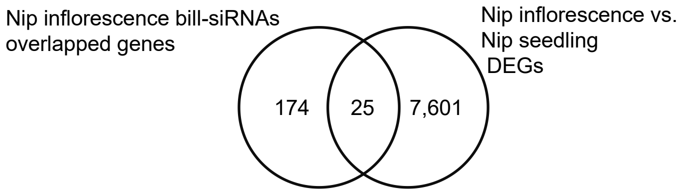

B

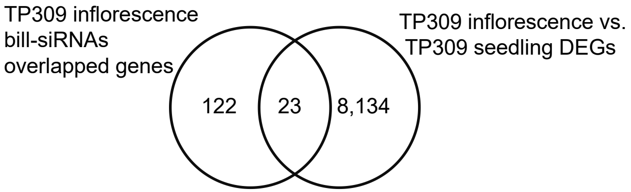

Figure S4. Bill-siRNAs in inflorescences have little effect on transcriptional levels of adjacent genes.
A. Venn diagram indicating the overlap between bill-siRNA cluster-adjacent genes in inflorescence and DEGs of inflorescences vs. seedlings in Nipponbare. **B.** Venn diagram indicating the overlap between bill-siRNA cluster-adjacent genes in inflorescence and DEGs of inflorescences vs. seedlings in TP309.

Figure S5

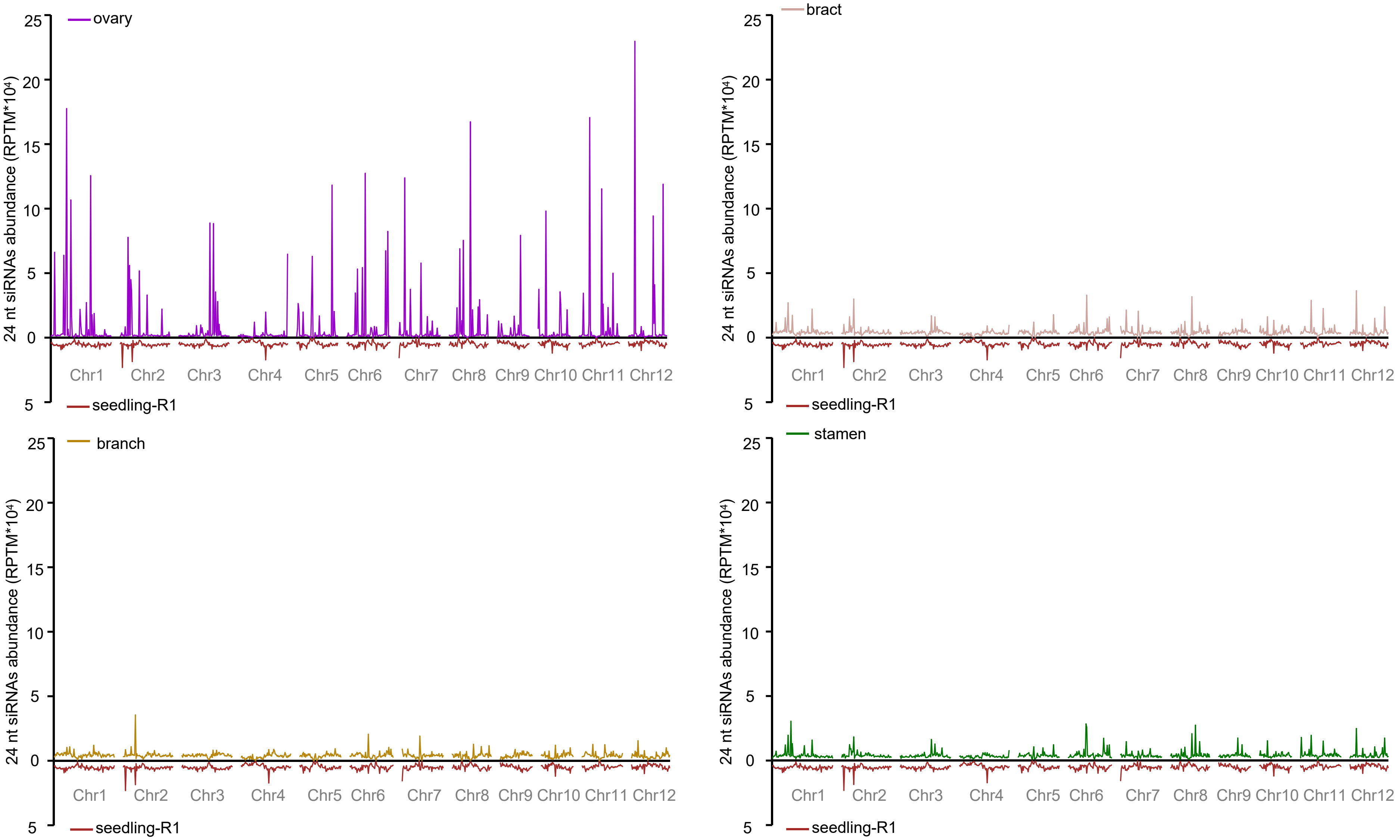

Figure S5. Genomic distribution of 24 nt siRNAs in different tissues.
Comparison of 24 nt siRNA distributions between four tissues and seedlings in Nipponbare. Y-axis indicates the siRNA abundance by RPTM within 500-kb windows.

Figure S6

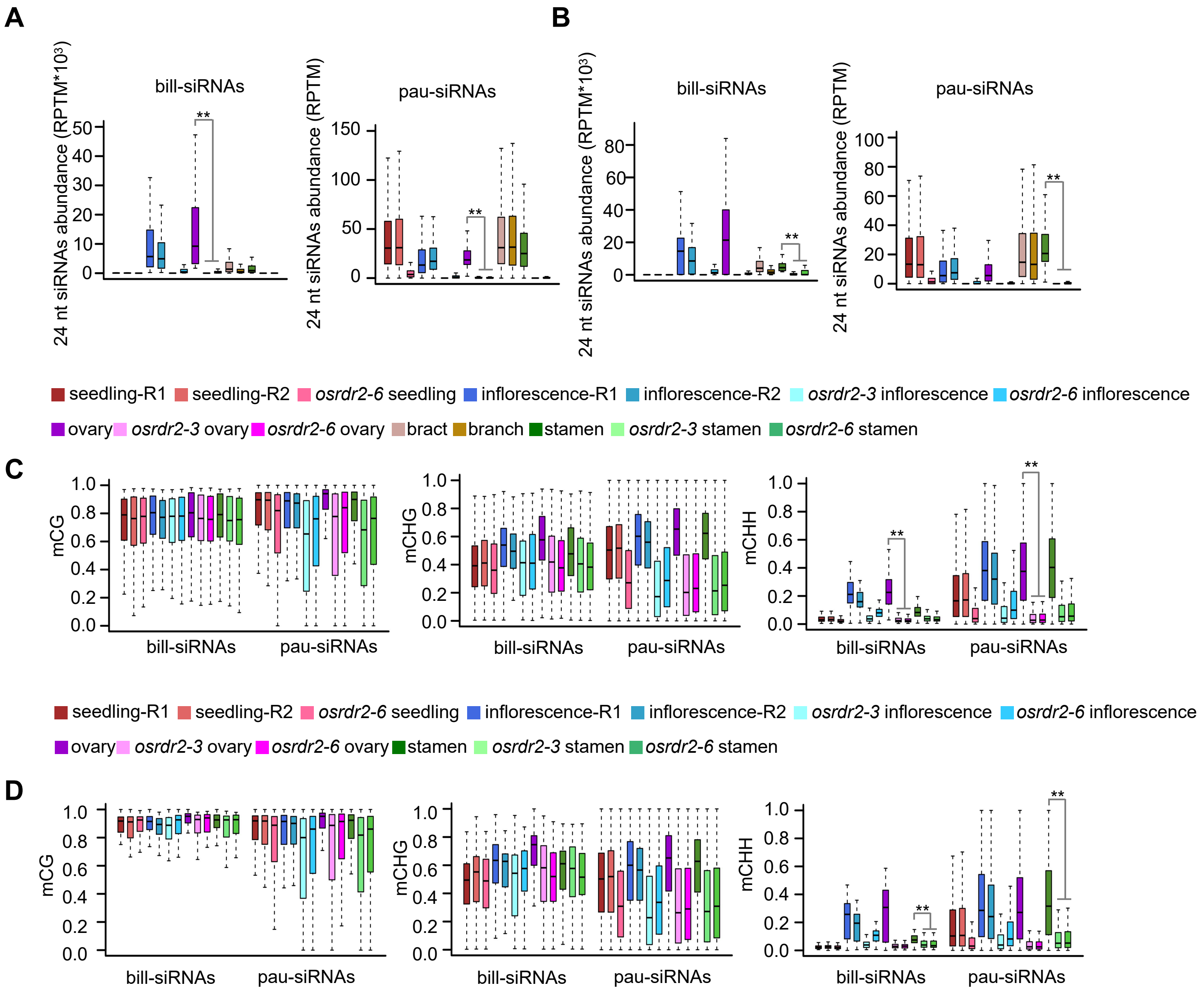

Figure S6. Biosynthesis of bill-siRNAs is dependent on OsRDR2 in ovaries and stamens.

A. Boxplots indicating the 24 nt siRNA levels in different tissues of various genotypes on ovary siRNA clusters. ** indicates $p < 0.01$ by the Wilcoxon sum test. **B.** Boxplots indicating the 24 nt siRNA levels in different tissues of various genotypes on stamen siRNA clusters. ** indicates $p < 0.01$ by the Wilcoxon sum test. **C.** Boxplots indicating the DNA methylation level of CG, CHG, and CHH in different tissues of various genotypes on ovary siRNA clusters. ** indicates $p < 0.01$ by the Wilcoxon sum test. **D.** Boxplots indicating the 24 nt siRNA levels in different tissues of various genotypes on stamen siRNA clusters. ** and * indicated $p < 0.01$ and < 0.05, respectively, by the Wilcoxon sum test.

Figure S7

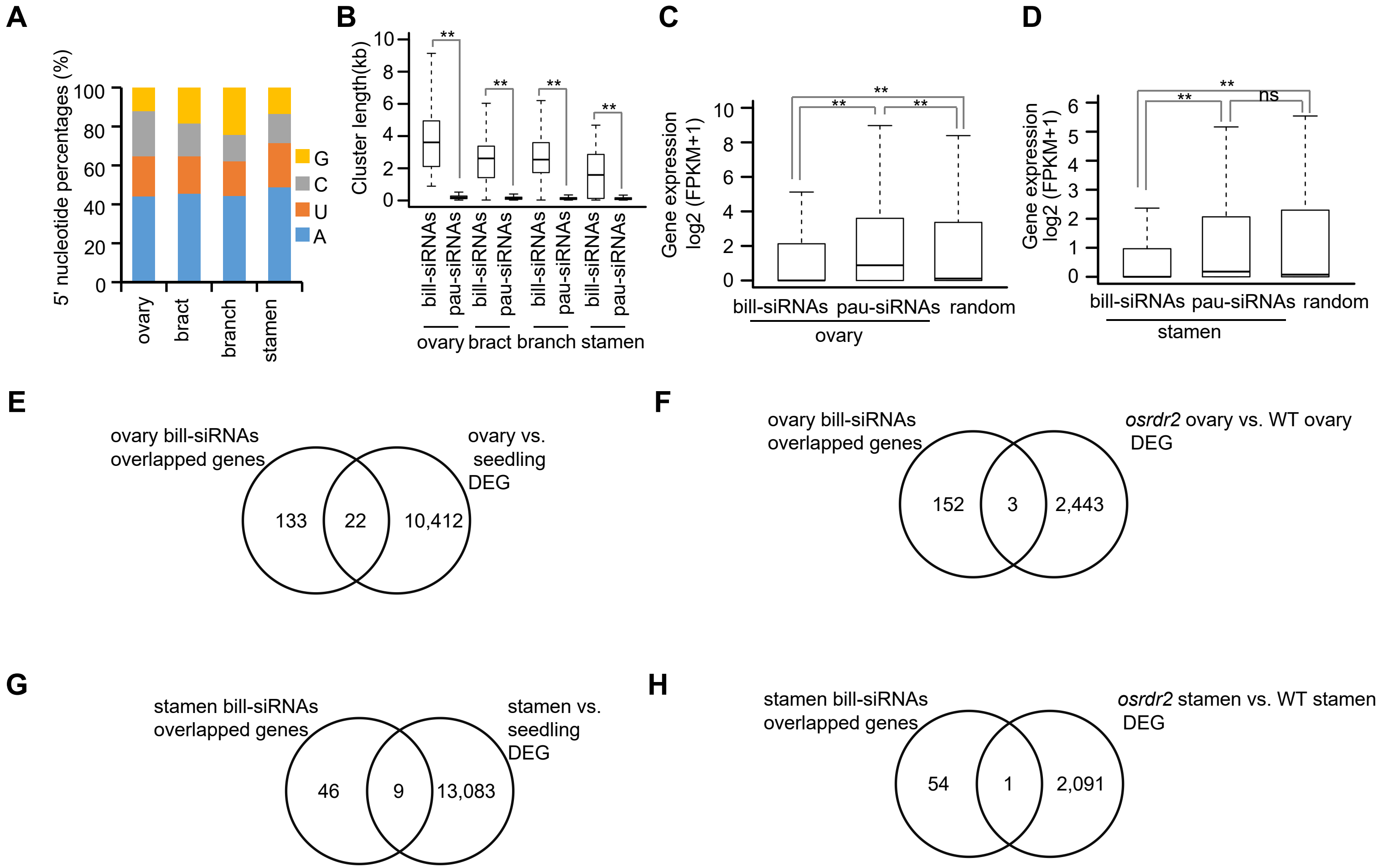

Figure S7. Characteristics of bill-siRNAs in stamens and ovaries.
A. Percentage of four bases on the first nucleotide acid of bill-siRNAs in ovaries, bracts, branches, and stamens. **B.** The lengths of bill-siRNA clusters and pau-siRNA clusters in ovaries, bracts, branches, and stamens. ** indicates $p < 0.01$ by the Wilcoxon sum test. **C.** Relative expression levels of bill-siRNA cluster-adjacent genes, pau-siRNA cluster-adjacent genes, and random genes in ovaries. ** indicates $p < 0.01$ by the Wilcoxon sum test. **D.** Relative expression levels of bill-siRNA cluster-adjacent genes, pau-siRNA cluster-adjacent genes, and random genes in stamens. ** indicates $p < 0.01$, ns indicates no significance ($p > 0.05$) by the Wilcoxon sum test. **E.** Venn diagram indicating the overlap between bill-siRNA cluster-adjacent genes in the ovary and DEGs of ovaries relative to seedlings. **F.** Venn diagram indicating the overlap between bill-siRNA cluster-adjacent genes and DEGs of *osrdr2* relative to WT in the ovary. **G.** Venn diagram indicating the overlap between bill-siRNA cluster-adjacent genes in the stamen and DEGs of stamens relative to seedlings. **H.** Venn diagram indicating the overlap between bill-siRNA clusters-adjacent genes and DEGs of *osrdr2* relative to WT in stamens.

Figure S8

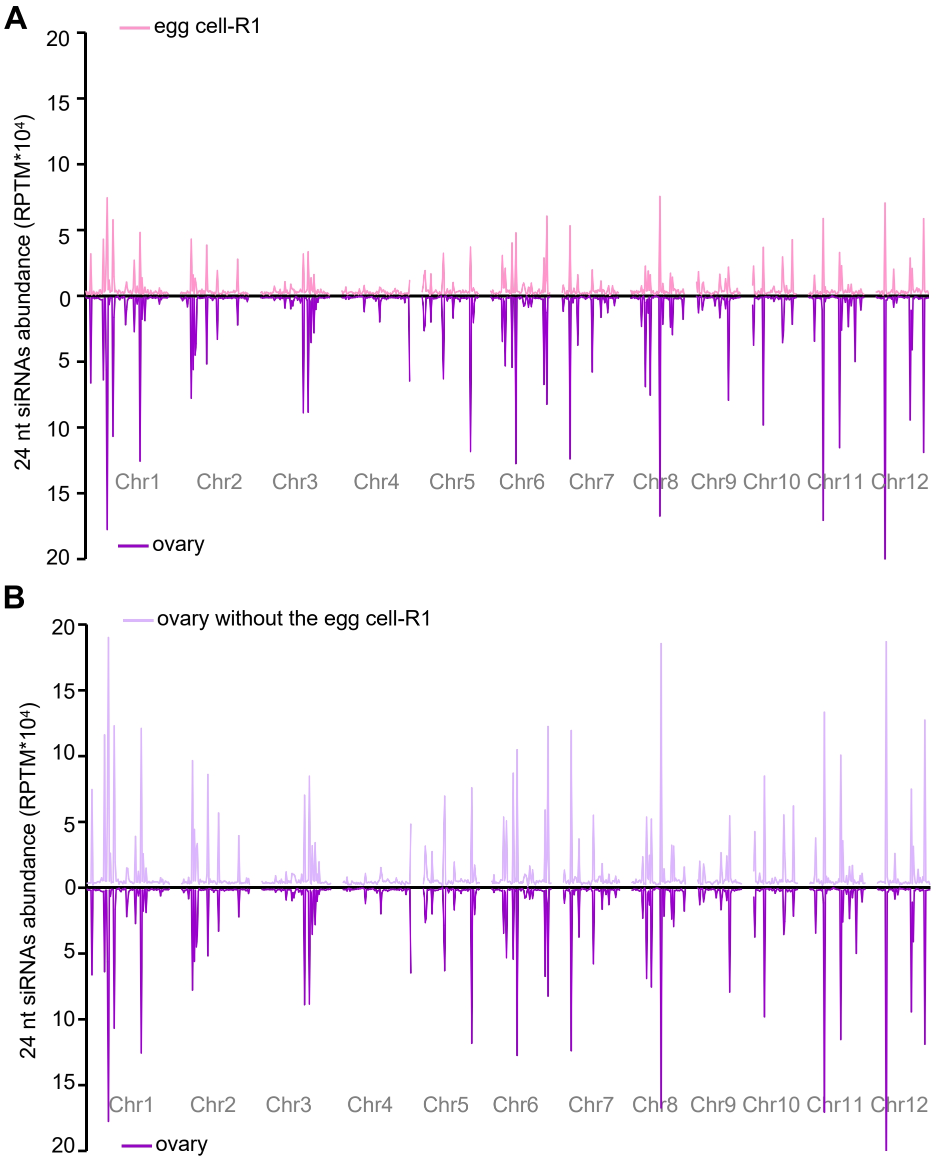

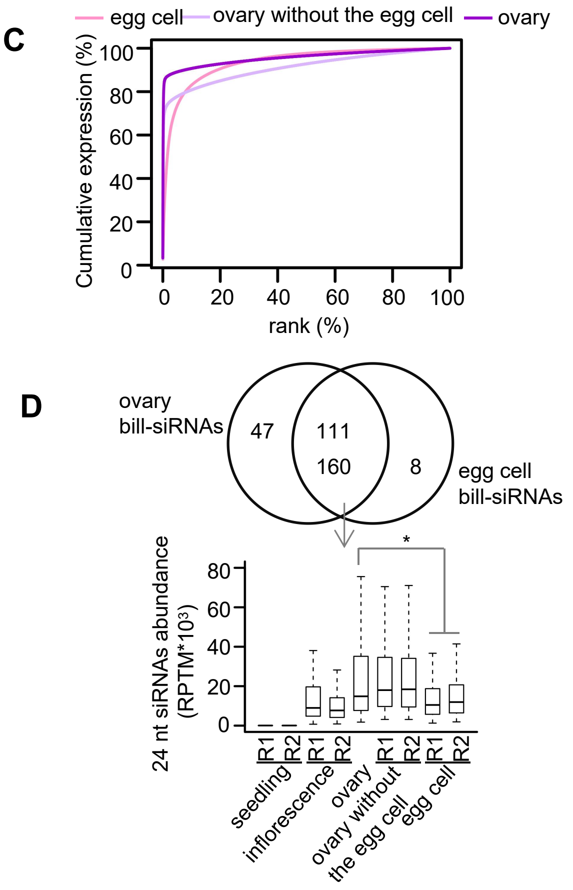

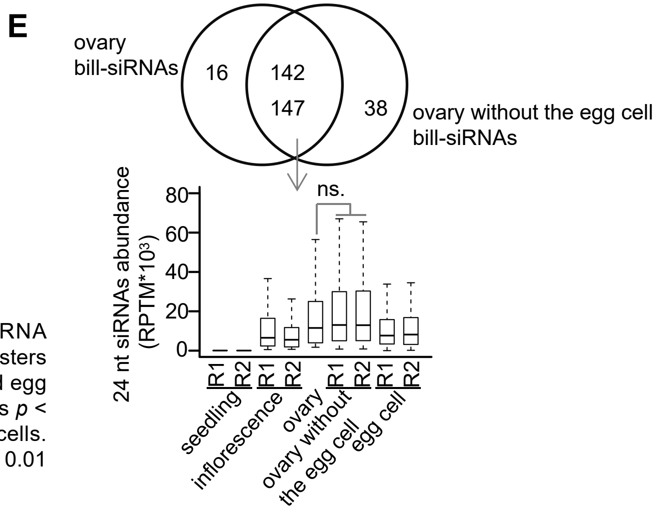

Figure S8. Bill-siRNAs in egg cells and in ovaries without the egg cells.
A. Comparison of 24 nt siRNA distributions between egg cells and ovaries. **B.** Comparison of 24 nt siRNA distributions between ovaries with and without the egg cells. **C.** Cumulative expression plot of 24 nt siRNAs clusters in ovaries, egg cells, and ovaries without the egg cells. **D.** Overlap between bill-siRNA clusters in ovaries and egg cells. Boxplots showing 24 nt siRNA abundance in various tissues on common bill-siRNA clusters. ** indicates $p < 0.01$ by the Wilcoxon sum test. **E.** Overlap between bill-siRNA clusters in ovaries and ovary without the egg cells. Boxplots showing the 24 nt siRNA abundance in various tissues on common bill-siRNA clusters. ** indicates $p < 0.01$ by the Wilcoxon sum test.

Figure S9

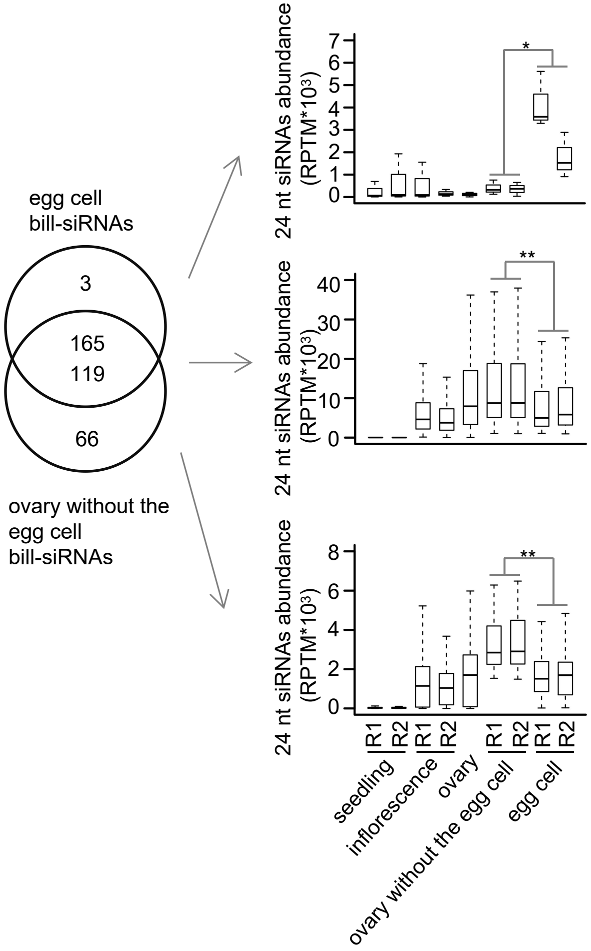

Figure S9. Bill-siRNAs in egg cells and ovaries without the egg cell largely overlapped.
Overlap between bill-siRNA clusters in egg cells and ovaries without the egg cells. Boxplots indicating 24 nt siRNA abundance in the indicated tissues. ** and * indicate $p < 0.01$ and < 0.05, respectively, by the Wilcoxon sum test.

Figure S10

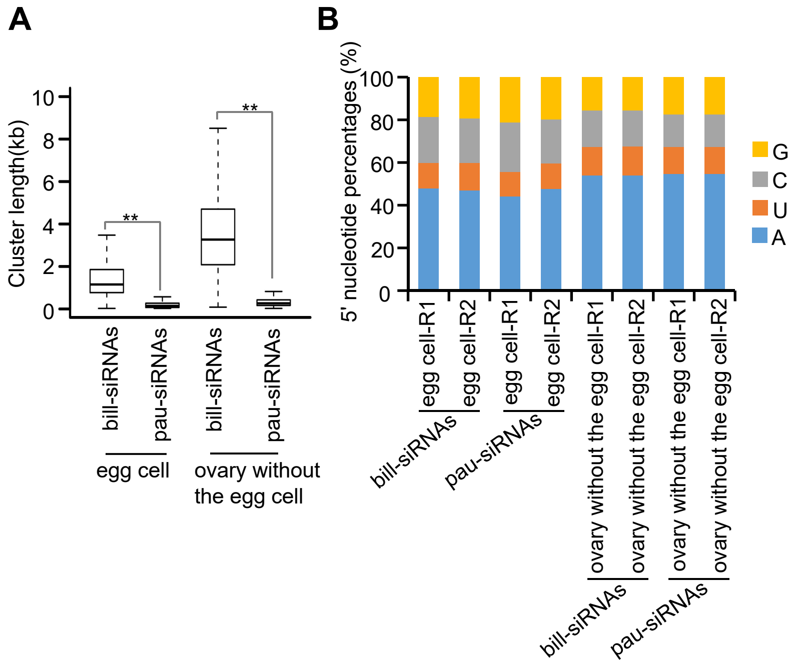

Figure S10. The features of bill-siRNAs of egg cell and ovary without the egg cell.
A. Comparison of the lengths of bill-siRNA clusters and pau-siRNA clusters in egg cells and ovaries without the egg cells. ** indicates $p < 0.01$ by the Wilcoxon sum test. **B.** Percentage of four bases at the first nucleotide acid of bill-siRNAs and pau-siRNAs in egg cells and ovaries without the egg cells.

Figure S11

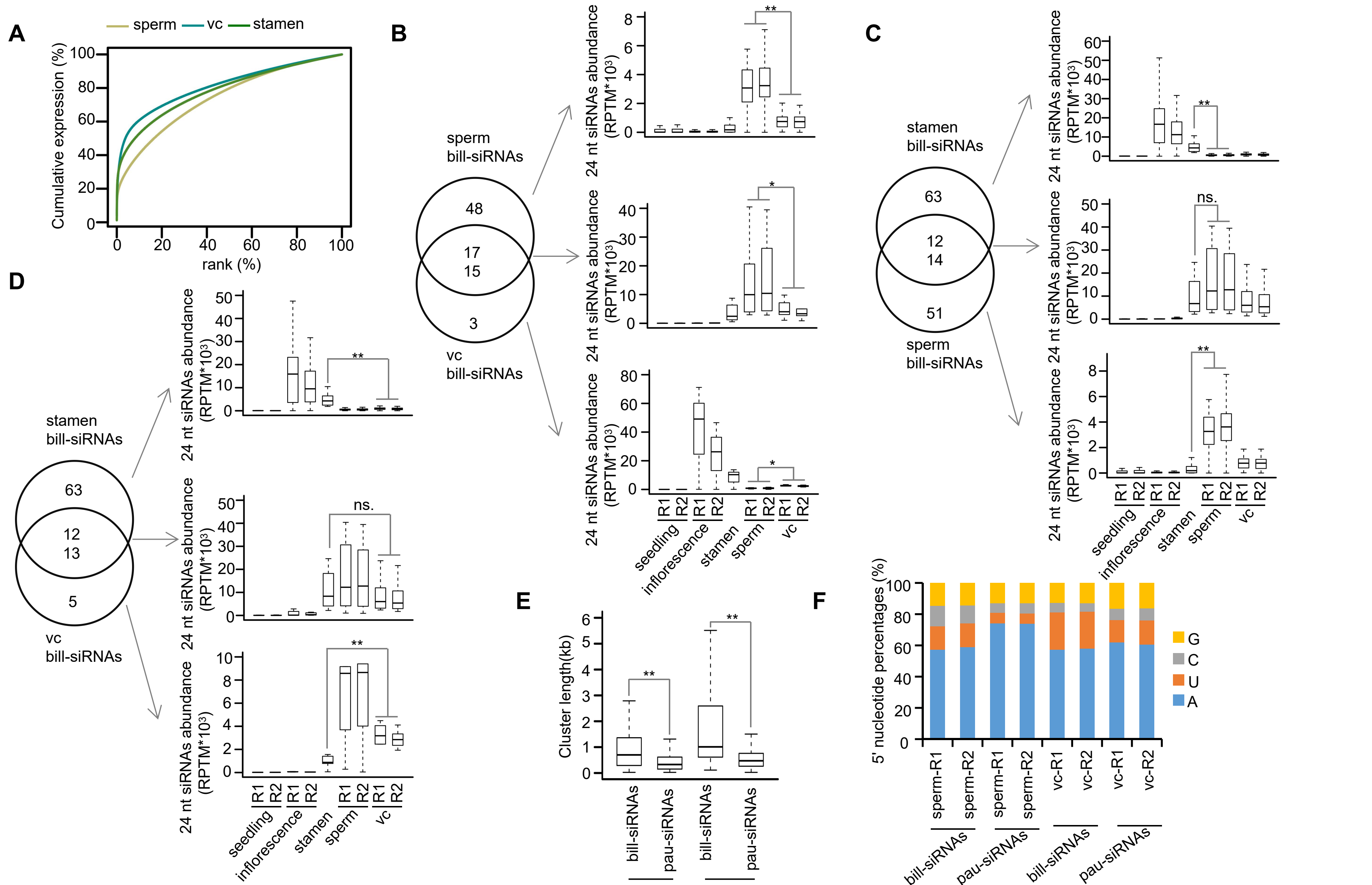

Figure S11. Bill-siRNAs in sperm and vegetative cells.

A. Cumulative expression plot of 24 nt siRNAs in stamens, sperm, and vegetative cells (vc). **B.** Overlap between bill-siRNA clusters in sperm and vegetative cells. Boxplots indicating siRNA abundance in various tissues. ** and * indicate $p < 0.01$ and < 0.05, respectively, and ns indicates no significance by the Wilcoxon sum test. **C.** Overlap between bill-siRNA clusters in stamens and sperm. Boxplots indicating siRNA abundance in various tissues. ** indicates $p < 0.01$, and ns indicates no significance by the Wilcoxon sum test. **D.** Overlap between bill-siRNA clusters in stamens and vegetative cells. Boxplots indicating siRNA abundance in various tissues. ** indicates $p < 0.01$, and ns indicates no significance by the Wilcoxon sum test. **E.** Comparison of the lengths of bill-siRNA clusters and pau-siRNA clusters in sperm and vegetative cells. ** indicates $p < 0.01$ by the Wilcoxon sum test. **F.** Percentage of four bases at the first nucleotide acid of bill-siRNAs and pau-siRNAs in sperm and vegetative cells.

Figure S12

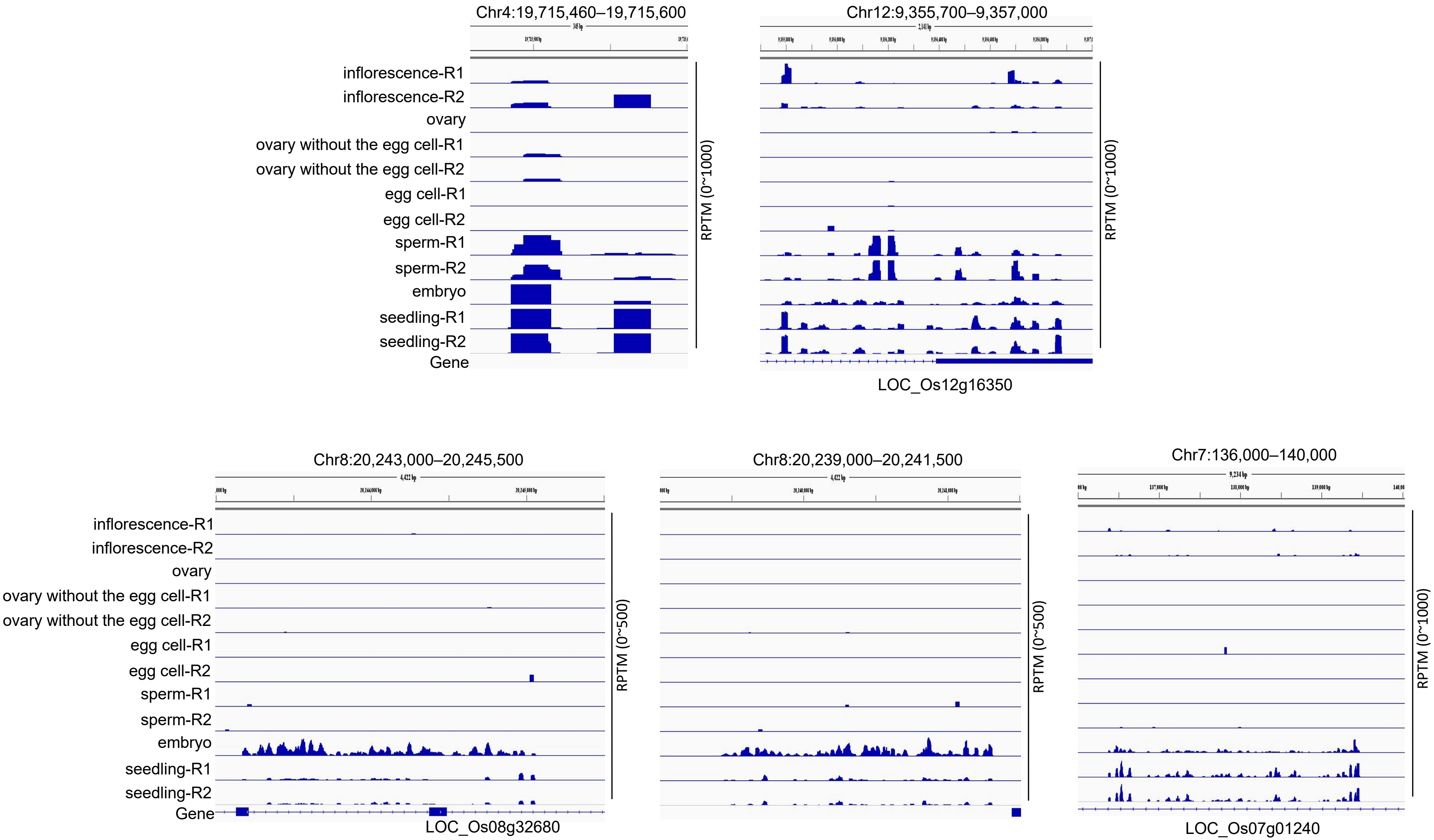

Figure S12. Bill-siRNAs in embryos.
Screen shots of IGV showing 24 nt siRNA abundance on five bill-siRNA clusters in embryos.

Figure S13

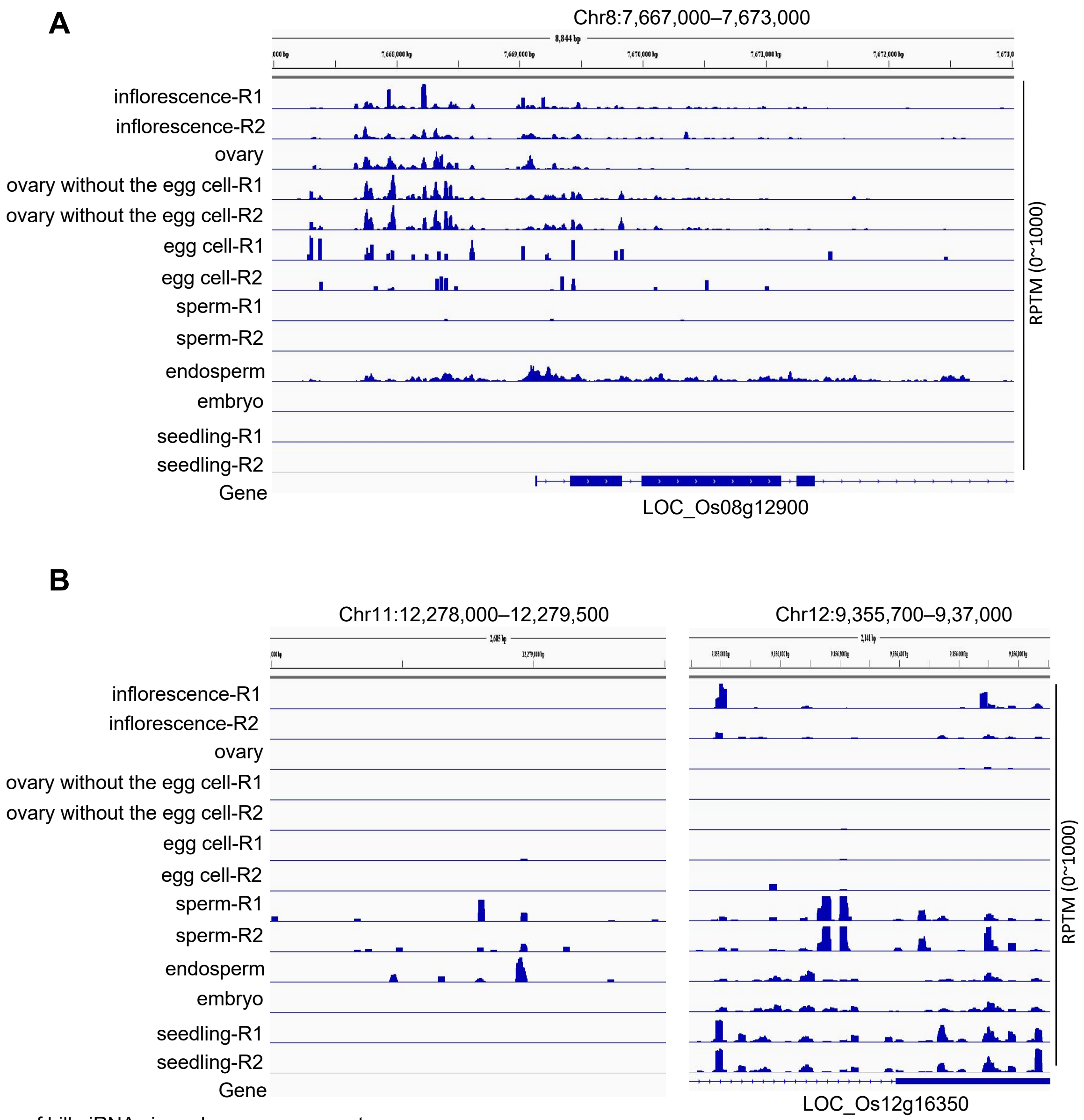

Figure S13. Overlap of bill-siRNAs in endosperm vs. gametes.
A. Screen shot of IGV showing 24 nt siRNA abundance on the common bill-siRNA cluster in endosperm and ovary without the egg cell. **B.** Screen shots of IGV showing 24 nt siRNA abundance on the common bill-siRNA clusters in endosperm and sperm.

Figure S14

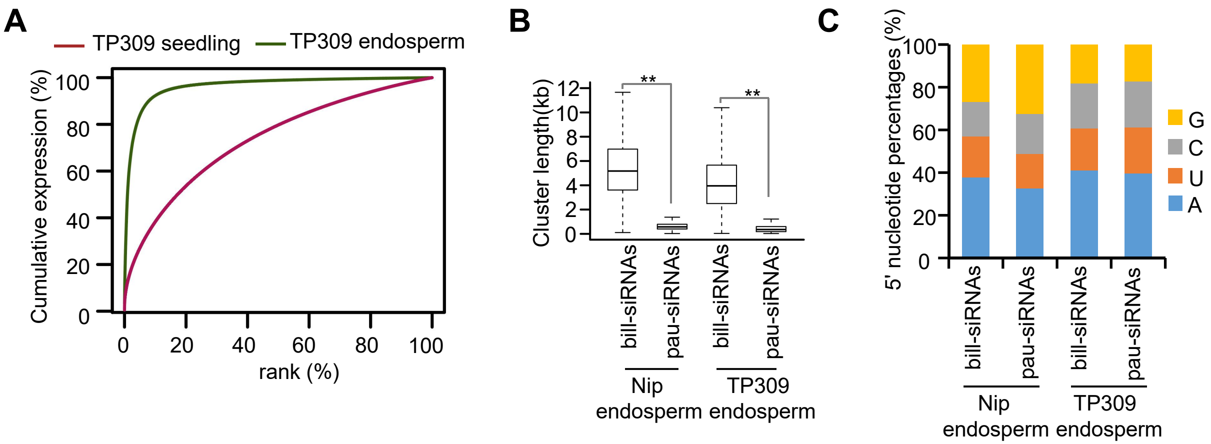

Figure S14. Features of bill-siRNAs in endosperms.
A. Cumulative expression plots of 24 nt siRNAs in TP309 seedlings and TP309 endosperms. **B.** The lengths of bill-siRNA clusters and pau-siRNA clusters in the endosperms of TP309 and Nipponbare. ** indicates p < 0.01 by the Wilcoxon sum test. **C.** Percentage of four bases at the first nucleotide acid of bill-siRNAs and pau-siRNAs in the endosperm of TP309 and Nipponbare.

Figure S15

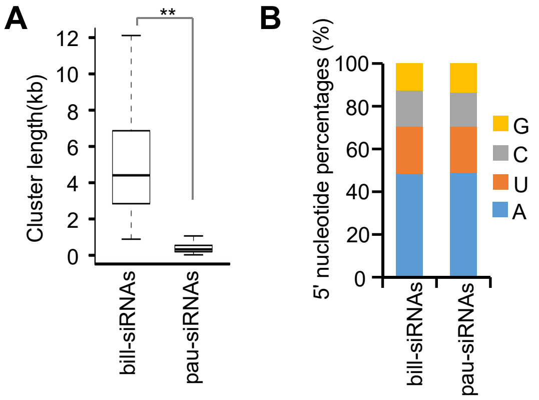

Figure S15. Features of bill-siRNAs in seed coats.
A. The lengths of bill-siRNA clusters and pau-siRNA clusters in the seed coats of TP309. ** indicates $p < 0.01$ by the Wilcoxon sum test. **B.** Percentage of four bases at the first nucleotide acid of bill-siRNAs and pau-siRNAs in seed coats of TP309.
