# Supplementary material for: The Rise and Fall of Billionaire siRNAs during Reproductive Development in Rice
Source: Plants (Basel). 2022 Jul 28;11(15):1957. doi: 10.3390/plants11151957 (PMC9370656; doi:10.3390/plants11151957)
Supplement: Supplementary file 1 [file plants-11-01957-s001.zip › plants-1800426-Tables.pdf]

Table S1 Bill-siRNA clusters list.

Bill-siRNA clusters in Nipponbare seedling

Region	Length	seedling-R1_reads	seedling-R2_reads	seedling-R1_RPTM	seedling-R2_RPTM
Chr10:4833386-4833508	123	4809	3569	2675.71	2208.25
Chr10:10320598-10320961	364	15165	10986	8437.76	6797.37
Chr10:20680292-20680594	303	4078	5602	2268.99	3466.13
Chr12:9355709-9356941	1233	10905	9637	6067.51	5962.7
Chr2:1553148-1553253	106	31543	24242	17550.4	14999.3
Chr2:8522078-8522114	37	13082	7402	7278.79	4579.84
Chr2:8525226-8525258	33	9366	7386	5211.22	4569.94
Chr2:9901657-9902569	913	5514	4584	3067.97	2836.26
Chr3:30515072-30515183	112	3514	5180	1955.18	3205.02
Chr4:19715084-19715249	166	7751	7679	4312.63	4751.23
Chr4:19715478-19715718	241	11876	6365	6607.77	3938.22
Chr5:2810218-2810607	390	5254	4882	2923.31	3020.64
Chr5:3660751-3661764	1014	3804	3090	2116.53	1911.88
Chr6:13330447-13330641	195	7692	5974	4279.81	3696.29
Chr6:21165722-21165845	124	4154	5162	2311.27	3193.88
Chr7:136323-139659	3337	18767	15015	10441.9	9290.23
Chr7:12755873-12756152	280	6113	5097	3401.26	3153.67
Chr7:21302369-21308135	5767	6250	5166	3477.48	3196.36
Chr8:9193386-9194236	851	7256	5675	4037.22	3511.29

Bill-siRNA clusters in Nipponbare inflorescence

Region	Length	inflorescence-R1_reads	inflorescence-R2_reads	inflorescence-R1_RPTM	inflorescence-R2_RPTM
Chr1:2521768-2526052	4285	2627	6895	2904.7	5136.67
Chr1:2555529-2560694	5166	21626	41074	23912.1	30599.5
Chr1:5581732-5589426	7695	1502	3148	1660.78	2345.21
Chr1:8599811-8604128	4318	1747	3329	1931.68	2480.05
Chr1:9038763-9044876	6114	18114	14666	20028.9	10925.9
Chr1:9435862-9439656	3795	23285	35897	25746.5	26742.7
Chr1:10921204-10924098	2895	3046	10412	3368	7756.78
Chr1:10924269-10926494	2226	16187	21196	17898.1	15790.7
Chr1:11189862-11192041	2180	5892	5818	6514.85	4334.32
Chr1:11194294-11201121	6828	10596	18548	11716.1	13818
Chr1:11360420-11365017	4598	30604	38089	33839.2	28375.7
Chr1:11470333-11475439	5107	45398	42784	50197.1	31873.4
Chr1:12139280-12145453	6174	9104	8537	10066.4	6359.93
Chr1:14333154-14340725	7572	42230	59509	46694.2	44333.3
Chr1:14959355-14964119	4765	6862	9715	7587.39	7237.52
Chr1:20809638-20814813	5176	6926	15002	7658.15	11176.3
Chr1:21008537-21022996	14460	5334	8183	5897.86	6096.21
Chr1:25251535-25254752	3218	2172	4074	2401.6	3035.07
Chr1:25311726-25313703	1978	4796	8793	5302.99	6550.65
Chr1:26580342-26582395	2054	1912	2966	2114.12	2209.62
Chr1:28199276-28206541	7266	42822	59660	47348.8	44445.8
Chr1:28209631-28216676	7046	22132	32121	24471.6	23929.6
Chr1:29052975-29057217	4243	8800	12889	9730.26	9602.1
Chr1:30807171-30812220	5050	5617	10653	6210.78	7936.32
Chr1:38341396-38345532	4137	1946	3410	2151.71	2540.4
Chr10:402185-405250	3066	2367	3450	2617.22	2570.2
Chr10:706248-710643	4396	11784	19749	13029.7	14712.7
Chr10:2403049-2413838	10790	3462	9830	3827.97	7323.2
Chr10:3440819-3446842	6024	4065	8004	4494.72	5962.85
Chr10:5924258-5934240	9983	39174	58543	43315.1	43613.6
Chr10:15985572-15992069	6498	19388	27659	21437.5	20605.5
Chr10:16003028-16005127	2100	3952	8460	4369.77	6302.57
Chr10:16005549-16007061	1513	7447	17020	8234.23	12679.6
Chr10:20776781-20779879	3099	9200	10614	10172.5	7907.26
Chr11:2540766-2543924	3159	4721	4690	5220.06	3493.98
Chr11:3230045-3233542	3498	16650	19171	18410.1	14282.1
Chr11:3993179-4002073	8895	5279	10803	5837.05	8048.07
Chr11:7333517-7336095	2579	2861	4631	3163.44	3450.02
Chr11:7725873-7730813	4941	3419	4087	3780.43	3044.75
Chr11:7731057-7743657	12601	76385	104865	84459.7	78122.8
Chr11:8909723-8916209	6487	4887	4271	5403.61	3181.83
Chr11:16329888-16336863	6976	59254	66685	65517.8	49679.3
Chr11:17164662-17170276	5615	11086	20021	12257.9	14915.3
Chr11:20706536-20709509	2974	7801	11964	8625.65	8912.99
Chr11:22338326-22342986	4661	2558	5127	2828.41	3819.53
Chr11:24386946-24396201	9256	42023	44558	46465.3	33195
Chr11:27016652-27019288	2637	6308	7370	6974.83	5490.53
Chr12:2870565-2882229	11665	1829	5785	2022.35	4309.73
Chr12:4726889-4738510	11622	72131	81501	79756	60717
Chr12:4898715-4904135	5421	22455	27006	24828.7	20119
Chr12:4909461-4920790	11330	19298	27242	21338	20294.9
Chr12:5024901-5028293	3393	2208	2386	2441.41	1777.53
Chr12:9061581-9066507	4927	5206	6557	5756.33	4884.86
Chr12:9355708-9356951	1244	2562	1906	2832.83	1419.94

Chr12:10115606-10124335	8730	1665	3571	1841.01	2660.34
Chr12:17499882-17504299	4418	31960	28592	35338.5	21300.6
Chr12:17984443-17988999	4557	11323	17981	12520	13395.6
Chr12:18392996-18397217	4222	5522	6470	6105.74	4820.05
Chr12:18860415-18868291	7877	12392	27162	13702	20235.3
Chr12:19401294-19406902	5609	1840	6295	2034.51	4689.68
Chr12:24724955-24735788	10834	57596	79055	63684.5	58894.7
Chr2:1292570-1294488	1919	3017	2878	3335.93	2144.06
Chr2:1553161-1553270	110	124	14136	137.108	10531.1
Chr2:3700050-3702958	2909	2088	2846	2308.72	2120.23
Chr2:5895693-5907049	11357	36438	39545	40289.9	29460.4
Chr2:6758841-6765525	6685	13325	19739	14733.6	14705.2
Chr2:6795056-6797018	1963	6868	10746	7594.02	8005.6
Chr2:6797248-6798640	1393	5673	5499	6272.7	4096.67
Chr2:7603808-7610062	6255	18680	21215	20654.7	15804.8
Chr2:8343265-8346971	3707	14307	19778	15819.4	14734.3
Chr2:8347074-8350038	2965	2309	3070	2553.09	2287.1
Chr2:13580482-13582726	2245	3397	3513	3756.1	2617.13
Chr2:13582836-13584440	1605	7388	11931	8168.99	8888.41
Chr2:13584554-13586359	1806	10065	10881	11129	8106.17
Chr2:17352298-17362667	10370	3197	7778	3534.96	5794.49
Chr2:19469238-19471202	1965	9594	19400	10608.2	14452.7
Chr2:29633230-29636002	2773	3844	6077	4250.35	4527.27
Chr2:29777060-29783044	5985	5809	9386	6423.08	6992.42
Chr3:12967075-12976151	9077	4072	7419	4502.46	5527.04
Chr3:15609453-15614434	4982	2929	3762	3238.63	2802.63
Chr3:16790240-16793888	3649	2185	3100	2415.98	2309.45
Chr3:17534172-17543694	9523	5416	3953	5988.53	2944.92
Chr3:20789817-20792348	2532	2287	2011	2528.76	1498.16
Chr3:22402292-22412222	9931	42226	42969	46689.8	32011.2
Chr3:24566447-24574082	7636	35515	40864	39269.3	30443
Chr3:24601028-24606935	5908	11969	15266	13234.3	11372.9
Chr3:26123020-26126247	3228	9410	17259	10404.7	12857.7
Chr3:27617297-27621763	4467	9936	16455	10986.3	12258.7
Chr3:28510208-28512514	2307	2359	2888	2608.37	2151.51
Chr3:29751814-29757515	5702	4199	3891	4642.88	2898.73
Chr4:4588570-4593843	5274	1858	4531	2054.41	3375.52
Chr4:11651697-11659198	7502	2907	5874	3214.3	4376.04
Chr4:19681237-19685224	3988	7526	9336	8321.58	6955.17
Chr4:26770125-26774265	4141	1718	5445	1899.61	4056.44
Chr4:35049575-35053502	3928	27643	31088	30565.2	23160.1
Chr5:661784-664204	2421	1627	3393	1798.99	2527.73
Chr5:1132468-1140507	8040	15917	24430	17599.6	18200
Chr5:1680413-1685418	5006	7138	13497	7892.57	10055.1
Chr5:4824684-4831616	6933	9306	14400	10289.7	10727.8
Chr5:10916862-10920212	3351	11707	15457	12944.6	11515.2
Chr5:10920338-10923044	2707	3129	5343	3459.77	3980.45
Chr5:11242416-11247317	4902	30080	44552	33259.8	33190.5
Chr5:16055424-16058088	2665	7045	9093	7789.73	6774.14
Chr5:18245583-18250825	5243	1078	3800	1191.96	2830.94
Chr5:19884414-19887313	2900	2169	3066	2398.29	2284.12
Chr5:25352163-25357799	5637	40783	57544	45094.2	42869.4
Chr5:26871602-26874206	2605	7858	11473	8688.68	8547.2
Chr6:2605685-2612791	7107	1957	5488	2163.88	4088.47
Chr6:6078050-6083497	5448	20077	19599	22199.4	14600.9
Chr6:6420375-6424515	4141	1493	4229	1650.83	3150.54
Chr6:7535124-7539311	4188	20019	29031	22135.2	21627.6
Chr6:11299378-11306310	6933	23604	32064	26099.2	23887.2
Chr6:13424568-13438590	14023	62500	88610	69106.9	66013.1
Chr6:17320067-17323412	3346	3581	3382	3959.55	2519.54
Chr6:19709922-19712027	2106	4907	4784	5425.72	3564
Chr6:21224117-21229217	5101	3124	4682	3454.24	3488.02
Chr6:25786623-25794475	7853	1095	4417	1210.75	3290.6
Chr6:27798029-27809477	11449	32340	44968	35758.7	33500.5
Chr6:28515426-28518629	3204	4926	8748	5446.73	6517.12
Chr6:29376920-29380615	3696	33523	50394	37066.8	37542.7
Chr7:995670-1002285	6616	4879	6773	5394.76	5045.78
Chr7:4346348-4353167	6820	30032	46656	33206.7	34758
Chr7:4372363-4377384	5022	24835	29814	27460.3	22211
Chr7:4703565-4710602	7038	2132	5532	2357.38	4121.25
Chr7:4988771-4998928	10158	3007	8817	3324.87	6568.53
Chr7:8049445-8056572	7128	1068	3893	1180.9	2900.22
Chr7:8291378-8301022	9645	21362	24845	23620.2	18509.1
Chr7:15712491-15717966	5476	7470	11427	8259.66	8512.94
Chr7:15985944-15991071	5128	16937	15273	18727.4	11378.1
Chr7:19997762-20003812	6051	5672	9608	6271.59	7157.81
Chr7:20003935-20006845	2911	1812	5698	2003.55	4244.92
Chr7:21685705-21693930	8226	2016	5768	2229.11	4297.07
Chr7:21714119-21720197	6079	2627	7860	2904.7	5855.58
Chr7:23519975-23522299	2325	8423	10618	9313.4	7910.24
Chr7:27168180-27170308	2129	1967	3615	2174.93	2693.12
Chr8:4307930-4312926	4997	1621	3757	1792.36	2798.91
Chr8:5776237-5781355	5119	11903	17170	13161.3	12791.4
Chr8:7667556-7673918	6363	9803	11376	10839.3	8474.94
Chr8:7762814-7770869	8056	26914	29225	29759.1	21772.2
Chr8:8702577-8702970	394	5578	3552	6167.66	2646.18
Chr8:9443716-9449228	5513	4962	7612	5486.54	5670.82
Chr8:10491663-10502271	10609	32329	35776	35746.5	26652.6

Chr8:10866511-10873300	6790	1033	4378	1142.2	3261.54
Chr8:10890143-10894812	4670	1647	3931	1821.11	2928.53
Chr8:15452051-15462294	10244	88167	101079	97487.2	75302.3
Chr8:15463916-15466938	3023	3818	4648	4221.6	3462.69
Chr8:16973609-16980517	6909	8634	10783	9546.71	8033.17
Chr8:20978669-20986760	8092	9172	21381	10141.6	15928.5
Chr8:21691355-21695670	4316	12615	16536	13948.5	12319.1
Chr8:22467505-22472176	4672	1230	4492	1360.02	3346.47
Chr8:22504024-22508062	4039	1060	4131	1172.05	3077.53
Chr8:27144229-27147566	3338	11362	7860	12563.1	5855.58
Chr9:1290331-1297850	7520	4834	9754	5345.01	7266.58
Chr9:3227798-3229687	1890	2652	3321	2932.35	2474.09
Chr9:3229897-3231863	1967	2360	4564	2609.48	3400.11
Chr9:3510381-3520050	9670	2699	2528	2984.31	1883.32
Chr9:5769286-5778227	8942	1556	4640	1720.49	3456.73
Chr9:9556900-9558463	1564	2417	3567	2672.5	2657.36
Chr9:12418744-12427530	8787	5337	6917	5901.18	5153.06
Chr9:12621918-12623526	1609	3307	3522	3656.59	2623.83
Chr9:15228002-15232300	4299	4830	6579	5340.58	4901.25
Chr9:16533262-16541018	7757	3033	7252	3353.62	5402.63
Chr9:16723081-16727730	4650	29508	39096	32627.3	29125.9

Bill-siRNA clusters in Nipponbare ovary

Region	Length	ovary_reads	ovary_RPTM
Chr1:2521844-2525545	3702	8576	7934.62
Chr1:2555529-2560448	4920	59626	55166.7
Chr1:9039468-9043459	3992	15819	14635.9
Chr1:9435925-9439656	3732	50224	46467.8
Chr1:10922855-10924098	1244	15258	14116.9
Chr1:10924269-10926494	2226	42643	39453.8
Chr1:11188466-11189659	1194	2558	2366.69
Chr1:11189862-11191482	1621	9442	8735.85
Chr1:11195592-11200861	5270	20356	18833.6
Chr1:11360445-11364759	4315	75563	69911.8
Chr1:11470338-11475439	5102	79149	73229.6
Chr1:12141521-12144908	3388	5117	4734.31
Chr1:14333181-14340292	7112	113393	104913
Chr1:14959477-14963426	3950	16419	15191.1
Chr1:20809443-20814598	5156	22228	20565.6
Chr1:21008538-21014877	6340	9650	8928.29
Chr1:25251520-25254709	3190	8514	7877.25
Chr1:25311726-25313703	1978	15834	14649.8
Chr1:26580524-26582469	1946	4094	3787.82
Chr1:28199278-28206541	7264	91550	84703.1
Chr1:28209633-28216658	7026	41598	38487
Chr1:29053164-29056607	3444	16537	15300.2
Chr1:30808064-30812190	4127	17943	16601.1
Chr1:38450707-38453768	3062	4107	3799.84
Chr10:401914-403983	2070	3986	3687.89
Chr10:706272-710633	4362	36959	34194.9
Chr10:5925568-5934240	8673	103078	95369
Chr10:11208276-11213222	4947	2361	2184.42
Chr10:15986726-15991294	4569	35608	32944.9
Chr10:16003038-16005189	2152	5643	5220.97
Chr10:16005552-16007049	1498	19135	17703.9
Chr10:20776792-20779600	2809	19488	18030.5
Chr11:3230045-3232308	2264	34975	32359.3
Chr11:7332657-7335666	3010	10821	10011.7
Chr11:7731054-7735579	4526	114710	106131
Chr11:7735686-7742719	7034	63993	59207.1
Chr11:8910747-8913303	2557	2240	2072.47
Chr11:16330307-16336817	6511	121346	112271
Chr11:17164714-17170193	5480	25690	23768.7
Chr11:19254455-19256354	1900	2546	2355.59
Chr11:20706558-20709374	2817	23173	21439.9
Chr11:22338335-22342969	4635	5928	5484.66
Chr11:24387542-24394638	7097	50954	47143.2
Chr11:27016911-27019288	2378	10212	9448.26
Chr12:4727561-4738238	10678	163098	150900
Chr12:4898742-4904118	5377	40094	37095.4
Chr12:4911982-4917759	5778	40474	37447
Chr12:9061767-9066481	4715	7541	6977.02
Chr12:10927193-10928982	1790	2359	2182.57
Chr12:17500450-17504299	3850	65373	60483.9
Chr12:17984443-17988995	4553	33899	31363.8
Chr12:18392996-18394295	1300	5323	4924.9
Chr12:18395684-18397217	1534	3446	3188.28
Chr12:18860618-18867498	6881	42782	39582.4
Chr12:24728001-24734673	6673	125729	116326
Chr2:3700051-3702965	2915	7078	6548.65
Chr2:5900748-5907257	6510	81371	75285.4
Chr2:6759382-6763679	4298	30285	28020
Chr2:6795024-6797015	1992	16995	15724
Chr2:6797246-6798459	1214	11292	10447.5
Chr2:7605635-7610028	4394	46593	43108.4

Chr2:8343301-8346968	3668	35706	33035.6
Chr2:13580615-13582726	2112	5480	5070.16
Chr2:13582842-13584440	1599	22539	20853.3
Chr2:13584555-13586314	1760	26505	24522.7
Chr2:19469288-19471202	1915	31430	29079.4
Chr2:29633236-29635858	2623	7929	7336
Chr2:29777051-29782774	5724	14196	13134.3
Chr2:34951976-34953742	1767	3282	3036.54
Chr3:12709654-12710829	1176	4169	3857.21
Chr3:12711051-12713166	2116	4022	3721.2
Chr3:15611459-15613388	1930	5607	5187.66
Chr3:16790320-16793973	3654	6140	5680.8
Chr3:19352637-19356624	3988	2932	2712.72
Chr3:20792461-20793650	1190	2294	2122.44
Chr3:22293543-22296227	2685	3385	3131.84
Chr3:22402851-22411450	8600	90234	83485.6
Chr3:24566548-24574110	7563	64241	59436.5
Chr3:24601688-24606560	4873	28588	26450
Chr3:26123012-26126243	3232	32921	30458.9
Chr3:26143500-26144629	1130	2699	2497.15
Chr3:27617781-27619388	1608	7376	6824.36
Chr3:27619511-27621774	2264	18201	16839.8
Chr3:27940887-27943015	2129	2327	2152.97
Chr3:28510208-28512378	2171	8592	7949.42
Chr3:29751814-29754615	2802	2532	2342.64
Chr4:11651952-11657426	5475	11637	10766.7
Chr4:17845592-17848967	3376	2480	2294.53
Chr4:19681575-19685138	3564	18956	17538.3
Chr4:20353517-20355297	1781	2292	2120.59
Chr4:32565887-32567718	1832	3543	3278.03
Chr4:35049584-35053502	3919	68363	63250.2
Chr5:661703-665576	3874	5649	5226.52
Chr5:1134469-1140452	5984	26959	24942.8
Chr5:1680412-1684307	3896	21642	20023.4
Chr5:4824694-4831581	6888	19528	18067.5
Chr5:10917329-10920212	2884	25845	23912.1
Chr5:10920342-10922460	2119	3827	3540.79
Chr5:11242416-11246770	4355	65400	60508.8
Chr5:13079676-13081371	1696	2169	2006.78
Chr5:16055424-16058037	2614	15623	14454.6
Chr5:19884425-19886842	2418	2822	2610.95
Chr5:25352166-25357301	5136	125817	116407
Chr5:26871715-26874206	2492	19220	17782.6
Chr5:27489463-27491629	2167	2724	2520.28
Chr6:6078050-6082334	4285	32313	29896.4
Chr6:7380761-7383367	2607	2818	2607.25
Chr6:7535106-7539308	4203	55255	51122.6
Chr6:11300484-11305697	5214	54265	50206.6
Chr6:13424897-13437332	12436	134932	124841
Chr6:17320233-17323410	3178	5943	5498.53
Chr6:17673971-17676421	2451	5400	4996.14
Chr6:18068855-18071342	2488	3331	3081.88
Chr6:19709919-19712027	2109	6436	5954.66
Chr6:21225606-21227918	2313	4595	4251.35
Chr6:27798395-27809150	10756	70599	65319
Chr6:28515525-28518630	3106	11929	11036.9
Chr6:29376920-29381191	4272	86880	80382.4
Chr7:996003-1001329	5327	10923	10106.1
Chr7:3900364-3903253	2890	3028	2801.54
Chr7:4348715-4353178	4464	78784	72891.9
Chr7:4372345-4376274	3930	51877	47997.2
Chr7:5719332-5723278	3947	3484	3223.44
Chr7:8295242-8301023	5782	37657	34840.7
Chr7:12422161-12424012	1852	2496	2309.33
Chr7:15676996-15677882	887	3912	3619.43
Chr7:15712854-15717796	4943	20012	18515.3
Chr7:15986496-15991075	4580	34511	31930
Chr7:19999609-20003812	4204	12910	11944.5
Chr7:20003949-20005234	1286	2364	2187.2
Chr7:22459903-22462003	2101	3320	3071.7
Chr7:23520073-23522005	1933	11762	10882.3
Chr7:27168208-27170301	2094	6515	6027.75
Chr8:5776604-5780800	4197	23136	21405.7
Chr8:7325192-7326190	999	2426	2244.56
Chr8:7667556-7671308	3753	10352	9577.79
Chr8:7763655-7770869	7215	58782	54385.8
Chr8:8446598-8450020	3423	3270	3025.44
Chr8:9443293-9449278	5986	12350	11426.4
Chr8:10491813-10500449	8637	80024	74039.1
Chr8:15383621-15386899	3279	3012	2786.74
Chr8:15452051-15462294	10244	173350	160385
Chr8:16975162-16979983	4822	21360	19762.5
Chr8:20978754-20986055	7302	22849	21140.2
Chr8:21691366-21695670	4305	29811	27581.5
Chr8:27145005-27147561	2557	17336	16039.5
Chr9:1293310-1297850	4541	11970	11074.8
Chr9:3227834-3229695	1862	5527	5113.65
Chr9:3229897-3231498	1602	4848	4485.43

Chr9:3510498-3519634	9137	3709	3431.61
Chr9:9556907-9558881	1975	6633	6136.93
Chr9:12177687-12182797	5111	4271	3951.58
Chr9:12418744-12420519	1776	2754	2548.03
Chr9:12420622-12427022	6401	8377	7750.5
Chr9:12621913-12623471	1559	4770	4413.26
Chr9:15228199-15232299	4101	8535	7896.68
Chr9:16723081-16726908	3828	83046	76835.1
Chr9:17428907-17430558	1652	3253	3009.71

Bill-siRNA clusters in Nipponbare bract

Region	Length	bract_reads	bract_RPTM
Chr1:2555565-2559538	3974	4123	6730.01
Chr1:9436150-9439635	3486	4821	7869.37
Chr1:10923058-10924089	1032	1276	2082.83
Chr1:10924271-10926493	2223	2889	4715.74
Chr1:11360926-11364464	3539	5937	9691.02
Chr1:11470886-11475397	4512	6158	10051.8
Chr1:14333332-14336317	2986	3844	6274.6
Chr1:14336425-14338650	2226	2863	4673.3
Chr1:14338763-14339975	1213	1559	2544.77
Chr1:25311955-25313671	1717	1256	2050.18
Chr1:28200328-28206531	6204	7851	12815.3
Chr1:28210040-28214677	4638	3098	5056.9
Chr1:29053833-29056279	2447	1261	2058.34
Chr10:707138-709675	2538	2916	4759.82
Chr10:5926931-5932489	5559	7262	11853.8
Chr10:10320909-10320961	53	6372	10401.1
Chr10:15987356-15991138	3783	2420	3950.19
Chr10:16005555-16007061	1507	1587	2590.48
Chr10:20777278-20779305	2028	1670	2725.96
Chr11:3230056-3231775	1720	2408	3930.6
Chr11:7731185-7735578	4394	10341	16879.7
Chr11:7735688-7738615	2928	1499	2446.83
Chr11:7738737-7741735	2999	3051	4980.18
Chr11:9798169-9798270	102	2865	4676.57
Chr11:16332390-16336650	4261	9484	15480.8
Chr11:17165906-17168443	2538	1326	2164.44
Chr11:20706562-20708264	1703	1420	2317.88
Chr11:24389485-24393129	3645	1877	3063.85
Chr11:24393231-24394618	1388	1741	2841.85
Chr12:4729407-4736295	6889	11426	18650.8
Chr12:4736403-4737273	871	1588	2592.11
Chr12:4899116-4901879	2764	2834	4625.97
Chr12:4912411-4914505	2095	1402	2288.5
Chr12:4914640-4916540	1901	1231	2009.37
Chr12:9355714-9356917	1204	3370	5500.88
Chr12:17500453-17503989	3537	4861	7934.66
Chr12:17986446-17988664	2219	1722	2810.84
Chr12:18864068-18866206	2139	2383	3889.79
Chr12:24728452-24730449	1998	4335	7076.06
Chr12:24730552-24733990	3439	7061	11525.7
Chr2:1553161-1553230	70	3659	5972.62
Chr2:5902855-5906581	3727	5921	9664.91
Chr2:6759920-6762767	2848	1824	2977.33
Chr2:7605638-7609291	3654	2589	4226.05
Chr2:8343791-8346311	2521	2333	3808.18
Chr2:8522080-8522114	35	7583	12377.8
Chr2:8525230-8525258	29	8358	13642.8
Chr2:13582846-13584440	1595	1620	2644.34
Chr2:13584567-13586310	1744	1932	3153.62
Chr2:19469624-19471187	1564	2648	4322.36
Chr3:22406877-22410687	3811	7321	11950.1
Chr3:24569582-24572160	2579	4168	6803.47
Chr3:24602670-24605086	2417	1602	2614.96
Chr3:26123106-26126043	2938	2294	3744.52
Chr4:35050618-35053502	2885	3918	6395.39
Chr5:1135893-1139298	3406	1795	2930
Chr5:10917774-10920198	2425	1772	2892.45
Chr5:11242427-11246614	4188	6049	9873.84
Chr5:25352988-25356776	3789	8428	13757.1
Chr6:6078050-6080881	2832	2178	3555.17
Chr6:7535833-7538922	3090	3774	6160.34
Chr6:11302776-11304967	2192	4303	7023.83
Chr6:13330467-13330592	126	5914	9653.48
Chr6:13425429-13427842	2414	1398	2281.97
Chr6:13427987-13432273	4287	10103	16491.2
Chr6:27802758-27808793	6036	5718	9333.55
Chr6:29377010-29380073	3064	7899	12893.6
Chr7:136355-139466	3112	3167	5169.53
Chr7:4348789-4353078	4290	5607	9152.36
Chr7:4373867-4376294	2428	4072	6646.77
Chr7:8295263-8298599	3337	2495	4072.61
Chr7:12756069-12756150	82	11019	17986.4
Chr7:15987271-15990551	3281	1908	3114.45

Chr8:5778379-5780180	1802	1256	2050.18
Chr8:7767689-7769795	2107	2632	4296.24
Chr8:10492041-10499590	7550	5737	9364.56
Chr8:15453469-15462092	8624	16232	26495.7
Chr8:20899837-20900339	503	3476	5673.91
Chr8:20981115-20985580	4466	1779	2903.88
Chr8:21692748-21694270	1523	1252	2043.65
Chr8:26181375-26181458	84	1988	3245.03
Chr8:27145500-27147348	1849	1274	2079.56
Chr9:16723103-16726642	3540	6266	10228.1

Bill-siRNA clusters in Nipponbare branch

Region	Length	branch_reads	branch_RPTM
Chr1:9436509-9439638	3130	3249	3303.95
Chr1:11361251-11363743	2493	2445	2486.35
Chr1:14333568-14336302	2735	2395	2435.51
Chr1:28200325-28202175	1851	2038	2072.47
Chr1:28202292-28203296	1005	1991	2024.68
Chr1:30298209-30298439	231	2883	2931.76
Chr10:5928582-5932457	3876	3574	3634.45
Chr10:10320909-10320938	30	8915	9065.79
Chr10:20859032-20859299	268	2600	2643.98
Chr11:7731940-7734461	2522	4190	4260.87
Chr11:9798167-9798290	124	4244	4315.78
Chr11:16312182-16312395	214	2779	2826
Chr11:16332382-16336650	4269	5134	5220.84
Chr11:20715970-20716088	119	2113	2148.74
Chr12:4733378-4736277	2900	4015	4082.91
Chr12:9355709-9356885	1177	3923	3989.35
Chr12:24728404-24730422	2019	2153	2189.42
Chr12:24730552-24732124	1573	2459	2500.59
Chr2:1553161-1553230	70	3836	3900.88
Chr2:5904975-5906828	1854	2126	2161.96
Chr2:8522083-8522114	32	17276	17568.2
Chr2:8525232-8525258	27	14025	14262.2
Chr2:9902423-9902539	117	2226	2263.65
Chr2:17987103-17987205	103	3197	3251.07
Chr2:25352977-25353145	169	2020	2054.17
Chr3:22408354-22409951	1598	2631	2675.5
Chr4:8302174-8302676	503	2289	2327.72
Chr4:23615771-23615947	177	3432	3490.05
Chr5:11242587-11245292	2706	3092	3144.3
Chr5:25353338-25356925	3588	4843	4924.91
Chr6:11302835-11304809	1975	2253	2291.11
Chr6:13330467-13330592	126	9724	9888.47
Chr6:13427996-13430856	2861	4572	4649.33
Chr6:27801821-27806496	4676	3176	3229.72
Chr6:29377010-29380135	3126	5106	5192.36
Chr7:136365-138754	2390	3892	3957.83
Chr7:4348715-4353061	4347	2255	2293.14
Chr7:12756069-12756150	82	16672	16954
Chr8:10496169-10500151	3983	2982	3032.44
Chr8:15454505-15458779	4275	4634	4712.38
Chr8:15459026-15462089	3064	3980	4047.32
Chr8:20900013-20900339	327	4312	4384.93
Chr8:26181386-26181458	73	4657	4735.77
Chr9:10619367-10619814	448	5406	5497.44
Chr9:14466940-14467068	129	2120	2155.86
Chr9:16723927-16726377	2451	2573	2616.52

Bill-siRNA clusters in Nipponbare stamen

Region	Length	stamen_reads	stamen_RPTM
Chr1:2555565-2559450	3886	3265	3551.79
Chr1:9436150-9439650	3501	5611	6103.85
Chr1:10924273-10926493	2221	2586	2813.15
Chr1:11361161-11364515	3355	6781	7376.62
Chr1:11470690-11475204	4515	5718	6220.25
Chr1:13389805-13392416	2612	25244	27461.4
Chr1:14333509-14338649	5141	6135	6673.88
Chr1:22638675-22639984	1310	7299	7940.12
Chr1:28200187-28206540	6354	8235	8958.34
Chr1:28212846-28214674	1829	2312	2515.08
Chr10:707125-709080	1956	2088	2271.4
Chr10:5863800-5864813	1014	2375	2583.61
Chr10:5928579-5932459	3881	6747	7339.63
Chr10:15987295-15991263	3969	2378	2586.88
Chr11:959469-960198	730	13827	15041.5
Chr11:3230045-3232270	2226	2166	2356.25
Chr11:6403924-6404788	865	4097	4456.87
Chr11:7731178-7734501	3324	9344	10164.7
Chr11:7735700-7741734	6035	4500	4895.27

Chr11:16332252-16336558	4307	8824	9599.07
Chr11:24388697-24392981	4285	2086	2269.23
Chr11:24393090-24394620	1531	2234	2430.23
Chr12:4729451-4736278	6828	10722	11663.8
Chr12:4898973-4901944	2972	2704	2941.51
Chr12:9355713-9356881	1169	5112	5561.02
Chr12:17500947-17503828	2882	4643	5050.83
Chr12:17985691-17988719	3029	2121	2307.3
Chr12:18864068-18866169	2102	2084	2267.05
Chr12:23211154-23211743	590	5915	6434.55
Chr12:24728404-24730449	2046	4349	4731
Chr12:24730559-24732147	1589	5740	6244.18
Chr12:24732257-24734673	2417	1956	2127.81
Chr2:5903208-5906599	3392	6649	7233.03
Chr2:6001388-6001878	491	2027	2205.05
Chr2:7606171-7609153	2983	2445	2659.76
Chr2:8343697-8346273	2577	2276	2475.92
Chr2:8522078-8522112	35	5385	5858
Chr2:8525230-8525258	29	6078	6611.87
Chr2:11111727-11114927	3201	3679	4002.15
Chr2:13584569-13586310	1742	2063	2244.21
Chr3:22087978-22088202	225	2519	2740.26
Chr3:22406873-22410756	3884	8475	9219.42
Chr3:24569592-24572543	2952	4850	5276.01
Chr3:26123107-26126009	2903	2041	2220.27
Chr3:29756833-29756968	136	5475	5955.91
Chr4:35050618-35053500	2883	4672	5082.37
Chr5:11242434-11246731	4298	7231	7866.15
Chr5:17801991-17802387	397	5077	5522.95
Chr5:18834207-18834628	422	1844	2005.97
Chr5:25353010-25356767	3758	7966	8665.71
Chr6:6078051-6080845	2795	1849	2011.41
Chr6:7535842-7538898	3057	3429	3730.19
Chr6:11301671-11304902	3232	3807	4141.39
Chr6:12837371-12838522	1152	22789	24790.7
Chr6:13330471-13330581	111	8032	8737.5
Chr6:13427985-13432522	4538	10439	11355.9
Chr6:25366417-25367331	915	9748	10604.2
Chr6:25428066-25428776	711	2365	2572.73
Chr6:27806584-27808759	2176	2943	3201.5
Chr6:29377008-29380144	3137	8271	8997.5
Chr7:4348764-4353078	4315	4637	5044.3
Chr7:4373862-4376239	2378	4095	4454.69
Chr7:8295416-8298545	3130	2651	2883.86
Chr7:12756071-12756150	80	3409	3708.44
Chr7:15987454-15990559	3106	2322	2525.96
Chr7:20909518-20911172	1655	1983	2157.18
Chr7:21303225-21305661	2437	2684	2919.75
Chr7:21306172-21307790	1619	2392	2602.11
Chr8:7767690-7769893	2204	3225	3508.27
Chr8:10495239-10499777	4539	5837	6349.7
Chr8:15453536-15462092	8557	14941	16253.4
Chr8:17681948-17684640	2693	20485	22284.3
Chr8:20899838-20900339	502	9083	9880.82
Chr9:13621908-13624578	2671	11886	12930
Chr9:16723863-16726352	2490	7656	8328.48

Bill-siRNA clusters in egg cell

Region	Length	egg cell-R1_reads	egg cell-R2_reads	egg cell-R1_RPTM	egg cell-R2_RPTM
Chr1:2522950-2523954	1005	4726	4788	6833.04	5751.93
Chr1:2555829-2559377	3549	14865	26180	21492.4	31450.6
Chr1:9040092-9043499	3408	8061	10931	11654.9	13131.6
Chr1:9436970-9439961	2992	17685	20722	25569.7	24893.8
Chr1:10923006-10924104	1099	2326	4209	3363.03	5056.36
Chr1:10924312-10925048	737	2648	2286	3828.59	2746.22
Chr1:10925194-10927397	2204	11318	11145	16364	13388.7
Chr1:11189946-11190873	928	6723	8624	9720.39	10360.2
Chr1:11197266-11199494	2229	10195	9524	14740.3	11441.4
Chr1:11361738-11363092	1355	4979	5800	7198.84	6967.67
Chr1:11363203-11364288	1086	8886	16517	12847.7	19842.2
Chr1:11470883-11473390	2508	13792	17839	19941	21430.4
Chr1:11473902-11475185	1284	1399	1683	2022.73	2021.82
Chr1:12142417-12144054	1638	6182	8011	8938.19	9623.79
Chr1:14333578-14334046	469	1173	2157	1695.97	2591.25
Chr1:14334352-14335941	1590	8856	9844	12804.4	11825.8
Chr1:14336432-14336941	510	8351	7371	12074.2	8854.94
Chr1:14337055-14339725	2671	20107	20787	29071.5	24971.9
Chr1:14960897-14961724	828	5880	7227	8501.54	8681.95
Chr1:20810478-20811091	614	2338	656	3380.38	788.067
Chr1:21009371-21009848	478	755	2475	1091.61	2973.27
Chr1:25252473-25253744	1272	3509	5305	5073.45	6373.01
Chr1:25311930-25312933	1004	10530	11196	15224.7	13450
Chr1:26580979-26581884	906	3255	9274	4706.21	11141.1

Chr1:28200989-28203198	2210	9104	11205	13162.9	13460.8
Chr1:28204077-28206320	2244	9471	15498	13693.6	18618.1
Chr1:28212263-28214868	2606	9027	12893	13051.6	15488.6
Chr1:29053819-29055157	1339	5335	7400	7713.56	8889.78
Chr1:30809420-30809877	458	1469	2335	2123.94	2805.09
Chr1:38451891-38452661	771	3087	4872	4463.31	5852.84
Chr10:402640-402983	344	2216	2079	3203.98	2497.55
Chr10:403437-404183	747	1599	1957	2311.9	2350.99
Chr10:707103-708021	919	2835	2983	4098.96	3583.54
Chr10:708360-710346	1987	7537	13322	10897.3	16004
Chr10:5928572-5929230	659	1485	3082	2147.07	3702.47
Chr10:5929396-5930428	1033	9237	18827	13355.2	22617.3
Chr10:5930578-5932403	1826	5800	9652	8385.88	11595.2
Chr10:5932575-5933433	859	1358	1804	1963.45	2167.18
Chr10:5933640-5934228	589	2975	3615	4301.38	4342.78
Chr10:15987538-15989571	2034	11737	13576	16969.8	16309.1
Chr10:15989724-15991243	1520	5925	10845	8566.61	13028.3
Chr10:16004591-16005109	519	1348	1832	1948.99	2200.82
Chr10:16005560-16006254	695	3380	3252	4886.94	3906.7
Chr10:20777395-20778647	1253	26912	24909	38910.5	29923.7
Chr11:3230496-3231673	1178	7337	8912	10608.1	10706.2
Chr11:7732371-7734499	2129	13395	17827	19367	21416
Chr11:7737199-7738621	1423	3485	5942	5038.75	7138.25
Chr11:7739200-7740174	975	2880	3809	4164.02	4575.83
Chr11:7740288-7741683	1396	15002	18459	21690.5	22175.2
Chr11:16332411-16334505	2095	13438	17857	19429.2	21452
Chr11:16334619-16336288	1670	5588	7168	8079.36	8611.07
Chr11:17165586-17168390	2805	9818	13279	14195.3	15952.4
Chr11:17168610-17169447	838	2277	1559	3292.18	1872.86
Chr11:20707192-20707759	568	2756	4520	3984.74	5429.97
Chr11:22340361-22342289	1929	2614	5209	3779.43	6257.69
Chr11:27017983-27018652	670	1294	2713	1870.92	3259.19
Chr12:4730656-4732631	1976	10010	12007	14472.9	14424.3
Chr12:4732744-4736294	3551	11696	16271	16910.6	19546.7
Chr12:4736472-4737252	781	2312	3480	3342.78	4180.6
Chr12:4899631-4901143	1513	10440	12813	15094.6	15392.5
Chr12:4901316-4901606	291	1964	1059	2839.63	1272.2
Chr12:4912418-4916123	3706	7116	12651	10288.6	15197.9
Chr12:9064216-9066039	1824	11450	17030	16554.9	20458.5
Chr12:17500856-17503815	2960	10579	16080	15295.5	19317.3
Chr12:17986373-17987071	699	779	2801	1126.31	3364.9
Chr12:17987560-17988617	1058	1423	2481	2057.43	2980.48
Chr12:18864100-18866181	2082	13106	17062	18949.2	20497
Chr12:24728425-24731900	3476	34136	44835	49355.2	53861.3
Chr12:24732062-24733391	1330	2054	3461	2969.76	4157.77
Chr2:5903234-5906580	3347	26156	31216	37817.4	37500.5
Chr2:6760060-6761418	1359	2976	3552	4302.82	4267.1
Chr2:6795727-6796808	1082	2782	7058	4022.33	8478.93
Chr2:7606384-7607452	1069	1928	2601	2787.58	3124.64
Chr2:7607595-7608737	1143	2849	1682	4119.2	2020.62
Chr2:8343889-8345013	1125	3414	8712	4936.1	10465.9
Chr2:13582025-13586312	4288	25208	35415	36446.7	42544.8
Chr2:19282892-19283498	607	1812	3713	2619.86	4460.51
Chr2:19469510-19470847	1338	7337	14268	10608.1	17140.5
Chr2:29633288-29635396	2109	8877	12356	12834.7	14843.5
Chr2:29635511-29635720	210	1233	2412	1782.72	2897.59
Chr2:29779279-29779835	557	1563	1982	2259.85	2381.02
Chr2:29781249-29782469	1221	4213	4070	6091.33	4889.38
Chr3:7207362-7208457	1096	1457	2240	2106.59	2690.96
Chr3:12711094-12711820	727	3514	1892	5080.68	2272.9
Chr3:17446092-17447056	965	1512	3015	2186.11	3621.99
Chr3:22406172-22407693	1522	4094	6813	5919.27	8184.61
Chr3:22407803-22408245	443	3207	1477	4636.81	1774.35
Chr3:22408374-22410031	1658	9996	14958	14452.6	17969.4
Chr3:24567201-24568605	1405	1547	1767	2236.72	2122.74
Chr3:24568877-24569967	1091	2032	1582	2937.95	1900.49
Chr3:24570108-24571360	1253	8992	9699	13001	11651.6
Chr3:24571486-24572742	1257	3243	1347	4688.86	1618.18
Chr3:24603917-24604988	1072	3840	3373	5552.03	4052.06
Chr3:26123815-26125355	1541	4064	6296	5875.9	7563.52
Chr3:27619566-27620339	774	4519	7518	6533.75	9031.54
Chr3:27620449-27620718	270	1615	1956	2335.03	2349.79
Chr3:29752343-29753108	766	1120	2377	1619.34	2855.54
Chr4:19682734-19683565	832	1861	1766	2690.71	2121.53
Chr4:20353634-20354745	1112	3432	5432	4962.12	6525.58
Chr4:35050627-35052675	2049	6060	13031	8761.79	15654.4
Chr5:1135610-1136163	554	1664	1636	2405.88	1965.36
Chr5:1136682-1138058	1377	5494	8078	7943.45	9704.28
Chr5:1681798-1682999	1202	8668	7282	12532.5	8748.03
Chr5:4826745-4827921	1177	4936	5945	7136.67	7141.86
Chr5:4828081-4829668	1588	3896	6086	5632.99	7311.24
Chr5:10917881-10920093	2213	6050	6815	8747.34	8187.01
Chr5:10920672-10921539	868	878	3720	1269.45	4468.92
Chr5:11242587-11245267	2681	17267	17846	24965.3	21438.8
Chr5:11245732-11246565	834	1700	2930	2457.93	3519.87
Chr5:16055522-16056984	1463	4339	5368	6273.5	6448.7
Chr5:25353784-25354107	324	1622	3193	2345.15	3835.82
Chr5:25354293-25356551	2259	20009	19150	28929.8	23005.3

Chr5:26873017-26874190	1174	3240	4279	4684.52	5140.46
Chr6:6077897-6080386	2490	16069	18224	23233.2	21892.9
Chr6:7535831-7536909	1079	1892	2189	2735.53	2629.69
Chr6:7537014-7537622	609	6839	7519	9888.1	9032.74
Chr6:7538323-7539394	1072	1866	3147	2697.94	3780.56
Chr6:11301685-11304877	3193	24664	31894	35660.2	38315
Chr6:13426951-13427609	659	5660	4824	8183.46	5795.18
Chr6:13427912-13430860	2949	22245	29534	32162.7	35479.8
Chr6:13430970-13432240	1271	2369	2155	3425.2	2588.85
Chr6:16892428-16893253	826	2106	963	3044.94	1156.87
Chr6:17674091-17674798	708	1756	1814	2538.9	2179.2
Chr6:19710389-19711766	1378	1145	3178	1655.49	3817.8
Chr6:20384471-20384850	380	1967	2013	2843.97	2418.26
Chr6:21225678-21227227	1550	2745	3824	3968.83	4593.85
Chr6:27802387-27803890	1504	2129	3009	3078.19	3614.78
Chr6:27805460-27806391	932	4687	6784	6776.65	8149.77
Chr6:27807201-27807559	359	2777	1775	4015.1	2132.35
Chr6:27807727-27808889	1163	3774	3936	5456.6	4728.4
Chr6:28516353-28517472	1120	4112	7372	5945.3	8856.14
Chr6:28517577-28517890	314	1759	3153	2543.23	3787.77
Chr6:29377012-29380350	3339	38442	53543	55581	64322.4
Chr7:999852-1000769	918	2068	1074	2990	1290.22
Chr7:4349415-4352710	3296	26302	38171	38028.5	45855.7
Chr7:4373867-4375891	2025	7866	8865	11373	10649.7
Chr7:8296498-8297585	1088	6463	8564	9344.47	10288.1
Chr7:15677012-15677890	879	4108	6106	5939.51	7335.27
Chr7:15714989-15715711	723	2389	3085	3454.11	3706.08
Chr7:15988106-15990391	2286	3681	4983	5322.14	5986.19
Chr7:20002141-20002806	666	3256	5696	4707.66	6842.73
Chr7:24243640-24243673	34	2276	2404	3290.73	2887.98
Chr7:26333237-26333261	25	3882	1270	5612.75	1525.68
Chr8:5778355-5779406	1052	1842	2964	2663.24	3560.72
Chr8:7667731-7668633	903	2722	1811	3935.58	2175.59
Chr8:7767913-7768467	555	1231	2880	1779.83	3459.81
Chr8:7768631-7769265	635	2075	1797	3000.12	2158.78
Chr8:7991188-7991898	711	2000	1476	2891.68	1773.15
Chr8:9445368-9447251	1884	8798	7822	12720.5	9396.74
Chr8:10495804-10497082	1279	2651	2510	3832.92	3015.32
Chr8:10497183-10497715	533	2867	2755	4145.22	3309.64
Chr8:10498104-10499006	903	1609	4455	2326.36	5351.89
Chr8:15455335-15458898	3564	21966	34086	31759.3	40948.3
Chr8:15459018-15462222	3205	25805	32779	37309.9	39378.1
Chr8:16976766-16978189	1424	3484	3550	5037.31	4264.69
Chr8:20981399-20983484	2086	7629	8878	11030.3	10665.3
Chr8:21691538-21694272	2735	8081	10269	11683.8	12336.4
Chr8:27145500-27146791	1292	2058	2817	2975.54	3384.12
Chr9:1293771-1295052	1282	3580	3650	5176.11	4384.82
Chr9:1295658-1296838	1181	5072	6749	7333.3	8107.72
Chr9:1297039-1297441	403	1840	2829	2660.35	3398.54
Chr9:3228857-3229750	894	1549	3611	2239.61	4337.97
Chr9:3229904-3230932	1029	2311	3798	3341.34	4562.62
Chr9:3515553-3516049	497	2491	3113	3601.59	3739.71
Chr9:12420612-12422305	1694	2113	4114	3055.06	4942.24
Chr9:15228668-15229487	820	3397	2397	4911.52	2879.57
Chr9:15230413-15231047	635	1989	2375	2875.78	2853.14
Chr9:16724057-16726642	2586	12227	16418	17678.3	19723.3

Bill-siRNA clusters in ovary without the egg cell

Region	Length	ovary without the egg cell-R1_reads	ovary without the egg cell-R2_reads	ovary without the egg cell-R1_RPTM	ovary without the egg cell-R2_RPTM
Chr1:2521732-2523864	2133	25792	27713	12743.7	12906.4
Chr1:2555230-2559488	4259	117278	121959	57946.3	56798.3
Chr1:9039469-9044839	5371	99102	104707	48965.7	48763.8
Chr1:9436039-9441447	5409	127699	134266	63095.3	62529.9
Chr1:10922454-10924142	1689	19058	19364	9416.44	9018.14
Chr1:10924269-10928133	3865	94029	102110	46459.1	47554.3
Chr1:11187953-11189724	1772	4081	4418	2016.4	2057.54
Chr1:11189849-11190961	1113	26810	28074	13246.7	13074.5
Chr1:11194472-11200782	6311	85568	89910	42278.6	41872.6
Chr1:11360842-11365015	4174	125122	131850	61822	61404.8
Chr1:11468957-11470040	1084	4341	4446	2144.86	2070.58
Chr1:11470426-11476572	6147	124839	136202	61682.2	63431.5
Chr1:12141609-12145052	3444	42954	45332	21223.3	21111.9
Chr1:14333307-14340220	6914	239314	254048	118244	118314
Chr1:14959357-14963605	4249	31559	33786	15593.1	15734.7
Chr1:16172286-16175087	2802	5397	5412	2666.62	2520.46
Chr1:20809793-20814418	4626	22562	23360	11147.7	10879.1
Chr1:21008411-21013112	4702	20818	22323	10286	10396.2
Chr1:25251535-25254062	2528	18455	19004	9118.5	8850.48
Chr1:25311931-25313593	1663	46192	49745	22823.2	23167.1
Chr1:26580919-26582394	1476	14889	15190	7356.56	7074.24
Chr1:28199338-28208011	8674	145728	154552	72003.3	71977.5
Chr1:28209610-28215997	6388	87100	91800	43035.6	42752.8

Chr1:29053251-29056279	3029	39794	42304	19662	19701.7
Chr1:30808356-30812188	3833	19972	21713	9868.04	10112.1
Chr1:38451520-38453329	1810	7283	7522	3598.48	3503.12
Chr1:39322744-39324043	1300	4622	4329	2283.7	2016.09
Chr10:401896-404258	2363	14738	15692	7281.95	7308.03
Chr10:707091-710421	3331	73556	78365	36343.6	36495.9
Chr10:3442273-3445803	3531	15044	16629	7433.14	7744.4
Chr10:5680286-5681100	815	4064	4552	2008	2119.94
Chr10:5927422-5933485	6064	139501	148796	68926.6	69296.8
Chr10:5933589-5934237	649	12298	13223	6076.36	6158.17
Chr10:11208741-11212837	4097	6272	6474	3098.96	3015.05
Chr10:15986188-15991421	5234	96484	100871	47672.1	46977.3
Chr10:16004085-16005176	1092	8536	9111	4217.58	4243.15
Chr10:16005552-16007432	1881	31814	35343	15719.1	16459.8
Chr10:20777019-20779416	2398	113607	121578	56132.5	56620.9
Chr11:2541243-2543960	2718	11251	11590	5559.05	5397.66
Chr11:3229550-3232287	2738	67343	70309	33273.7	32744.1
Chr11:7728254-7730775	2522	5204	5363	2571.26	2497.64
Chr11:7730927-7734524	3598	117447	124976	58029.8	58203.4
Chr11:7734625-7738644	4020	35328	37162	17455.3	17307
Chr11:7738747-7741713	2967	95366	100039	47119.7	46589.8
Chr11:7741872-7742581	710	5111	5383	2525.31	2506.95
Chr11:8911397-8913713	2317	8341	8805	4121.24	4100.64
Chr11:9698554-9701870	3317	5151	5230	2545.08	2435.7
Chr11:15465328-15468157	2830	4817	5358	2380.05	2495.31
Chr11:16330812-16332117	1306	7926	8130	3916.19	3786.28
Chr11:16332244-16336650	4407	182633	195401	90237.8	91001.5
Chr11:17164417-17170500	6084	60600	64927	29942.1	30237.6
Chr11:19254502-19256108	1607	6605	6951	3263.49	3237.2
Chr11:20707158-20709525	2368	22795	23395	11262.9	10895.4
Chr11:22336837-22342925	6089	25588	27563	12642.9	12836.6
Chr11:26270584-26272400	1817	11338	12645	5602.03	5888.99
Chr11:27016958-27019330	2373	13644	14267	6741.41	6644.38
Chr12:4729317-4736344	7028	179194	190803	88538.6	88860.1
Chr12:4736450-4737273	824	24049	26477	11882.5	12330.8
Chr12:4737375-4738238	864	6536	6957	3229.4	3239.99
Chr12:4898892-4904926	6035	79496	82498	39278.5	38420.7
Chr12:4912031-4918086	6056	71321	78018	35239.3	36334.3
Chr12:5024509-5027632	3124	5964	6040	2946.77	2812.93
Chr12:9062955-9066535	3581	42003	45331	20753.4	21111.4
Chr12:17500242-17505707	5466	92036	97679	45474.4	45490.7
Chr12:17984344-17989083	4740	49369	51788	24392.9	24118.5
Chr12:18861253-18866953	5701	58469	60505	28889.2	28178.2
Chr12:19232994-19236259	3266	10611	11316	5242.83	5270.05
Chr12:21104064-21108755	4692	17841	18604	8815.12	8664.19
Chr12:23677972-23679294	1323	9121	10066	4506.63	4687.9
Chr12:24727729-24734587	6859	245861	261174	121478	121633
Chr2:1371636-1372683	1048	4034	4433	1993.17	2064.52
Chr2:3700044-3702409	2366	5538	6218	2736.29	2895.83
Chr2:5901327-5907223	5897	184012	193662	90919.2	90191.6
Chr2:6759591-6763892	4302	34240	36178	16917.8	16848.7
Chr2:6795192-6797025	1834	31811	33256	15717.6	15487.9
Chr2:6797249-6798333	1085	14218	14342	7025.02	6679.31
Chr2:7604912-7609291	4380	49521	52816	24468	24597.3
Chr2:8343847-8349482	5636	58777	59609	29041.3	27760.9
Chr2:13139422-13139605	184	9032	10693	4462.65	4979.91
Chr2:13580566-13587717	7152	166166	177286	82101.6	82565.1
Chr2:14731925-14734996	3072	4658	4733	2301.49	2204.24
Chr2:19282158-19285083	2926	20127	22437	9944.62	10449.3
Chr2:19469359-19471985	2627	85198	90752	42095.8	42264.7
Chr2:29632213-29635840	3628	33514	35512	16559.1	16538.5
Chr2:29778338-29779853	1516	8168	8306	4035.76	3868.24
Chr2:29780011-29782774	2764	28809	31013	14234.3	14443.3
Chr2:33666809-33669145	2337	5582	6427	2758.03	2993.16
Chr3:7204980-7209458	4479	9794	10801	4839.15	5030.21
Chr3:10131626-10135220	3595	5586	6749	2760.01	3143.12
Chr3:12710048-12712968	2921	14962	15140	7392.63	7050.95
Chr3:15609540-15613989	4450	9495	9515	4691.42	4431.29
Chr3:16165123-16166316	1194	6870	7083	3394.42	3298.67
Chr3:16790754-16793684	2931	11280	11636	5573.38	5419.08
Chr3:17443800-17448110	4311	8285	8643	4093.57	4025.19
Chr3:19353012-19356617	3606	5957	6547	2943.32	3049.05
Chr3:20789900-20793672	3773	12978	13437	6412.35	6257.84
Chr3:22294438-22296091	1654	5391	6088	2663.66	2835.28
Chr3:22402927-22411016	8090	125392	133733	61955.4	62281.7
Chr3:24566476-24573767	7292	114465	120698	56556.4	56211.1
Chr3:24601609-24605533	3925	44726	46800	22098.8	21795.5
Chr3:26122885-26129251	6367	46363	50210	22907.7	23383.6
Chr3:26143572-26144672	1101	8143	8396	4023.4	3910.16
Chr3:27618891-27621672	2782	54256	56062	26807.5	26109
Chr3:28509601-28511671	2071	11802	13043	5831.29	6074.34
Chr3:29748836-29751620	2785	7512	7831	3711.63	3647.03
Chr3:29751734-29754695	2962	21294	22183	10521.2	10331
Chr4:11651843-11656585	4743	12867	14059	6357.5	6547.51
Chr4:19682184-19684644	2461	20203	21938	9982.17	10216.9
Chr4:20353577-20355903	2327	12826	12878	6337.25	5997.5
Chr4:23338306-23340568	2263	5438	5682	2686.88	2646.2
Chr4:27933005-27934489	1485	5932	6474	2930.96	3015.05

Chr4:35050584-35053521	2938	85988	89688	42486.1	41769.2
Chr5:661691-664101	2411	9504	9830	4695.87	4578
Chr5:1134949-1136537	1589	10082	9930	4981.45	4624.57
Chr5:1136676-1140186	3511	45340	48572	22402.2	22620.8
Chr5:1680136-1683572	3437	28844	30197	14251.6	14063.2
Chr5:2481362-2481985	624	6976	7499	3446.8	3492.41
Chr5:2775827-2775910	84	5699	5769	2815.84	2686.72
Chr5:4826150-4827939	1790	21199	23453	10474.3	10922.5
Chr5:4828053-4831638	3586	25460	26847	12579.6	12503.1
Chr5:10917366-10922456	5091	68917	70303	34051.5	32741.3
Chr5:11241193-11247100	5908	132179	140517	65308.8	65441.1
Chr5:16053876-16057988	4113	26728	27831	13206.1	12961.4
Chr5:19883443-19885047	1605	4851	5651	2396.85	2631.77
Chr5:25352931-25357226	4296	139747	146788	69048.1	68361.6
Chr5:26872169-26874574	2406	27974	29986	13821.8	13965
Chr5:27488831-27490515	1685	9545	9961	4716.12	4639
Chr6:1020770-1023328	2559	4717	5171	2330.64	2408.22
Chr6:1050396-1053374	2979	5912	6909	2921.08	3217.64
Chr6:6073665-6081727	8063	96037	101091	47451.3	47079.8
Chr6:7378759-7383285	4527	10260	11250	5069.4	5239.31
Chr6:7535649-7541170	5522	93699	98122	46296.1	45697.1
Chr6:11301673-11305097	3425	161179	173127	79637.5	80628.1
Chr6:13425328-13433837	8510	196334	213137	97007.4	99261.5
Chr6:13434156-13438338	4183	5645	5751	2789.16	2678.34
Chr6:17320147-17322325	2179	8080	8970	3992.28	4177.48
Chr6:17673998-17677275	3278	8734	9993	4315.41	4653.91
Chr6:18069265-18071348	2084	8028	8048	3966.58	3748.09
Chr6:19709920-19715677	5758	21992	21816	10866.1	10160.1
Chr6:20383589-20385536	1948	8648	8374	4272.92	3899.91
Chr6:21225648-21227449	1802	12728	13559	6288.82	6314.65
Chr6:27799303-27806512	7210	65115	69406	32172.9	32323.5
Chr6:27806630-27809096	2467	43843	47357	21662.5	22054.9
Chr6:28515521-28519245	3725	40263	43457	19893.7	20238.7
Chr6:29372555-29380618	8064	239188	257689	118181	120010
Chr7:864861-866126	1266	4254	4704	2101.87	2190.73
Chr7:996428-1000991	4564	13950	14861	6892.61	6921.02
Chr7:4348717-4353086	4370	160150	171141	79129.1	79703.2
Chr7:4372306-4376897	4592	70049	74399	34610.8	34648.9
Chr7:6414208-6415841	1634	8167	7708	4035.26	3589.74
Chr7:6988035-6988932	898	5105	5565	2522.35	2591.71
Chr7:8295276-8300948	5673	62364	66253	30813.7	30855.1
Chr7:15676149-15678935	2787	23479	23957	11600.8	11157.2
Chr7:15713035-15717883	4849	33304	35289	16455.3	16434.7
Chr7:15986539-15990993	4455	42495	44284	20996.5	20623.8
Chr7:16064531-16066301	1771	4387	4321	2167.59	2012.36
Chr7:18498538-18501295	2758	6141	6407	3034.23	2983.85
Chr7:19998919-20004643	5725	27779	29229	13725.4	13612.4
Chr7:23520036-23521984	1949	11359	12752	5612.41	5938.82
Chr7:27168790-27170489	1700	10326	10932	5102.01	5091.22
Chr7:28997880-28999201	1322	4914	5181	2427.98	2412.88
Chr8:5775724-5778224	2501	10691	11743	5282.36	5468.91
Chr8:5778349-5780487	2139	27402	30700	13539.2	14297.5
Chr8:7666904-7671942	5039	27089	28536	13384.5	13289.7
Chr8:7765914-7767565	1652	10041	11277	4961.19	5251.89
Chr8:7767691-7772582	4892	50567	53633	24984.8	24977.8
Chr8:7989823-7992612	2790	8171	9022	4037.24	4201.7
Chr8:8446510-8449684	3175	5260	5170	2598.93	2407.76
Chr8:9442911-9449183	6273	41629	43612	20568.6	20310.8
Chr8:10491721-10499795	8075	96617	103145	47737.8	48036.4
Chr8:15383732-15386857	3126	4612	5173	2278.76	2409.15
Chr8:15452050-15466199	14150	362337	379445	179028	176714
Chr8:16975293-16979494	4202	20760	21650	10257.4	10082.8
Chr8:20979660-20986590	6931	55691	57924	27516.6	26976.2
Chr8:21691320-21695444	4125	54903	56486	27127.2	26306.5
Chr8:27144980-27147526	2547	24077	24610	11896.3	11461.3
Chr9:1292302-1298579	6278	32420	34405	16018.5	16023
Chr9:3227716-3231468	3753	28589	30363	14125.6	14140.6
Chr9:3512136-3519406	7271	13851	14403	6843.69	6707.72
Chr9:12177632-12184663	7032	12668	14221	6259.18	6622.96
Chr9:12418936-12426852	7917	29988	31641	14816.9	14735.7
Chr9:12621273-12623420	2148	12137	13656	5996.81	6359.83
Chr9:15227970-15231916	3947	38764	40419	19153	18823.8
Chr9:16722326-16726841	4516	100536	105521	49674.2	49142.9
Chr9:17428605-17430531	1927	5391	5557	2663.66	2587.99

Bill-siRNA clusters in sperm

Region	Length	sperm-R1_reads	sperm-R2_reads	sperm-R1_RPTM	sperm-R2_RPTM
Chr1:5832452-5832480	29	5350	6150	2640.2	2560.06
Chr1:13389750-13392539	2790	98563	117174	48640.3	48776.1
Chr1:22638789-22639134	346	20194	25041	9965.63	10423.8
Chr1:22639312-22639986	675	25265	31011	12468.1	12909
Chr10:4833469-4833493	25	22021	25793	10867.3	10736.9
Chr10:4833653-4833734	82	6671	8866	3292.1	3690.65

Chr10:5733760-5733795	36	8784	11139	4334.86	4636.84
Chr10:5863937-5864726	790	6078	7129	2999.46	2967.59
Chr10:11842695-11843341	647	5847	6282	2885.46	2615.01
Chr10:11861538-11861955	418	8629	11399	4258.37	4745.07
Chr10:11872745-11872979	235	4313	6022	2128.44	2506.78
Chr10:12917830-12918270	441	8877	13950	4380.75	5806.97
Chr11:2525476-2525850	375	4500	6436	2220.73	2679.12
Chr11:6404058-6404508	451	8499	7672	4194.21	3193.63
Chr11:6404612-6404765	154	6847	7216	3378.96	3003.81
Chr11:9962541-9962695	155	4005	5932	1976.45	2469.32
Chr11:12039479-12045573	6095	7935	9516	3915.88	3961.23
Chr11:12082057-12091313	9257	11506	13319	5678.15	5544.31
Chr11:12091434-12098260	6827	7798	9409	3848.27	3916.69
Chr11:12098377-12105542	7166	8366	10201	4128.58	4246.37
Chr11:12108857-12112490	3634	4192	5157	2068.73	2146.71
Chr11:12112628-12116000	3373	5205	5936	2568.64	2470.98
Chr11:12117578-12122235	4658	6989	8787	3449.04	3657.77
Chr11:12152601-12157037	4437	4827	6385	2382.1	2657.89
Chr11:12159598-12163159	3562	4296	5263	2120.05	2190.83
Chr11:12277524-12280220	2697	4484	6251	2212.83	2602.11
Chr11:25567052-25567443	392	17384	20785	8578.91	8652.18
Chr12:9355716-9356887	1172	21288	25103	10505.5	10449.6
Chr12:10451711-10452799	1089	10719	12113	5289.77	5042.28
Chr12:10452944-10454043	1100	7906	10469	3901.57	4357.93
Chr12:22728962-22729161	200	6610	7011	3262	2918.47
Chr12:22757840-22757936	97	5555	7987	2741.36	3324.75
Chr2:6004101-6004463	363	7493	9655	3697.76	4019.09
Chr2:6858743-6859225	483	6363	8911	3140.11	3709.39
Chr2:7682570-7682849	280	4809	4883	2373.22	2032.65
Chr2:11111727-11114974	3248	60839	62512	30023.7	26021.9
Chr3:7365210-7366325	1116	8620	9321	4253.93	3880.06
Chr3:7366446-7367619	1174	6664	9251	3288.65	3850.92
Chr3:28646237-28646388	152	3703	6759	1827.41	2813.57
Chr4:12225786-12225902	117	4138	4963	2042.08	2065.95
Chr5:7442444-7444284	1841	4684	4898	2311.53	2038.89
Chr5:8700746-8702511	1766	18620	20466	9188.87	8519.39
Chr5:9162850-9164714	1865	8732	8303	4309.2	3456.29
Chr5:10952586-10952876	291	4086	6353	2016.42	2644.57
Chr5:19929254-19929538	285	4933	4001	2434.41	1665.5
Chr5:22687899-22688158	260	12326	12537	6082.82	5218.78
Chr6:7908551-7909362	812	6120	8690	3020.19	3617.39
Chr6:12837394-12838523	1130	63572	73822	31372.5	30729.9
Chr6:13330470-13330579	110	6030	6920	2975.77	2880.59
Chr6:25366430-25367236	807	5558	6135	2742.84	2553.82
Chr6:25428084-25428691	608	10874	12182	5366.26	5071.01
Chr6:25516864-25517510	647	5557	9093	2742.35	3785.15
Chr7:20909662-20911024	1363	28409	35936	14019.7	14959.1
Chr7:26333237-26333320	84	11365	12283	5608.57	5113.05
Chr8:808546-809249	704	10868	11481	5363.3	4779.2
Chr8:812059-812362	304	11407	14497	5629.3	6034.67
Chr8:7856645-7857710	1066	5883	3734	2903.23	1554.35
Chr8:17680393-17681092	700	41804	64556	20630.1	26872.8
Chr8:17681885-17684563	2679	73033	91687	36041.4	38166.6
Chr8:23993800-23994189	390	4826	6967	2381.61	2900.16
Chr9:309524-310691	1168	4016	5861	1981.88	2439.76
Chr9:311098-312110	1013	8130	6558	4012.11	2729.9
Chr9:3963462-3964322	861	6532	7057	3223.51	2937.62
Chr9:10835754-10836624	871	19085	18591	9418.35	7738.88
Chr9:10836802-10837433	632	9590	17086	4732.62	7112.4

Bill-siRNA clusters in vegetative cell

Region	Length	vegetative cell-R1_reads	vegetative cell-R2_reads	vegetative cell-R1_RPTM	vegetative cell-R2_RPTM
Chr1:13389841-13392431	2591	42874	31025	29271	27469.8
Chr1:22638784-22639174	391	4603	3124	3142.56	2766.01
Chr1:22639283-22640026	744	10467	5700	7146.04	5046.82
Chr11:6403919-6404765	847	10662	7119	7279.17	6303.22
Chr11:25567052-25567581	530	6572	3772	4486.84	3339.76
Chr12:4730782-4736293	5512	3647	2059	2489.88	1823.05
Chr12:9355710-9356884	1175	4451	3148	3038.79	2787.26
Chr12:10451673-10454043	2371	3558	2186	2429.12	1935.5
Chr2:6003909-6004485	577	5938	4642	4054	4110.06
Chr2:11111727-11114965	3239	14248	10348	9727.41	9162.2
Chr5:26275526-26276139	614	3588	2654	2449.6	2349.87
Chr6:12837392-12838522	1131	20289	13776	13851.7	12197.4
Chr6:13330468-13330579	112	4667	3003	3186.26	2658.88
Chr6:25428029-25428706	678	4555	3617	3109.79	3202.52
Chr7:20909511-20911196	1686	7313	5629	4992.74	4983.96
Chr8:15455302-15462222	6921	5897	3758	4026	3327.36
Chr8:17680076-17680964	889	4615	3205	3150.76	2837.73
Chr8:17681903-17684574	2672	40693	31218	27782	27640.7

Bill-siRNA clusters in Nipponbare embryo

Region	Length	embryo_reads	embryo_RPTM
Chr4:19715479-19715586	108	6346	3121.91
Chr7:136115-139833	3719	15007	7382.67
Chr8:20239410-20241532	2123	15660	7703.92
Chr8:20243047-20245138	2092	16741	8235.71
Chr12:9355653-9356898	1246	7987	3929.19

Bill-siRNA clusters in Nipponbare endosperm

Region	Length	endosperm_reads	endosperm_RPTM
Chr1:3699712-3703446	3735	3088	2185.19
Chr1:4260932-4268040	7109	12279	8689.1
Chr1:4776252-4781931	5680	3092	2188.02
Chr1:5577127-5590843	13717	38328	27122.4
Chr1:5594143-5597892	3750	8181	5789.19
Chr1:7151897-7157057	5161	3562	2520.61
Chr1:7159014-7167085	8072	34987	24758.2
Chr1:7170133-7174133	4001	4886	3457.52
Chr1:7536240-7540264	4025	5069	3587.02
Chr1:7541343-7547109	5767	9135	6464.28
Chr1:8599429-8604337	4909	51444	36403.8
Chr1:13950072-13957986	7915	30784	21784
Chr1:14130361-14130667	307	4090	2894.24
Chr1:18516522-18519391	2870	4642	3284.86
Chr1:20862907-20865552	2646	3502	2478.15
Chr1:22833158-22841076	7919	15182	10743.4
Chr1:23310763-23312834	2072	4489	3176.59
Chr1:23382930-23389754	6825	13634	9647.95
Chr1:23395248-23400986	5739	20031	14174.7
Chr1:26558643-26564521	5879	6055	4284.75
Chr1:26924199-26932111	7913	47067	33306.4
Chr1:32308796-32314030	5235	23594	16696
Chr1:37168571-37173029	4459	11684	8268.05
Chr1:38341385-38348660	7276	45696	32336.3
Chr1:40437030-40441825	4796	5386	3811.34
Chr1:40539621-40546418	6798	25431	17996
Chr1:42568754-42574755	6002	8000	5661.11
Chr2:6772541-6780956	8416	24542	17366.9
Chr2:6781277-6783956	2680	3012	2131.41
Chr2:7635652-7642634	6983	4968	3515.55
Chr2:8587388-8593367	5980	5260	3722.18
Chr2:9855067-9859230	4164	7819	5533.03
Chr2:9860949-9863940	2992	8209	5809.01
Chr2:11488559-11501617	13059	55429	39223.7
Chr2:11897062-11902896	5835	3192	2258.78
Chr2:14456000-14459752	3753	3566	2523.44
Chr2:14923617-14926067	2451	5082	3596.22
Chr2:17351656-17362573	10918	72826	51534.5
Chr2:29053895-29058370	4476	8298	5871.99
Chr2:30333408-30334387	980	3882	2747.05
Chr2:33157188-33162071	4884	11246	7958.11
Chr2:34010148-34015864	5717	3017	2134.95
Chr3:6510677-6511004	328	24331	17217.6
Chr3:9280806-9283054	2249	4789	3388.88
Chr3:9283171-9285763	2593	4241	3001.1
Chr3:12908213-12909522	1310	4852	3433.46
Chr3:12909670-12911722	2053	5808	4109.97
Chr3:12967070-12976657	9588	48620	34405.4
Chr3:13250042-13256116	6075	9695	6860.56
Chr3:16602800-16605557	2758	3620	2561.65
Chr3:16864791-16871680	6890	4489	3176.59
Chr3:17902445-17908218	5774	4337	3069.03
Chr3:17908331-17911816	3486	4759	3367.65
Chr3:25176521-25180837	4317	3688	2609.77
Chr3:27070415-27073544	3130	6198	4385.95
Chr3:32609748-32614193	4446	5019	3551.64
Chr3:36125868-36131839	5972	8391	5937.8
Chr4:1745684-1747360	1677	4161	2944.48
Chr4:4588040-4593805	5766	49849	35275.1
Chr4:4600073-4608106	8034	27628	19550.6
Chr4:5474890-5477996	3107	4726	3344.3
Chr4:5491394-5497324	5931	7169	5073.06
Chr4:11672651-11677517	4867	11815	8360.75
Chr4:12622611-12630837	8227	9950	7041.01
Chr4:16872735-16879711	6977	3043	2153.34
Chr4:16880431-16887050	6620	4942	3497.15
Chr4:18421939-18426415	4477	11786	8340.23
Chr4:18427336-18433949	6614	18601	13162.8
Chr4:19448581-19453108	4528	9656	6832.96
Chr4:19715121-19715243	123	2985	2112.3
Chr4:19715477-19715584	108	16756	11857.2
Chr4:21858330-21863964	5635	4352	3079.64
Chr4:26769647-26774571	4925	38271	27082

Chr4:29781853-29785951	4099	5801	4105.01
Chr4:31205978-31211720	5743	4538	3211.26
Chr4:35113702-35118644	4943	19380	13714
Chr5:543044-548100	5057	14696	10399.5
Chr5:1510257-1513811	3555	6169	4365.42
Chr5:5538679-5543298	4620	10831	7664.44
Chr5:7102550-7109275	6726	6013	4255.03
Chr5:7113669-7118855	5187	6215	4397.97
Chr5:7190890-7197442	6553	8407	5949.12
Chr5:10973694-10977337	3644	4188	2963.59
Chr5:13579598-13584409	4812	6180	4373.21
Chr5:13585373-13587312	1940	3393	2401.02
Chr5:15192220-15196447	4228	7004	4956.3
Chr5:18200643-18207204	6562	4536	3209.85
Chr5:18226942-18233386	6445	40403	28590.7
Chr5:18244531-18252770	8240	25278	17887.7
Chr5:19267295-19273494	6200	9029	6389.27
Chr5:19273621-19279216	5596	17550	12419.1
Chr5:19704862-19710624	5763	6415	4539.5
Chr5:20042720-20045331	2612	3267	2311.86
Chr5:20466930-20470587	3658	4773	3377.56
Chr5:22114552-22116524	1973	7032	4976.12
Chr5:23709449-23716203	6755	10910	7720.34
Chr5:24463339-24468826	5488	7231	5116.94
Chr5:25076795-25080541	3747	2945	2084
Chr5:26923669-26926976	3308	3013	2132.12
Chr5:28641134-28646017	4884	8580	6071.54
Chr6:518788-521036	2249	2895	2048.61
Chr6:2598764-2603873	5110	7149	5058.91
Chr6:2604838-2613043	8206	49217	34827.9
Chr6:3724722-3731040	6319	6602	4671.83
Chr6:3748674-3758502	9829	30767	21771.9
Chr6:4974281-4975759	1479	3650	2582.88
Chr6:6214351-6218208	3858	17152	12137.4
Chr6:6420437-6435842	15406	68726	48633.2
Chr6:12657135-12657443	309	3831	2710.96
Chr6:12764609-12767052	2444	4785	3386.05
Chr6:13329784-13329990	207	5731	4055.48
Chr6:19501840-19512759	10920	13463	9526.94
Chr6:19597247-19606841	9595	8372	5924.35
Chr6:20991318-20996023	4706	12466	8821.42
Chr6:21837740-21841292	3553	5536	3917.49
Chr6:25785609-25794475	8867	50216	35534.8
Chr6:30880570-30885135	4566	4171	2951.56
Chr6:30926407-30932387	5981	19260	13629.1
Chr6:30934501-30939773	5273	8385	5933.55
Chr7:136275-139649	3375	4841	3425.68
Chr7:1041444-1046253	4810	8510	6022.01
Chr7:1299133-1302117	2985	10795	7638.96
Chr7:4703562-4710912	7351	40300	28517.8
Chr7:4988775-4999808	11034	79906	56544.6
Chr7:6774867-6785518	10652	9439	6679.4
Chr7:7211617-7219281	7665	34415	24353.4
Chr7:8047974-8058021	10048	27939	19770.7
Chr7:9122576-9122797	222	4339	3070.44
Chr7:11005330-11016894	11565	40357	28558.2
Chr7:18661509-18666987	5479	3239	2292.04
Chr7:21685635-21694444	8810	47235	33425.3
Chr7:21708832-21711565	2734	3080	2179.53
Chr7:21714059-21720752	6694	79544	56288.4
Chr7:21834761-21840963	6203	12766	9033.72
Chr7:22527588-22531198	3611	6303	4460.25
Chr7:28193106-28200419	7314	29072	20572.5
Chr8:818500-822923	4424	4599	3254.43
Chr8:1614778-1618869	4092	5696	4030.71
Chr8:1627895-1633355	5461	22629	16013.2
Chr8:4308030-4312675	4646	7468	5284.65
Chr8:4366038-4369562	3525	2865	2027.39
Chr8:4372421-4379910	7490	11311	8004.1
Chr8:4380033-4383161	3129	4513	3193.57
Chr8:5165968-5170308	4341	3965	2805.79
Chr8:5172680-5177696	5017	8719	6169.9
Chr8:7662701-7673315	10615	37907	26824.5
Chr8:7694562-7701713	7152	6426	4547.29
Chr8:8702584-8703105	522	100427	71066
Chr8:9051839-9057509	5671	4262	3015.96
Chr8:10866521-10873299	6779	34454	24381
Chr8:10882220-10889865	7646	8612	6094.19
Chr8:10890038-10894831	4794	22744	16094.5
Chr8:14965911-14970477	4567	11771	8329.62
Chr8:17184421-17189346	4926	8890	6290.91
Chr8:17666819-17669871	3053	5229	3700.24
Chr8:17701283-17712303	11021	14545	10292.6
Chr8:17721765-17726019	4255	5375	3803.56
Chr8:20987966-20993398	5433	9819	6948.31
Chr8:21004031-21009540	5510	9286	6571.13
Chr8:21423862-21432609	8748	22904	16207.8
Chr8:22037072-22047584	10513	9289	6573.26

Chr8:22080687-22089312	8626	26032	18421.3
Chr8:22329945-22331794	1850	3672	2598.45
Chr8:22467034-22473077	6044	41086	29074
Chr8:22503701-22508998	5298	44804	31705
Chr8:22821605-22829085	7481	4504	3187.2
Chr9:1014-37759	36746	30953	21903.5
Chr9:1070370-1078318	7949	6533	4623
Chr9:1183588-1192199	8612	6448	4562.85
Chr9:1195496-1201177	5682	15076	10668.4
Chr9:1228561-1235121	6561	3871	2739.27
Chr9:4617635-4619977	2343	2974	2104.52
Chr9:5765708-5768851	3144	3211	2272.23
Chr9:5769277-5780934	11658	37980	26876.1
Chr9:6465808-6470439	4632	3454	2444.18
Chr9:6544064-6554889	10826	32539	23025.9
Chr9:8572142-8577814	5673	4159	2943.07
Chr9:12921772-12925470	3699	2842	2011.11
Chr9:14441072-14448242	7171	18801	13304.3
Chr9:15991281-15995684	4404	11023	7800.3
Chr9:16533117-16542149	9033	98950	70020.9
Chr9:17592956-17603158	10203	36464	25803.3
Chr9:18114403-18120398	5996	3440	2434.28
Chr9:18963880-18967002	3123	5266	3726.43
Chr9:19226656-19231907	5252	16684	11806.2
Chr9:19305089-19309195	4107	3712	2626.76
Chr9:19532886-19539445	6560	41387	29287
Chr9:20616681-20622422	5742	24416	17277.7
Chr9:21044077-21044386	310	8398	5942.75
Chr9:21045164-21045661	498	3746	2650.81
Chr9:21245909-21252086	6178	10266	7264.62
Chr9:22069975-22075683	5709	4377	3097.33
Chr10:2397960-2423525	25566	195271	138181
Chr10:2727541-2733712	6172	33564	23751.2
Chr10:2901761-2906633	4873	3509	2483.1
Chr10:4833918-4834134	217	3160	2236.14
Chr10:6928550-6928893	344	5466	3867.95
Chr10:7297355-7304894	7540	14379	10175.1
Chr10:12012669-12016454	3786	7303	5167.89
Chr10:13749032-13758836	9805	24783	17537.4
Chr11:2911079-2921919	10841	9322	6596.61
Chr11:3993091-4002573	9483	129021	91300.3
Chr11:4894957-4906014	11058	22607	15997.6
Chr11:10854855-10859224	4370	4057	2870.89
Chr11:11684803-11691018	6216	13485	9542.51
Chr11:12180073-12187686	7614	3512	2485.23
Chr11:12277522-12282433	4912	8660	6128.15
Chr11:15737744-15740540	2797	5327	3769.59
Chr11:16131029-16133470	2442	3294	2330.96
Chr11:17860045-17865726	5682	8087	5722.67
Chr11:18312134-18317187	5054	19221	13601.5
Chr11:19099035-19109418	10384	4667	3302.55
Chr11:21704245-21704368	124	5717	4045.57
Chr11:25021244-25028096	6853	4112	2909.81
Chr11:26689012-26694756	5745	2860	2023.85
Chr11:28914356-28918364	4009	10390	7352.37
Chr12:1813844-1817700	3857	4483	3172.34
Chr12:2869401-2882809	13409	42183	29850.3
Chr12:2893186-2897946	4761	3276	2318.22
Chr12:3095465-3099872	4408	10013	7085.59
Chr12:5575371-5579072	3702	2886	2042.25
Chr12:6399784-6403003	3220	3920	2773.94
Chr12:7743899-7747741	3843	2834	2005.45
Chr12:8656069-8658444	2376	15668	11087.3
Chr12:9355605-9356892	1288	4234	2996.14
Chr12:10112258-10125312	13055	45254	32023.5
Chr12:10176952-10184213	7262	27653	19568.3
Chr12:10337265-10341384	4120	3520	2490.89
Chr12:18660081-18660284	204	2867	2028.8
Chr12:19319837-19325505	5669	12898	9127.12
Chr12:19327241-19329934	2694	3564	2522.02
Chr12:19362489-19362966	478	4788	3388.17
Chr12:19373296-19377539	4244	28017	19825.9
Chr12:19377644-19380316	2673	24063	17027.9
Chr12:19389554-19392520	2967	4816	3407.99
Chr12:19401064-19414731	13668	96285	68135
Chr12:20876091-20884262	8172	9886	6995.72
Chr12:24879829-24882413	2585	3836	2714.5

Bill-siRNA clusters in TP309 seedling

Region	Length	TP309 seedling-R1_reads	TP309 seedling-R2_reads	TP309 seedling-R1_RPTM	TP309 seedling-R2_RPTM
Chr10:4833387-4833506	120	6283	5580	4548.56	4392.75
Chr12:9355709-9356884	1176	4105	4191	2971.8	3299.29
Chr2:1553148-1553255	108	14825	15457	10732.5	12168.2

Chr2:6001244-6001995	752	5985	24	4332.82	18.8935
Chr2:8522078-8522111	34	6253	7836	4526.84	6168.74
Chr3:30515079-30515181	103	2716	3188	1966.24	2509.69
Chr4:19715121-19715307	187	4394	5097	3181.02	4012.52
Chr4:19715477-19715702	226	4102	5200	2969.63	4093.6
Chr6:13330447-13330641	195	2941	3135	2129.13	2467.97
Chr6:21165722-21165845	124	6153	3246	4454.45	2555.35
Chr7:136223-139657	3435	8894	9437	6438.79	7429.1
Chr8:9193708-9194236	529	3503	3854	2535.99	3033.99

Bill-siRNA clusters in TP309 inflorescence

Region	Length	TP309 inflorescence-R1_reads	TP309 inflorescence-R2_reads	TP309 inflorescence-R1_RPTM	TP309 inflorescence-R2_RPTM
Chr1:2521832-2525868	4037	4068	4307	4557.31	4406.17
Chr1:2555533-2560396	4864	16889	16059	18920.4	16428.8
Chr1:9039407-9044856	5450	12799	8997	14338.5	9204.16
Chr1:9435855-9439656	3802	23905	23598	26780.3	24141.4
Chr1:10921554-10924098	2545	6653	6551	7453.23	6701.84
Chr1:10924269-10926494	2226	13817	15319	15478.9	15671.7
Chr1:11189858-11191482	1625	4329	3463	4849.7	3542.74
Chr1:11194285-11200503	6219	12863	15561	14410.2	15919.3
Chr1:11360853-11365225	4373	22545	24885	25256.7	25458
Chr1:11470340-11475439	5100	26501	28745	29688.6	29406.9
Chr1:12139282-12145475	6194	5761	5565	6453.94	5693.14
Chr1:14333189-14340569	7381	39838	45119	44629.8	46157.9
Chr1:14959401-14964483	5083	5822	6774	6522.28	6929.97
Chr1:20809576-20814772	5197	12859	12792	14405.7	13086.5
Chr1:21008538-21015105	6568	4591	4683	5143.21	4790.83
Chr1:25251600-25254709	3110	2623	3009	2938.5	3078.28
Chr1:25311726-25313682	1957	4825	5967	5405.36	6104.39
Chr1:26580342-26582385	2044	1971	1763	2208.07	1803.59
Chr1:28199280-28206541	7262	40936	38693	45859.8	39583.9
Chr1:28209600-28216678	7079	18496	19053	20720.7	19491.7
Chr1:29052999-29057508	4510	6750	8897	7561.9	9101.86
Chr1:30808064-30812188	4125	4696	5441	5260.84	5566.28
Chr10:706462-709675	3214	6897	6941	7726.58	7100.82
Chr10:3440843-3446855	6013	7091	6580	7943.92	6731.51
Chr10:5924603-5934240	9638	34696	34056	38869.3	34840.2
Chr10:15985585-15991286	5702	15528	18748	17395.7	19179.7
Chr10:16003040-16005183	2144	3782	6529	4236.9	6679.33
Chr10:16005550-16007065	1516	7279	9390	8154.53	9606.21
Chr10:20776661-20779960	3300	7340	9384	8222.87	9600.07
Chr11:2541020-2543933	2914	3082	4910	3452.71	5023.05
Chr11:3230045-3233132	3088	13512	14744	15137.2	15083.5
Chr11:7332520-7335712	3193	3052	3039	3419.1	3108.97
Chr11:7731055-7741761	10707	61583	65288	68990.3	66791.3
Chr11:8910215-8915580	5366	1960	2352	2195.75	2406.16
Chr11:16329892-16336817	6926	44462	50405	49810	51565.6
Chr11:17164428-17170275	5848	11077	9748	12409.4	9972.45
Chr11:20706555-20709432	2878	9473	11030	10612.4	11284
Chr11:22338292-22343006	4715	3721	3101	4168.57	3172.4
Chr11:24387252-24394638	7387	26102	31136	29241.6	31852.9
Chr11:27016567-27019285	2719	4296	5513	4812.73	5639.94
Chr12:4726893-4738687	11795	53586	56898	60031.4	58208.1
Chr12:4898719-4904134	5416	16599	16300	18595.6	16675.3
Chr12:4911981-4917807	5827	17264	16812	19340.5	17199.1
Chr12:5024937-5028201	3265	2398	1787	2686.44	1828.15
Chr12:9061792-9066505	4714	3574	3496	4003.89	3576.5
Chr12:17499964-17504299	4336	19005	22375	21290.9	22890.2
Chr12:17984443-17989007	4565	11734	14537	13145.4	14871.7
Chr12:18392996-18397217	4222	3548	3820	3974.76	3907.96
Chr12:18860689-18866408	5720	16516	17835	18502.6	18245.7
Chr12:24724489-24735566	11078	51876	53574	58115.7	54807.6
Chr2:1553148-1553331	184	13059	9198	14629.8	9409.79
Chr2:3700051-3702962	2912	1816	1958	2034.43	2003.08
Chr2:5900026-5907024	6999	25295	28839	28337.5	29503
Chr2:6759230-6764148	4919	11252	13464	12605.4	13774
Chr2:6795066-6797015	1950	9726	12171	10895.9	12451.2
Chr2:6797248-6798611	1364	4140	5380	4637.97	5503.88
Chr2:7604967-7611993	7027	12270	16746	13745.9	17131.6
Chr2:8343289-8346969	3681	13686	13658	15332.2	13972.5
Chr2:8522078-8522114	37	3281	4911	3675.64	5024.08
Chr2:8525127-8525258	132	1685	2169	1887.67	2218.94
Chr2:13580496-13582726	2231	1895	2670	2122.93	2731.48
Chr2:13582836-13584440	1605	9026	8408	10111.7	8601.6
Chr2:13584554-13586358	1805	8089	7306	9061.96	7474.22
Chr2:19469359-19471202	1844	18687	15022	20934.7	15367.9
Chr2:29633230-29636007	2778	4345	3970	4867.62	4061.41
Chr2:29777060-29783149	6090	5914	8091	6625.34	8277.3
Chr2:33848550-33850734	2185	1638	2343	1835.02	2396.95
Chr3:15609571-15614403	4833	2149	3033	2407.48	3102.84
Chr3:16790294-16793977	3684	1793	2251	2008.66	2302.83
Chr3:19352770-19356619	3850	1653	2309	1851.83	2362.17

Chr3:22402915-22411469	8555	29819	28889	33405.7	29554.2
Chr3:24566448-24574132	7685	25154	30221	28179.6	30916.9
Chr3:24600910-24606911	6002	10296	11782	11534.4	12053.3
Chr3:26123026-26126247	3222	11966	12659	13405.3	12950.5
Chr3:27616647-27619389	2743	1759	2337	1970.58	2390.81
Chr3:27619512-27621777	2266	6852	8278	7676.17	8468.6
Chr3:28510208-28511760	1553	1803	1974	2019.87	2019.45
Chr3:29749941-29755991	6051	3523	2848	3946.75	2913.58
Chr4:11651745-11657119	5375	3520	4442	3943.39	4544.28
Chr4:19681434-19685210	3777	6360	8610	7124.99	8808.25
Chr4:35049575-35053502	3928	20001	25287	22406.8	25869.2
Chr5:661602-664198	2597	2424	2354	2715.56	2408.2
Chr5:1132551-1140459	7909	13864	16924	15531.6	17313.7
Chr5:1680404-1684309	3906	8537	10355	9563.84	10593.4
Chr5:4825918-4831618	5701	9748	9163	10920.5	9373.98
Chr5:10916929-10920211	3283	9925	10031	11118.8	10262
Chr5:10920336-10923004	2669	2396	2768	2684.19	2831.73
Chr5:11242416-11247138	4723	30244	33801	33881.8	34579.3
Chr5:16055425-16058035	2611	6433	6468	7206.77	6616.93
Chr5:25352167-25357744	5578	40804	41812	45712	42774.7
Chr5:26871506-26874206	2701	7366	7569	8251.99	7743.28
Chr6:6078050-6082881	4832	12068	12585	13519.6	12874.8
Chr6:7535134-7539311	4178	18301	20073	20502.3	20535.2
Chr6:11299542-11306993	7452	20105	22132	22523.3	22641.6
Chr6:13424874-13438632	13759	57430	61030	64337.8	62435.2
Chr6:17320172-17323420	3249	1919	2584	2149.82	2643.5
Chr6:19709920-19712027	2108	3220	4041	3607.31	4134.05
Chr6:21224117-21227496	3380	3291	4336	3686.85	4435.84
Chr6:25366408-25367056	649	384	3936	430.188	4026.63
Chr6:27798388-27809478	11091	28375	27040	31788	27662.6
Chr6:28515631-28518627	2997	5120	5812	5735.84	5945.82
Chr6:29376919-29380614	3696	29860	31602	33451.6	32329.6
Chr7:996134-1001319	5186	3115	3802	3489.68	3889.54
Chr7:4346893-4353143	6251	30246	34305	33884	35094.9
Chr7:4372372-4376295	3924	22196	21356	24865.8	21847.7
Chr7:8295232-8301023	5792	13472	13136	15092.4	13438.5
Chr7:15712890-15717884	4995	6565	7538	7354.65	7711.57
Chr7:15986459-15991194	4736	9959	12056	11156.9	12333.6
Chr7:19999640-20006845	7206	7336	8111	8218.38	8297.76
Chr7:23519976-23522197	2222	3520	8777	3943.39	8979.09
Chr7:27168754-27170310	1557	1566	2486	1754.36	2543.24
Chr8:5775694-5780872	5179	10368	11010	11615.1	11263.5
Chr8:7667653-7671657	4005	4239	4141	4748.87	4236.35
Chr8:7762820-7770869	8050	17656	19947	19779.7	20406.3
Chr8:9442951-9449260	6310	8439	5604	9454.06	5733.03
Chr8:10491753-10502270	10518	22145	27207	24808.6	27833.5
Chr8:15452052-15462294	10243	67464	76165	75578.7	77918.7
Chr8:16973609-16979484	5876	7310	9303	8189.26	9517.21
Chr8:20978664-20986591	7928	12987	11991	14549.1	12267.1
Chr8:21691353-21695461	4109	9967	10080	11165.8	10312.1
Chr8:27145002-27147836	2835	5669	6646	6350.88	6799.03
Chr9:1292223-1297850	5628	6319	6102	7079.06	6242.5
Chr9:3227798-3229695	1898	1943	2916	2176.71	2983.14
Chr9:3229897-3231862	1966	2222	3441	2489.27	3520.23
Chr9:9556895-9558464	1570	2689	2822	3012.44	2886.98
Chr9:12420620-12427040	6421	3341	6587	3742.86	6738.67
Chr9:12621912-12623506	1595	2234	2984	2502.71	3052.71
Chr9:15228009-15232300	4292	3975	4498	4453.12	4601.57
Chr9:16723081-16727621	4541	27519	32411	30829	33157.3

Bill-siRNA clusters in TP309 endosperm

Region	Length	TP309 endosperm_reads	TP309 endosperm_RPTM
Chr1:2555817-2559537	3721	1866	2360.25
Chr1:3699714-3703754	4041	2659	3363.29
Chr1:4261876-4267470	5595	5786	7318.53
Chr1:5577343-5580874	3532	6283	7947.17
Chr1:5581730-5589870	8141	18767	23737.8
Chr1:7152190-7157133	4944	3849	4868.48
Chr1:7158856-7166897	8042	23247	29404.4
Chr1:7170230-7174138	3909	3698	4677.49
Chr1:7537357-7540212	2856	3591	4542.14
Chr1:7541440-7547107	5668	7642	9666.13
Chr1:8599564-8604129	4566	30365	38407.7
Chr1:9436219-9439656	3438	2067	2614.48
Chr1:10924271-10926494	2224	1600	2023.79
Chr1:11360931-11363743	2813	1954	2471.55
Chr1:11470747-11475396	4650	2807	3550.49
Chr1:13956842-13958367	1526	2168	2742.24
Chr1:14333331-14335954	2624	1621	2050.35
Chr1:18516962-18519248	2287	4813	6087.81
Chr1:22834155-22840290	6136	12641	15989.2
Chr1:23310769-23311842	1074	3412	4315.73
Chr1:23383832-23389553	5722	8838	11178.9

Chr1:23395273-23399801	4529	13254	16764.6
Chr1:23399986-23400995	1010	1916	2423.49
Chr1:23401097-23403318	2222	1929	2439.93
Chr1:26924287-26931228	6942	28697	36297.9
Chr1:28200251-28206538	6288	4000	5059.48
Chr1:28209635-28215248	5614	2069	2617.01
Chr1:32309674-32313890	4217	16108	20374.5
Chr1:38341440-38345532	4093	26278	33238.2
Chr1:40436957-40441074	4118	4051	5123.98
Chr1:40539348-40547010	7663	22214	28097.8
Chr1:42569168-42574745	5578	7798	9863.45
Chr2:1553148-1553230	83	1679	2123.72
Chr2:5901667-5906692	5026	2648	3349.37
Chr2:6772790-6780961	8172	20509	25941.2
Chr2:6781336-6783447	2112	2169	2743.5
Chr2:8587463-8593392	5930	3229	4084.26
Chr2:9855347-9859240	3894	6607	8356.99
Chr2:9860994-9864595	3602	4521	5718.47
Chr2:11488553-11490907	2355	6692	8464.5
Chr2:11491077-11494505	3429	7017	8875.59
Chr2:11494675-11501541	6867	8793	11122
Chr2:12214657-12217238	2582	1674	2117.39
Chr2:12222936-12226254	3319	3941	4984.85
Chr2:14455844-14459675	3832	2343	2963.59
Chr2:14924396-14926082	1687	2772	3506.22
Chr2:17351657-17362932	11276	53272	67382.1
Chr2:27250593-27255473	4881	2094	2648.64
Chr2:29055270-29056420	1151	2434	3078.69
Chr2:33157187-33161980	4794	8263	10451.6
Chr3:4508546-4513609	5064	2919	3692.15
Chr3:9280904-9283063	2160	2872	3632.7
Chr3:9283221-9285209	1989	1992	2519.62
Chr3:12910246-12911368	1123	2366	2992.68
Chr3:12967060-12976252	9193	36817	46568.7
Chr3:13250645-13256185	5541	8621	10904.4
Chr3:16865584-16870045	4462	5656	7154.1
Chr3:17536623-17539556	2934	3131	3960.3
Chr3:17905920-17908217	2298	2129	2692.91
Chr3:20976158-20978551	2394	2613	3305.1
Chr3:22406105-22410755	4651	2713	3431.59
Chr3:24569576-24573183	3608	2173	2748.56
Chr3:25178120-25180813	2694	2408	3045.8
Chr3:27070389-27071836	1448	2387	3019.24
Chr3:32610068-32614223	4156	3462	4378.98
Chr3:36126102-36131813	5712	7215	9126.03
Chr4:1745938-1747341	1404	2484	3141.93
Chr4:4588052-4594154	6103	37539	47481.9
Chr4:4600166-4607239	7074	18691	23641.7
Chr4:5474860-5477069	2210	1828	2312.18
Chr4:5491378-5496958	5581	5730	7247.7
Chr4:11672863-11674397	1535	4650	5881.64
Chr4:12622421-12627621	5201	7132	9021.05
Chr4:12628376-12630742	2367	5484	6936.54
Chr4:18422338-18426438	4101	9240	11687.4
Chr4:18427813-18433505	5693	17505	22141.5
Chr4:19448557-19452694	4138	7317	9255.05
Chr4:19715123-19715247	125	3173	4013.43
Chr4:19715478-19715592	115	3899	4931.72
Chr4:26769648-26774368	4721	32777	41458.6
Chr4:29781896-29785364	3469	6346	8026.86
Chr4:35050583-35053416	2834	2186	2765
Chr4:35113552-35118575	5024	14294	18080
Chr5:542947-547817	4871	11331	14332.2
Chr5:1510260-1513820	3561	4814	6089.08
Chr5:5540375-5543272	2898	6471	8184.97
Chr5:7102993-7105878	2886	3903	4936.78
Chr5:7111061-7114368	3308	2685	3396.17
Chr5:7114691-7118407	3717	3102	3923.62
Chr5:7191775-7197339	5565	3378	4272.73
Chr5:10975028-10977237	2210	3354	4242.37
Chr5:11242426-11246723	4298	3034	3837.61
Chr5:11485814-11487867	2054	1618	2046.56
Chr5:13579674-13583497	3824	4210	5325.1
Chr5:13585420-13587791	2372	3005	3800.93
Chr5:13705694-13708544	2851	2270	2871.25
Chr5:15193335-15196279	2945	4417	5586.93
Chr5:18201880-18205972	4093	2271	2872.52
Chr5:18227558-18229750	2193	8105	10251.8
Chr5:18229870-18231749	1880	9325	11794.9
Chr5:18231870-18233377	1508	3285	4155.09
Chr5:18244456-18252742	8287	25767	32591.9
Chr5:19267669-19272364	4696	6124	7746.06
Chr5:19273592-19280592	7001	14815	18739
Chr5:19706371-19710576	4206	3562	4505.46
Chr5:20468280-20470120	1841	2251	2847.22
Chr5:22115721-22116500	780	3430	4338.5
Chr5:23709442-23714420	4979	7268	9193.07
Chr5:24463732-24468729	4998	4414	5583.13

Chr5:25352224-25357093	4870	4118	5208.73
Chr5:26871607-26874206	2600	1777	2247.67
Chr5:28636425-28640359	3935	2137	2703.03
Chr5:28641249-28645794	4546	6325	8000.3
Chr6:518901-520279	1379	2689	3401.23
Chr6:2599635-2602223	2589	5853	7403.28
Chr6:2605685-2613039	7355	46291	58552.1
Chr6:3724856-3731384	6529	2581	3264.63
Chr6:3749428-3756160	6733	16190	20478.2
Chr6:4972341-4975523	3183	2623	3317.75
Chr6:6214384-6218259	3876	10848	13721.3
Chr6:6420346-6424513	4168	27213	34420.9
Chr6:6424616-6435985	11370	27408	34667.5
Chr6:7535537-7538954	3418	2156	2727.06
Chr6:11301676-11304945	3270	1986	2512.03
Chr6:12764862-12767057	2196	4404	5570.48
Chr6:13330467-13330603	137	3099	3919.83
Chr6:13427980-13432318	4339	4528	5727.33
Chr6:19501897-19503998	2102	2681	3391.11
Chr6:19504583-19512740	8158	7337	9280.34
Chr6:19597247-19601144	3898	3225	4079.2
Chr6:19601278-19608176	6899	3958	5006.35
Chr6:20991321-20996172	4852	12810	16203
Chr6:21165719-21165845	127	1877	2374.16
Chr6:21423301-21426899	3599	1859	2351.39
Chr6:21837795-21841265	3471	3240	4098.18
Chr6:25786231-25794475	8245	31730	40134.3
Chr6:27799534-27809084	9551	2813	3558.08
Chr6:29376922-29380068	3147	2704	3420.21
Chr6:30926664-30929077	2414	5413	6846.74
Chr6:30934342-30939003	4662	7370	9322.08
Chr7:1043841-1045960	2120	5362	6782.23
Chr7:1299213-1300476	1264	2048	2590.45
Chr7:1300579-1302134	1556	3749	4741.99
Chr7:4348719-4353078	4360	3194	4039.99
Chr7:4373503-4376249	2747	1909	2414.63
Chr7:4702318-4703457	1140	2146	2714.41
Chr7:4703562-4711302	7741	40806	51614.2
Chr7:4988786-4999548	10763	69592	88024.8
Chr7:6774396-6785581	11186	12889	16302.9
Chr7:7212409-7219225	6817	27747	35096.3
Chr7:8048308-8058185	9878	25415	32146.6
Chr7:10096589-10098596	2008	1779	2250.2
Chr7:11006183-11014929	8747	18885	23887.1
Chr7:18661540-18667035	5496	2571	3251.98
Chr7:21685950-21694147	8198	35584	45009.1
Chr7:21709023-21711343	2321	2003	2533.53
Chr7:21714111-21720595	6485	64007	80960.5
Chr7:21834781-21840621	5841	9459	11964.4
Chr7:22527006-22529808	2803	3831	4845.71
Chr7:28193302-28200487	7186	22522	28487.4
Chr8:818532-823346	4815	5344	6759.46
Chr8:1614766-1618337	3572	4663	5898.08
Chr8:1627995-1632953	4959	13422	16977.1
Chr8:4307989-4312922	4934	8212	10387.1
Chr8:4365993-4370070	4078	3332	4214.54
Chr8:4372430-4379913	7484	13898	17579.1
Chr8:4380025-4383115	3091	5332	6744.28
Chr8:5165785-5170277	4493	3256	4118.41
Chr8:5173938-5177687	3750	6145	7772.62
Chr8:7662655-7666363	3709	7721	9766.05
Chr8:7667556-7673470	5915	27582	34887.6
Chr8:7695227-7698695	3469	1979	2503.18
Chr8:7698802-7701224	2423	4334	5481.94
Chr8:7764346-7769796	5451	1909	2414.63
Chr8:8702817-8702918	102	15459	19553.6
Chr8:9145444-9150968	5525	5546	7014.96
Chr8:9193386-9194236	851	1732	2190.75
Chr8:10492153-10499819	7667	2312	2924.38
Chr8:10867556-10873271	5716	24643	31170.2
Chr8:10883033-10889820	6788	6266	7925.67
Chr8:10890038-10895268	5231	18086	22876.4
Chr8:14966160-14970456	4297	4242	5365.57
Chr8:15452407-15462092	9686	6503	8225.44
Chr8:17184322-17189325	5004	7222	9134.88
Chr8:17666814-17670168	3355	3993	5050.62
Chr8:17700636-17711324	10689	23988	30341.7
Chr8:17721388-17725886	4499	5666	7166.75
Chr8:20989285-20992609	3325	6568	8307.66
Chr8:21004088-21007118	3031	2732	3455.62
Chr8:21007238-21008907	1670	4374	5532.54
Chr8:21424114-21432407	8294	23747	30036.8
Chr8:21718482-21722308	3827	2504	3167.23
Chr8:22036937-22047604	10668	10979	13887
Chr8:22081006-22088810	7805	24586	31098.1
Chr8:22467052-22472178	5127	26020	32911.9
Chr8:22503692-22508451	4760	27280	34505.6
Chr9:1074972-1077703	2732	2551	3226.68

Chr9:1183551-1187227	3677	1834	2319.77
Chr9:1187645-1193903	6259	2976	3764.25
Chr9:1196074-1200878	4805	16261	20568
Chr9:5765838-5768762	2925	3134	3964.1
Chr9:5769297-5781150	11854	39503	49966.1
Chr9:6544463-6554851	10389	21176	26784.9
Chr9:14440984-14446995	6012	8904	11262.4
Chr9:15991312-15994476	3165	7222	9134.88
Chr9:16533266-16542020	8755	58894	74493.2
Chr9:16723088-16726859	3772	2644	3344.31
Chr9:17595818-17598076	2259	12418	15707.1
Chr9:17598267-17600109	1843	7323	9262.64
Chr9:17600222-17603773	3552	7671	9702.81
Chr9:18963885-18967011	3127	4445	5622.34
Chr9:19227589-19232609	5021	9684	12249
Chr9:19305108-19309221	4114	3456	4371.39
Chr9:19533321-19539840	6520	25659	32455.3
Chr9:20615578-20619926	4349	14136	17880.2
Chr9:20620444-20622430	1987	3708	4690.13
Chr9:21044080-21044115	36	2052	2595.51
Chr9:21045372-21045594	223	2078	2628.4
Chr9:21246679-21252218	5540	10241	12953.5
Chr9:21325122-21328310	3189	1962	2481.67
Chr9:22069981-22075731	5751	4234	5355.46
Chr10:2401007-2401784	778	1812	2291.94
Chr10:2404814-2405822	1009	1823	2305.86
Chr10:2406276-2413314	7039	36033	45577
Chr10:2414426-2414672	247	2349	2971.18
Chr10:2414861-2416625	1765	3616	4573.77
Chr10:2417173-2418145	973	2900	3668.12
Chr10:2727526-2733192	5667	15429	19515.7
Chr10:2901998-2905947	3950	2514	3179.88
Chr10:5925634-5933475	7842	3649	4615.51
Chr10:7297363-7304913	7551	11898	15049.4
Chr10:12013970-12014975	1006	1830	2314.71
Chr10:12015089-12016218	1130	1683	2128.77
Chr10:13749172-13754341	5170	12363	15637.6
Chr10:13754623-13756981	2359	1697	2146.48
Chr10:15987275-15991248	3974	1865	2358.98
Chr11:2911331-2921755	10425	13381	16925.2
Chr11:2923313-2924804	1492	1649	2085.77
Chr11:3993089-4002847	9759	104083	131651
Chr11:4894960-4906153	11194	10763	13613.8
Chr11:7731165-7734531	3367	2720	3440.44
Chr11:7738737-7741790	3054	1834	2319.77
Chr11:9449531-9451996	2466	2484	3141.93
Chr11:11687676-11688950	1275	5122	6478.66
Chr11:11689057-11689811	755	2156	2727.06
Chr11:15737757-15739855	2099	2607	3297.51
Chr11:16130605-16133551	2947	1664	2104.74
Chr11:16330799-16336649	5851	4332	5479.41
Chr11:17860076-17865726	5651	7842	9919.1
Chr11:18312138-18317185	5048	8200	10371.9
Chr11:19102806-19106758	3953	4257	5384.55
Chr11:24387678-24394624	6947	3471	4390.36
Chr11:25023874-25025610	1737	1826	2309.65
Chr11:28915041-28917571	2531	6399	8093.9
Chr12:2869634-2882792	13159	36676	46390.3
Chr12:2893885-2897910	4026	3040	3845.2
Chr12:3094190-3099633	5444	6601	8349.4
Chr12:4728657-4736294	7638	4821	6097.93
Chr12:4912415-4916843	4429	2027	2563.89
Chr12:5575307-5579337	4031	3225	4079.2
Chr12:6399805-6403120	3316	1998	2527.21
Chr12:7180675-7184679	4005	2358	2982.56
Chr12:8656081-8658356	2276	12995	16437
Chr12:10112359-10115339	2981	4401	5566.69
Chr12:10115608-10124195	8588	30653	38772
Chr12:10177918-10185087	7170	22550	28522.8
Chr12:10337333-10340366	3034	2672	3379.73
Chr12:13480000-13482762	2763	3487	4410.6
Chr12:17500497-17503825	3329	2042	2582.86
Chr12:19319050-19323890	4841	7768	9825.5
Chr12:19327437-19329763	2327	3450	4363.8
Chr12:19374466-19377539	3074	24336	30781.9
Chr12:19377669-19380258	2590	22818	28861.8
Chr12:19389016-19392455	3440	6647	8407.58
Chr12:19401051-19414790	13740	81001	102456
Chr12:20875170-20883468	8299	5771	7299.56
Chr12:22699696-22701320	1625	1764	2231.23
Chr12:24728433-24734446	6014	4977	6295.25
Chr12:24879830-24881083	1254	3226	4080.47

Bill-siRNA clusters in TP309 seed coat

Region	Length	TP309 seed coat_reads	TP309 seed coat_RPTM
Chr1:2521761-2525534	3774	10397	6751.76
Chr1:2555533-2560009	4477	41450	26917.4
Chr1:9039052-9045759	6708	41082	26678.4
Chr1:9435827-9439656	3830	56822	36899.9
Chr1:10921606-10924098	2493	12447	8083.02
Chr1:10924269-10926494	2226	38231	24827
Chr1:11187808-11189665	1858	5438	3531.41
Chr1:11189850-11192208	2359	11773	7645.33
Chr1:11194285-11201191	6907	34123	22159.3
Chr1:11360799-11365089	4291	73647	47826
Chr1:11470333-11475439	5107	90314	58649.5
Chr1:12139277-12145610	6334	22602	14677.6
Chr1:14333171-14340725	7555	106793	69350.8
Chr1:14959356-14964153	4798	24458	15882.9
Chr1:19259410-19274571	15162	12069	7837.55
Chr1:20809709-20814697	4989	33411	21696.9
Chr1:21008538-21023337	14800	18602	12080
Chr1:25251585-25254751	3167	6110	3967.8
Chr1:25311726-25313915	2190	16027	10407.9
Chr1:26580525-26582395	1871	4553	2956.7
Chr1:28199276-28206541	7266	123176	79989.9
Chr1:28209600-28216678	7079	66571	43230.9
Chr1:29052691-29057130	4440	29406	19096.1
Chr1:30807458-30812193	4736	12190	7916.12
Chr1:39322719-39324563	1845	4400	2857.34
Chr2:1292602-1294479	1878	3502	2274.18
Chr2:3700082-3702958	2877	4951	3215.15
Chr2:5899763-5907331	7569	86576	56222
Chr2:6758826-6764429	5604	35993	23373.7
Chr2:6795025-6797018	1994	25003	16236.8
Chr2:6797255-6798739	1485	14221	9235.05
Chr2:7603850-7612031	8182	49094	31881.4
Chr2:8343262-8350085	6824	53082	34471.2
Chr2:13580497-13582726	2230	8042	5222.43
Chr2:13582835-13584440	1606	21480	13949
Chr2:13584553-13586359	1807	18980	12325.5
Chr2:16555790-16558432	2643	3286	2133.91
Chr2:17352184-17362368	10185	3660	2376.79
Chr2:19469135-19471202	2068	33551	21787.9
Chr2:29633229-29636004	2776	7944	5158.79
Chr2:29777047-29782815	5769	19128	12421.6
Chr2:33666331-33670765	4435	4813	3125.54
Chr2:33847610-33850584	2975	6909	4486.67
Chr3:10131213-10135276	4064	4891	3176.19
Chr3:12709269-12710829	1561	5367	3485.3
Chr3:12711053-12713326	2274	5180	3363.87
Chr3:12967457-12976059	8603	11405	7406.35
Chr3:15609566-15614287	4722	7576	4919.82
Chr3:16165151-16169246	4096	4726	3069.04
Chr3:16790322-16793969	3648	6329	4110.02
Chr3:17534150-17543648	9499	20408	13252.9
Chr3:19353251-19356624	3374	3226	2094.95
Chr3:22402320-22411470	9151	84547	54904.4
Chr3:24408927-24413594	4668	3377	2193.01
Chr3:24566342-24574131	7790	87274	56675.3
Chr3:24600899-24607135	6237	29303	19029.2
Chr3:26123027-26126245	3219	28354	18412.9
Chr3:26143503-26145459	1957	3956	2569.01
Chr3:27617305-27621763	4459	33321	21638.5
Chr3:29748690-29757209	8520	17679	11480.7
Chr4:11651699-11659200	7502	10093	6554.34
Chr4:19681040-19685207	4168	25269	16409.6
Chr4:23337249-23341399	4151	4253	2761.88
Chr4:35049574-35053501	3928	77874	50571
Chr5:661501-664202	2702	4316	2802.79
Chr5:1132446-1140507	8062	57162	37120.7
Chr5:1679719-1685842	6124	24000	15585.5
Chr5:4824691-4831616	6926	39053	25360.8
Chr5:10916681-10920212	3532	36938	23987.4
Chr5:10920336-10923044	2709	15297	9933.79
Chr5:11242416-11246815	4400	96684	62786.1
Chr5:16055424-16058037	2614	21599	14026.3
Chr5:19884414-19887244	2831	5951	3864.55
Chr5:25347432-25352003	4572	9833	6385.5
Chr5:25352165-25357702	5538	137780	89473.6
Chr5:26871666-26874206	2541	26494	17205.1
Chr6:6074353-6077281	2929	3969	2577.45
Chr6:6078050-6083497	5448	44557	28935.1
Chr6:7380634-7384393	3760	5793	3761.95
Chr6:7534413-7539307	4895	59362	38549.4
Chr6:11299537-11305651	6115	60176	39078
Chr6:12034039-12041755	7717	6408	4161.32
Chr6:13424894-13438639	13746	171928	111649
Chr6:17320069-17323450	3382	10015	6503.69
Chr6:17672312-17677290	4979	4691	3046.31
Chr6:19708606-19712027	3422	12696	8244.72

Chr6:21224116-21229716	5601	7264	4717.21
Chr6:23399231-23400693	1463	3208	2083.26
Chr6:27797786-27809473	11688	86885	56422.7
Chr6:28515401-28518631	3231	12851	8345.37
Chr6:29376926-29380707	3782	72201	46887
Chr7:994259-1002410	8152	16333	10606.6
Chr7:3900402-3903667	3266	3445	2237.17
Chr7:4345533-4353177	7645	91811	59621.6
Chr7:4372338-4377228	4891	67854	44064
Chr7:5719312-5723719	4408	3708	2407.96
Chr7:8291249-8295114	3866	11836	7686.24
Chr7:8295238-8301025	5788	52317	33974.4
Chr7:12422204-12424303	2100	3166	2055.98
Chr7:15676996-15677884	889	6300	4091.19
Chr7:15677988-15679463	1476	5670	3682.07
Chr7:15712019-15717810	5792	24475	15893.9
Chr7:15986454-15991210	4757	45006	29226.7
Chr7:19997588-20006845	9258	24983	16223.8
Chr7:22459896-22462004	2109	4013	2606.02
Chr7:23519922-23522314	2393	14805	9614.29
Chr7:27168168-27170310	2143	8674	5632.85
Chr8:5774450-5782021	7572	25294	16425.8
Chr8:7662591-7666358	3768	4906	3185.93
Chr8:7667566-7673911	6346	23564	15302.3
Chr8:7762777-7770869	8093	76638	49768.3
Chr8:7989698-7992785	3088	3899	2531.99
Chr8:8446492-8449809	3318	3279	2129.37
Chr8:9442326-9449307	6982	26946	17498.6
Chr8:10491601-10502107	10507	76879	49924.8
Chr8:15382219-15386505	4287	4929	3200.87
Chr8:15452051-15462294	10244	193230	125483
Chr8:15463930-15466939	3010	19090	12396.9
Chr8:16972475-16979505	7031	31524	20471.5
Chr8:20978460-20986722	8263	36753	23867.2
Chr8:21691357-21695683	4327	25672	16671.3
Chr8:27144219-27147533	3315	20172	13099.6
Chr9:1285735-1297850	12116	16055	10426
Chr9:3227842-3229695	1854	4320	2805.39
Chr9:3229897-3231549	1653	4436	2880.72
Chr9:3511863-3520039	8177	3616	2348.21
Chr9:9556907-9558463	1557	5846	3796.36
Chr9:12418728-12427379	8652	12860	8351.22
Chr9:12621912-12624864	2953	9163	5950.41
Chr9:15228033-15232300	4268	13083	8496.03
Chr9:16723080-16727704	4625	86173	55960.3
Chr10:402209-405187	2979	5792	3761.3
Chr10:706457-709017	2561	15727	10213
Chr10:3440841-3446759	5919	25840	16780.4
Chr10:5923285-5934240	10956	111709	72543.3
Chr10:15985582-15993013	7432	69318	45014.8
Chr10:16003045-16005181	2137	19802	12859.3
Chr10:16005548-16007059	1512	29905	19420.2
Chr10:20760687-20764658	3972	6258	4063.91
Chr10:20776680-20779739	3060	21513	13970.4
Chr11:2540753-2545719	4967	19104	12406
Chr11:3230045-3233566	3522	39640	25742
Chr11:3993122-4001684	8563	3604	2340.42
Chr11:7333501-7335924	2424	10522	6832.93
Chr11:7720427-7725287	4861	4315	2802.14
Chr11:7725464-7730825	5362	11047	7173.87
Chr11:7731057-7735579	4523	98079	63692
Chr11:7735681-7745404	9724	109025	70800.3
Chr11:8909715-8916176	6462	13447	8732.41
Chr11:16329832-16336817	6986	146548	95167.5
Chr11:17164428-17170277	5850	26844	17432.4
Chr11:19254414-19256359	1946	4080	2649.53
Chr11:20706543-20709580	3038	27364	17770
Chr11:21410967-21413825	2859	7780	5052.29
Chr11:22338328-22343006	4679	8597	5582.85
Chr11:24386677-24394635	7959	125353	81403.6
Chr11:27016637-27019288	2652	16672	10826.7
Chr12:4726888-4738498	11611	160135	103991
Chr12:4898697-4904135	5439	52407	34032.8
Chr12:4909460-4920376	10917	67759	44002.4
Chr12:5022326-5028798	6473	20205	13121
Chr12:9061812-9066482	4671	12399	8051.85
Chr12:10926270-10929294	3025	4663	3028.13
Chr12:17499957-17504299	4343	75202	48835.8
Chr12:17505052-17508160	3109	3334	2165.08
Chr12:17984443-17989007	4565	34540	22430.1
Chr12:18391543-18392884	1342	6544	4249.64
Chr12:18392996-18394312	1317	7551	4903.58
Chr12:18394419-18397218	2800	8604	5587.39
Chr12:18860401-18868300	7900	51691	33567.9
Chr12:24725046-24735886	10841	167897	109031

Table S2 Basic information of high-throughput sequencing.

mRNA-seq basic information

sample	raw reads	raw bases	clean reads	clean bases	mapped reads	mapped ratio	depth (X)	SRA	reference
TP309 seedling-R1	59,359,372	8,903,905,800	56,306,542	8,350,925,760	53,403,841	94.84%	22.39		
TP309 seedling-R2	60,424,258	9,063,638,700	57,222,120	8,485,162,962	54,397,444	95.06%	22.75		
TP309 seedling-R3	71,100,710	10,665,106,500	68,268,376	10,159,956,972	64,941,139	95.13%	27.24		
TP309 inflorescence-R1	67,537,852	10,130,677,800	65,650,002	9,807,815,124	62,067,623	94.54%	26.29		
TP309 inflorescence-R2	70,342,390	10,551,358,500	68,459,800	10,228,000,942	64,711,656	94.53%	27.42		
Nip inflorescence-R1	69,906,792	10,486,018,800	63,796,400	9,332,604,980	60,771,007	95.26%	25.02	SRX3837322	Wang et al., 2022
Nip inflorescence-R2	67,044,098	10,056,614,700	60,326,862	8,813,623,755	57,151,392	94.74%	23.63	SRX3837323	Wang et al., 2022
osrdr2-3 -inflorescence-R1	69,885,574	10,482,836,100	65,013,666	9,648,288,625	60,313,287	92.77%	25.87	SRX3837325	Wang et al., 2022
osrdr2-3 -inflorescence-R2	67,544,644	10,131,696,600	59,756,218	8,724,920,377	55,205,212	92.38%	23.39	SRX3837326	Wang et al., 2022
Nip seedling-R1	274,272,624	41,140,893,600	251,471,788	37,210,950,150	237,216,298	94.33%	99.76	SRX5724238	Wang et al., 2022
Nip seedling-R2	125,356,904	18,803,535,600	115,313,972	17,036,610,626	110,542,360	95.86%	45.67	SRX5724239	Wang et al., 2022
Nip stamen-R1	79,193,822	11,879,073,300	76,682,404	11,445,718,761	73,313,209	95.61%	30.69	SRX8512913	Wang et al., 2022
Nip stamen-R2	84,400,444	12,660,066,600	81,429,974	12,151,494,381	77,489,483	95.16%	32.58	SRX8512914	Wang et al., 2022
Nip stamen-R3	88,647,982	13,297,197,300	85,699,916	12,790,464,730	81,727,019	95.36%	34.29	SRX8512915	Wang et al., 2022
osrdr2-3 stamen-R1	78,100,906	11,715,135,900	75,756,582	11,313,273,498	72,599,932	95.83%	30.33	SRX8512916	Wang et al., 2022
osrdr2-3 stamen-R2	94,160,166	14,124,024,900	91,096,730	13,600,442,830	86,939,598	95.44%	36.46	SRX8512917	Wang et al., 2022
osrdr2-3 stamen-R3	84,001,516	12,600,227,400	81,080,748	12,101,849,943	77,181,083	95.19%	32.44	SRX8512918	Wang et al., 2022
Nip pistil-R1	67,864,086	10,179,612,900	65,739,068	9,813,460,640	63,080,665	95.96%	26.31	SRX8512922	Wang et al., 2022
Nip pistil-R2	85,546,240	12,831,936,000	82,628,330	12,333,614,112	78,932,663	95.53%	33.07	SRX8512923	Wang et al., 2022
Nip pistil-R3	82,711,026	12,406,653,900	79,988,436	11,941,312,616	76,239,247	95.31%	32.01	SRX8512924	Wang et al., 2022
osrdr2-3 pistil-R1	60,308,434	9,046,265,100	58,377,330	8,716,684,070	56,081,979	96.07%	23.37	SRX8512925	Wang et al., 2022
osrdr2-3 pistil-R2	76,990,078	11,548,511,700	74,266,718	11,087,236,958	71,126,258	95.77%	29.72	SRX8512926	Wang et al., 2022
osrdr2-3 pistil-R3	71,775,126	10,766,268,900	69,145,974	10,324,886,034	65,985,379	95.43%	27.68	SRX8512927	Wang et al., 2022

sRNA-seq basic information

sample	raw reads	clean reads	mapped reads	mapped ratio	SRA	reference
Nip bract	19,988,796	19,969,562	15,691,409	78.58%		
Nip branch	21,506,629	21,496,916	18,451,096	85.83%		
TP309 seedling-R1	28,321,200	28,311,900	22,542,144	79.62%	SRX11502899	Wang et al., 2022
TP309 seedling-R2	27,980,509	27,965,218	21,968,483	78.56%	SRX11502900	Wang et al., 2022
TP309 inflorescence-R1	20,740,453	20,739,517	16,952,014	81.74%		
TP309 inflorescence-R2	28,674,331	28,674,085	19,137,666	66.74%		
TP309 seed coat	24,149,326	24,149,325	20,975,097	86.86%		
fem1-1 seed coat	21,981,245	21,981,243	18,383,150	83.63%		
pol iv seed coat	21,382,456	21,382,453	16,669,784	77.96%		
TP309 endosperm	22,388,240	22,388,240	18,459,636	82.45%		
fem1-1 endosperm	21,483,482	21,483,481	18,128,932	84.39%		
pol iv endosperm	22,421,276	22,421,275	17,743,305	79.14%		
Nip inflorescence-R1	25,030,595	25,030,251	16,847,503	67.31%	SRX3837382	Wang et al., 2022
Nip inflorescence-R2	27,227,675	27,227,004	23,102,469	84.85%	SRX5724233	Wang et al., 2022
osrdr2-3 inflorescence	23,490,085	23,489,603	14,365,213	61.16%	SRX3837383	Wang et al., 2022
osrdr2-6 inflorescence	27,978,716	27,977,683	19,715,066	70.47%	SRX5724234	Wang et al., 2022
seedling-R1	24,875,329	24,875,202	21,678,829	87.15%	SRX5724235	Wang et al., 2022
seedling-R2	25,131,542	25,131,495	21,913,601	87.20%	SRX5724236	Wang et al., 2022
osrdr2-6 seedling	24,737,577	24,737,425	19,747,442	79.83%	SRX5724237	Wang et al., 2022
Nip stamen	21,045,563	21,038,047	16,305,153	77.50%	SRX8512935	Wang et al., 2022
osrdr2-3 stamen	24,307,925	24,307,719	20,474,882	84.23%	SRX8512936	Wang et al., 2022
osrdr2-6 stamen	25,967,101	25,966,752	21,961,584	84.58%	SRX8512937	Wang et al., 2022
Nip pistil	19,986,635	19,985,410	17,356,918	86.85%	SRX8512938	Wang et al., 2022
osrdr2-3 pistil	26,506,662	26,504,142	21,282,404	80.30%	SRX8512939	Wang et al., 2022
osrdr2-6 pistil	26,048,327	26,048,255	22,596,967	86.75%	SRX8512940	Wang et al., 2022
egg cell-R1	34,338,087	17,949,255	13,431,907	74.83%	SRX5757720	Li et al., 2020
egg cell-R2	36,755,974	21,298,630	15,828,862	89.91%	SRX5757723	Li et al., 2020
ovary without the egg cell-R1	40,408,939	30,862,239	27,747,170	77.50%	SRX5757668	Li et al., 2020
ovary without the egg cell-R2	42,307,958	32,635,862	29,331,685	79.10%	SRX5757691	Li et al., 2020
sperm-R1	52,745,236	32,007,855	24,806,059	74.32%	SRX5757716	Li et al., 2020
sperm-R2	60,979,419	37,833,278	29,345,251	89.88%	SRX5757717	Li et al., 2020
vegetative cell-R1	118,005,472	41,020,785	32,447,006	77.56%	SRX5757653	Li et al., 2020
vegetative cell-R2	86,675,388	34,093,634	26,449,728	77.58%	SRX5757654	Li et al., 2020
embryo	26,233,129	24,712,273	20,608,555	83.39%	SRX248516	Rodrigues et al. 2013
endosperm	22,605,850	19,253,861	16,030,242	83.26%	SRX248515	Rodrigues et al. 2013

Basic information of bisulfite sequencing data.

sample	raw reads	raw bases	clean reads	clean bases	mapped reads	mapped ratio	coverage	depth (X)	conversion rate	SRA	reference
Nip inflorescence-R1	157,087,444	15,708,744,400	148,033,072	14,803,307,200	102,959,700	69.6%	93.54%	22.79	99.55%	SRX3837328	Wang et al., 2022
Nip inflorescence-R2	168,633,068	25,294,960,200	161,320,954	24,198,143,100	106,198,762	65.8%	94.18%	28.10	99.58%	SRX5724228	Wang et al., 2022
osrdr2-3 inflorescence	159,396,562	15,939,656,200	151,230,624	15,123,062,400	103,639,178	68.5%	93.96%	23.10	99.62%	SRX3837329	Wang et al., 2022
osrdr2-6 inflorescence	140,415,006	21,062,250,900	134,758,396	20,213,759,400	83,582,372	62.0%	92.86%	21.93	99.44%	SRX5724229	Wang et al., 2022
Nip seedling-R1	227,476,972	34,121,545,800	218,857,910	32,828,686,500	168,637,380	77.1%	94.59%	35.33	99.53%	SRX5724230	Wang et al., 2022
Nip seedling-R2	178,621,232	26,793,184,800	171,791,384	25,768,707,600	132,069,884	76.9%	94.05%	29.42	99.51%	SRX5724231	Wang et al., 2022
osrdr2-6 seedling	243,416,222	36,512,433,300	234,162,858	35,124,428,700	182,309,254	77.9%	94.70%	39.09	99.55%	SRX5724232	Wang et al., 2022
Nip stamen	135,381,864	20,307,279,600	135,381,806	20,307,270,900	109,532,276	80.9%	94.02%	25.52	99.46%	SRX8512907	Wang et al., 2022
osrdr2-3 stamen	134,734,552	20,210,182,800	134,734,156	20,210,123,400	99,740,148	74.0%	94.24%	26.94	99.54%	SRX8512908	Wang et al., 2022
osrdr2-6 stamen	135,399,670	20,309,950,500	135,399,612	20,309,941,800	106,647,162	78.8%	93.83%	25.09	99.49%	SRX8512909	Wang et al., 2022
Nip pistil	134,834,618	20,225,192,700	134,834,560	20,225,184,000	106,989,728	79.3%	93.79%	24.71	99.42%	SRX8512910	Wang et al., 2022
osrdr2-3 pistil	135,111,284	20,266,692,600	135,111,240	20,266,686,000	107,675,592	79.7%	93.74%	25.36	99.45%	SRX8512911	Wang et al., 2022
osrdr2-6 pistil	135,271,462	20,290,719,300	135,271,390	20,290,708,500	104,106,846	77.0%	93.69%	23.59	99.46%	SRX8512912	Wang et al., 2022
endosperm	72,549,332	10,882,399,800	63,416,348	9,512,452,200	51,983,520	82.0%	82.46%	12.98	99.49%	SRX4396080	Liu et al., 2018

Basic information of ChIP-seq data

sample	raw reads	clean reads	mapped reads	mapped ratio	SRA	reference
young inflorescence H3K9me2	36,040,908	33,849,332	33,728,693	99.64%	SRX7426653	Zhao et al., 2020
young inflorescence H3K27me3	31,084,746	28,068,766	27,277,132	97.18%	SRX7426649	Zhao et al., 2020
young inflorescence H3K4me1	21,003,844	18,872,928	17,328,304	91.82%	SRX7426651	Zhao et al., 2020
young inflorescence H3K4me3	17,702,958	15,326,724	15,118,170	98.64%	SRX7426645	Zhao et al., 2020
young inflorescence H3K27ac	19,577,316	16,782,264	16,505,881	98.35%	SRX7426647	Zhao et al., 2020
young inflorescence Pol II	18,681,528	16,208,338	15,708,263	96.91%	SRX7426655	Zhao et al., 2020
endosperm H3K27me3	10,999,930	10,925,764	7,287,778	66.70%	SRX040548	Malone et al., 2011

Table S3 Primers list.

Name	Sequence(5'-3')	Experiment
BILL-F	GATGGAGAGAGTATTTGTGTAC	sRNA northern blotting (Corresponding to Figure 4F)
BILL-R	GTCTCTCTAGCAGCGTAAAGC	sRNA northern blotting (Corresponding to Figure 4F)
methyl1-F	CATTTTGCTTCCTGCATTCCTC	Chop-PCR (Corresponding to Figure 3D the first locus)
methyl1-R	CTGGCGTGCAACCGCACAACATG	Chop-PCR (Corresponding to Figure 3D the first locus)
methyl2-F	GATGGAGAGAGTATTTGTGTAC	Chop-PCR (Corresponding to Figure 3D the second locus)
methyl2-R	GTCTCTCTAGCAGCGTAAAGC	Chop-PCR (Corresponding to Figure 3D the second locus)
